# Neoplasia in the dromedary camel: a review (*Camelus dromedarius*)

**DOI:** 10.3389/fvets.2025.1664874

**Published:** 2025-12-10

**Authors:** Hassan Abu Damir, Mohamed H. Tageldin, Mahmoud A. Ali, Abdu Adem

**Affiliations:** 1 Department of Medical Sciences, College of Medicine and Health Sciences, Khalifa University, Abu Dhabi, United Arab Emirates; 2 College of Agricultural and Marine Sciences, Sultan Qaboos University, Muscat, Oman

**Keywords:** dromedary camels, tumors types and body location, tumor relative frequency, risk factors, current and future tumor diagnosis, future tumor treatment

## Abstract

**Background:**

Dromedary camels are a source of milk, meat, wool, and income in poor societies and play an important role in social events. Tumors have devastating effects on the health, production, reproduction, and marketing value of dromedaries. Reviews on neoplasia in dromedaries are scarce, making it pertinent to present this comprehensive study.

**Aim:**

This review provides insights into the epidemiology, pathology, future diagnosis, and treatment of camel tumors.

**Methods:**

The literature on tumors in dromedaries is reviewed with a focus on tumor types in different body systems, epidemiology, risk factors, future diagnosis, and treatment. Epidemiological data were collected from various sources, analyzed, and presented according to body systems. The relative frequencies of malignant and benign tumors were displayed.

**Results:**

The relative frequencies of tumors in camels were as follows: skin (54.8%), reproductive (23.0%), lymphoid (6.9%), and gastrointestinal (6.3%). The common malignant and benign tumors were observed in the following order: carcinomas (35.5%), fibromas (11.2%), teratomas (7.8%), lymphomas and leukemia (7.4%), papillomas (6.2%), and adenomas (6.2%). A recent rise in tumor cases was observed. Possible risk factors included contamination of feed with fungal toxins and exposure to pesticides, herbicides, hydrocarbons, and heavy metals from the petroleum industry and gold mining. Other risk factors included environmental changes or famine, certain veterinary drugs and hormones, plant carcinogens, ultraviolet light, familial predisposition, and old age. The contraction of bovine papillomavirus (PV) during movement to new areas with denser animal populations is also likely.

**Conclusion:**

Different tumor types were reported in various body systems of the dromedary, with carcinomas, fibromas, teratomas, lymphomas/leukemia, and papillomas being the most common. An increase in tumor reports in camels is expected due to recent advancements in camel farming systems, specialized breeding centers, improvements in veterinary services, and enhanced disease surveillance. Some skin tumors, such as papillomas/fibropapillomas, exhibited koilocytosis, possibly caused by different PV strains, which may warrant further investigation. Abattoir tumor surveys may show bias, as dromedaries presented for slaughter were typically barren females or young males. Various diagnostic methods were discussed, and future advanced technologies for tumor diagnosis and treatment in camels, including targeted therapy and precision medicine, were suggested.

## 1 Introduction

The majority of the dromedary camels in the world are in Somalia, Sudan, Eritrea, and Ethiopia. Fewer are found in the Arabian Peninsula, India, and Pakistan (1, 2). Dromedaries are of social and economic importance as sources of milk, meat, wool, and cash, and they are also a source of pride in racing and beauty pageant events. Tumors result from a series of genetic mutations where cellular carcinogenesis progresses through stages of initiation, promotion, and finally progression (3). The changes leading to cancer include DNA point mutations, chromosomal rearrangements, epigenetic changes in DNA packing into the nucleus, and the formation of multiple copies of “on switch” points near genes (4, 5). All types of cells derived from the embryonic layers—ectoderm, mesoderm, and endoderm—are subject to neoplastic changes (4, 5). Both benign and malignant tumors have been reported in dromedaries, as in other domestic animals (6–8). Malignant tumors can be well-differentiated, moderately differentiated, poorly differentiated, or undifferentiated/anaplastic, and they may be primary, metastatic, or invasive (4, 6). Tumors can grow anywhere in the body of the one-humped camel, as in other species, and have a direct impact (9, 10). For example, skin tumors mar the skin of valuable camels and devalue their price; joint and toenail tumors hinder movement and racing; sternal pad tumors impede resting (9); and odontogenic tumors affect browsing. Large neoplasms in the reproductive system have been reported to affect breeding, and sex-cord-stromal tumors have been associated with sexual behavioral changes and infertility (7, 11). Neoplasia may cause lethargy, anemia, decreased immunity, and mortality (9, 12). Grossly, tumors exhibit variable colors, sizes, and shapes. They can be soft or hard, single lesions or multiple nodular masses, and have either a cauliflower or smooth surface. The lesions may be bulging on the surface or deeply embedded in tissues. Neoplasia can be encapsulated with a thick fibrous or loose capsule, or may be non-encapsulated (4, 12). Tumor cells acquire physiological, biochemical, histopathological, and anatomical characteristics associated with the secretion of factors promoting the tumor microenvironment (13). The tumor microenvironment includes stromal fibroblastic tissue, blood vessels, immune cells, signaling molecules, and extracellular matrix. These components significantly facilitate or impede tumor growth, metastasis, and invasion (14, 15). Tumor cells are closely impacted and exhibit different cytoplasmic colors and nuclear shapes, with prominent nucleoli and varying mitotic figures, as well as differing cellular characteristics compared to normal cells (16). The cellular characteristics and atypia are predictive of tumor type and differentiation.

The future prospects for dromedaries in developed Arab countries appear promising due to advancements in intensive farming, disease treatment and control, surveillance programs, and selective breeding for milk, meat, and racing purposes. On the other hand, famine, especially in the Horn of Africa, has forced nomads to move with their camels from their natural habitat to cultivated and industrial areas. The extensive use of herbicides and insecticides, along with the hazardous waste from gold mining and petroleum activities, can pollute the air, pasture, and water with carcinogenic compounds and heavy metals. Additionally, these camels have to share common grazing areas and water points with other domestic and wild animals, which increases the risk of contracting infections, especially papillomaviruses (PV) (17–22).

Given the limited number of reviews on camel tumors, this comprehensive work is presented to help fill this knowledge gap. The review focuses on the epidemiology, pathology, diagnosis, and treatment of dromedary camel tumors. Our objective was to describe tumor types and their relative frequencies to provide deeper insights into the understanding of tumors in dromedary camels. Furthermore, the review aims to provide easy references for veterinarians, researchers, and camel owners to assist with the diagnosis and recording of tumors in field cases. The review also seeks to raise awareness among authorities and policymakers about the potential increase in tumor cases because of environmental changes and the risk factors that may contribute to tumor occurrence.

## 2 Relative frequency of tumor in the dromedary camels

There have been reports showing low neoplastic incidence rates in camels (10); however, we believe that the reporting of tumor incidence in camels is below the actual cases. This may be due to the reluctance of owners and field veterinarians to report. The selective reporting of tumor incidence in certain organs, such as the ovaries, liver, skin, and eyes, during abattoir surveys could be useful (Table 1) but may provide biased information, as most slaughtered camels were young males or barren females (6, 23). Generally, tumor diagnosis and reporting are expected to increase with the recent advancements in camel husbandry and owners' awareness.

**Table 1:** Some tumor surveys in the dromedary camels: country, total number surveyed, sex and age, total reported cases, type of tumors, % incidence, references and reference number.

**Investigation**	**Country and source**	**Total no**	^*^Sex: *m, f* Age: *y*	**Reported cases**	**Type of tumors**	**% incidence**	**References**
General tumor incidence	KSA (clinic)	9,576	*m, f;* 4 month−18 y	59	Different types	0.006%	(10)
**Skin and appendages**							
Cutaneous lesions	Iran (abattoir)	105	Sex/age not defined	16 skin lesions	Six tumor types	15.24%	(114)
Skin and S/C tissue	Egypt (abattoir)	988 (808 *m*; 180 *f*)	*m, f*, 4–15 y	13 skin lesions	Papilloma (0.1%), fibropapilloma (0.1%), lipomas (0.2%), adenoma (0.1%), squamous cell carcinoma (0.1%), and myxosarcomas (0.7%)	1.3%	(257)
**Reproductive system**							
Genital tract	KSA (clinic)	447	*f*	2	Adenocarcinoma, lipoma	0.5%	(23)
Genital tract	Iraq (abattoir)	80	*f*	2	ovarian cystadenoma (1.25%) ovarian papilloferous cystadenoma (1.25%)	2.5%	(237)
Genital tract	KSA (Clinic)	1,621	*f*, 9–13 years	12	Adenocarcinoma	0.007%	(161)
Genital tract	Algeria. (abattoir)	165	*F*, adult	1	Ovarian teratoma	0.6%	(220)
Genital tract	Egypt (abattoir)	500	*F*, adult	7	Demoid cyst (0.4%); arrhenoblastoma (0.2%); leiomyoma (0.4%); Lipoma (0.2%); adenocarcinoma (0.4%)	1.4%	(150)
Genital tract	Ethiopia (abattoir)	140	*f, adult*	0	No tumors	0%	(353)
Ovaries	Ethiopia (abattoir)	231	*f, adult*	2	*Hemangioma*	0.87%	(238)
Ovaries	KSA (abattoir and clinic)	600	*f, adult*	2	1 teratoma (0.17%), 1 adenoma (0.17%).	0.34%	(223)
Ovaries	UAE (abattoir)	531	*f*, >16 year	10	Teratomas	1.9%	(216)
Ovaries	Egypt (abattoir)	500	*f*, 6–15 years	34	Papillary cystadenoma (0.6%), fibroadenoma (0.2%), granulosa cell tumor (1.2%), luteoma (0.8%), thecoma and luteinised thecoma (0.4%), fibrothecoma (0.4%), teratoma (2%), fibroma (1.6%), cavernous haemangioma (0.4%); mixed tumors (0.6%)	6.8%	(117)
Ovaries	Egypt (abattoir)	180	*f, adult*	15	Sex cord-stromal tumors: adult granulosa cell tumor (0.55%); interstitial cell tumor (0.55%); steroid cell tumor-NOS (0.55%); thecoma (1.1%); fibrothecoma (1.1%); Granulosa-theca cell tumor (5.55%)	8.30%	(11)
Mammary neoplasia	Sudan (abattoir)	150	*f, adult*	45	Benign (15.4%); malignant (14.6%). papillary carcinoma and fibroadenoma	30%	(116)
**Digestive system**							
Liver	Egypt (Abattoir)	10,000	*m*; *f*	6	Bening (3) malignant (3) leiomyoma (1), haemangioma (2), cholangio carcinoma (2), hepatocellular carcinoma (1),	0.006%	(155)
Liver	KSA (Abattoir)	500	*m*; *f*	4	Bening (3) malignant (1)	0.8%	(157)
Liver	Egypt (Abattoir)	988	*m* (808); *f* (180),	7	Unusual multiple primary liver tumors (5 types and 7 cases)	0.70%	(151)
Liver	Iran (Abattoir)	70	Not specified	5	Lipoma, 1, (5%); cavernous hemangioma 1 (1.4%); leiomyoma 1 (1.4%)	7.1%	(113)
**Respiratory system**							
Lungs	Sudan (Abattoir)	45	*F, adult*	4	Pulmonary papillary carcinoma	8.9%	(115)

^*^Sex: m, male; f, female.

Age: y, years.

There were no records to check for tumors in most private camel farms and small veterinary clinics. However, these records were mostly available in university clinics and governmental veterinary hospitals and laboratories. In this review, a total of 682 tumor cases were identified in the literature (cumulative frequency). We reported the cumulative frequency and relative frequency of different malignant and benign tumors in the various body systems of the dromedary camel (Supplementary Figure S1), with results displayed in chronological order. The skin and integument showed the highest relative frequency (54.84%) of tumors, followed by the reproductive system (23.02%); lymphatic and hematopoietic system (6.89%); digestive system (6.3%); respiratory system (3.81%); musculoskeletal system (2.49%); urinary system (1.47%); nervous system (0.59%); and endocrine system (0.59%). The relative frequency of malignant tumors is presented in Supplementary Figure S2. Carcinomas showed the highest relative frequency (35.45%), followed by lymphomas and leukemia (7.4%); sarcomas (4.8%); germ cell tumors (0.59%); primitive ectodermal tumors (0.44%); sex cord stromal tumors (0.29%); melanomas (0.29%); mast cell tumors (0.15%); and nephroblastomas (0.15%). On the other hand, the relative frequency of benign tumors in dromedary camels is presented in Supplementary Figure S3. Fibromas showed the highest relative frequency (11.6%), followed by teratomas (7.68%); papillomas (6.17%); adenomas (6.17%); papillomatosis (4.7%); hemangiomas (3.67%); sex cord stromal tumors (3.08%); lipomas (2.20%); keratodermas (2.2%); leiomyomas (1.76%); osteomas (0.44%); melanocytomas (0.44%); myxomas (0.29%); schwannomas (0.29%); ameloblastomas (0.15%); and pyogranulomas (0.15%).

The incidence rates of dromedary tumors (Table 1) were high in Sudan (25 [30%]; 26, [8.9%]); Egypt (27 [6.8%]; 11 [8.30%]); Iran (28 [7.1%], 29 [15.24%]); and Iraq (30 [2.5%]) compared to other countries. However, these incidences were relatively low in Saudi Arabia (31 [0.34%], 10 [0.006%], 24 [0.5%], 13 [0.007%]); Algeria (32 [0.6%]); and Ethiopia (33 [0%]). A number of tumor risk factors (24, 25) might be implicated in these high tumor incidences in certain countries.

## 3 Tumor risk factors

There are several risk factors that provoke neoplasia in camels and other species, and knowledge of these can lower cancer risk. These factors include fungal toxins, pesticides, environmental pollutants, pharmaceutical drugs and hormones, certain viruses, ultraviolet light, age, and genetic inheritance (10, 26). Supplementary Table S1 illustrates some of the tumor risk factors that could be acquired or inherited, along with possible mechanisms of carcinogenesis (26).

### 3.1 Mycotoxins

The high temperature and humidity in arid and semi-arid environments favor fungal growth in animal feed (27). Fungal toxins, such as Aflatoxin B1, are implicated in hepatocellular carcinoma and lung adenocarcinoma; Ochratoxin is associated with renal cell, liver, and gall bladder carcinoma (28); Zearalenone induces hormonal activity with possible genotoxicity and/or carcinogenicity in the reproductive organs. Ochratoxin and other mycotoxins are also implicated in cancer. The maximum permissible limit of mycotoxin in animal feed is 20 ppb (29, 30). Different types of mycotoxins, including Aflatoxins, Ochratoxin A, and Zearalenone, were isolated and quantified in stored camel feed (31, 32). Ingested mycotoxins can accumulate in various body organs (33), primarily in muscle, liver, kidney, mammary gland, and the nervous, endocrine, and immune systems (34–36). High aflatoxin B1 residues were reported in 23.4% of camel livers, accompanied by fibrosis and large whitish lesions (37). Histopathology revealed cholangitis, cirrhosis, bile duct obstruction, and hepatic carcinoma. Mycotoxins were also a public concern in camel meat in Australia (36). Moreover, Aflatoxin B1 is converted to various metabolites, including Aflatoxin M1, by cytochrome P450 and associated enzymes, which can be secreted in milk (38). Both Aflatoxins B1 and M1 are risk factors for hepatic carcinoma (30). 29% of dromedary milk samples were reported to contain aflatoxin M1 levels (39) exceeding the GCC limit (200 ng/L) (40). Animal feed should not be stored for long; regular analysis should be performed, and highly contaminated feed must be disposed of.

### 3.2 Agricultural pesticides and herbicides

Pesticides and herbicides are widely used to control insects and weeds; however, they may pose health hazards (41, 42). Famine forced camels to move to more cultivated lands where they could consume residues of potential carcinogenic pesticides in fodder, remnants of agricultural harvests, contaminated soil and water, or by accidental access to chemical storage areas. Additionally, in the Gulf, these pesticides are stored within camel farms for use in deterring biting flies, which presents occasional hazards. The potentially carcinogenic agricultural insecticides include organochlorine pesticides such as DDT, Gamatox, chlordane, Heptachlor, Aldrin, Dieldrin, and others, with bioaccumulation of their residues in camel meat (43, 44). Furthermore, some organophosphate pesticides, such as chlorpyrifos, and organic compounds such as carbamates, bipyridine pesticides, and chlorinated or brominated herbicides (Paraquat, Diquat) as well as triazine herbicides (Atrazine, Simazine) are potentially carcinogenic (45–47). The fungicides with potential carcinogenicity used in agriculture include benzimidazole, Maneb, Zineb, and phthalimide (48, 49). The body organs most affected by pesticide carcinogens include the liver, lungs, brain, and blood. In general, the risk of non-Hodgkin's lymphomas increases with pesticide exposure (50). Awareness of the carcinogenicity of some agricultural chemicals is vitally important to protect against or reduce tumor incidences.

### 3.3 Pharmaceutical drugs

Many drugs are used for the treatment of camel diseases. Drug-related carcinogenesis and/or mutagenesis in the dromedary includes Nitroimidazoles, Nitrofurans, carbamates, Cyclosporine, Barbiturates, phenol pesticides, Phenylbutazone, carbon tetrachloride, hexachloroethane, trichloroethane, and nitrite (12, 51, 52). Moreover, Diazinon, dieldrin, chlorpyrifos, Buparvaquone, Sulfonamides, and Ivermectin (with chronic exposure) have been identified as carcinogens or potential carcinogens (53–57). Other drugs used in camels have been categorized as likely human carcinogens, such as Carbaryl, inclusive carcinogens like synthetic pyrethroids, or anticipated carcinogens such as Chloramphenicol (58–60). The dromedary has a slower clearance and longer elimination half-life for drugs (61, 62) and lower levels of drug-metabolizing enzymes, including P450 in the liver, which are responsible for the biotransformation of drugs into safer compounds (63–65). For these reasons, the dromedary has been reported to be more susceptible to drug toxicity compared to other species (66, 67). Therefore, we speculate that the dromedary is more prone to tumors induced by drugs. The use of carcinogenic or potentially carcinogenic pharmaceutical drugs, or those listed under controlled substances, is not recommended if safer alternatives are available.

### 3.4 Hormones

High levels of sex steroid hormones have been implicated in driving all stages of carcinogenesis through progesterone (PR) and estrogen (ERα and ERβ) receptors (68, 69) with potential carcinogenic effects (70). Hormonal therapy for *in vitro* fertilization (IVF) has been introduced by camel breeding centers to reduce the high infertility rates in this species. The IVF practice in camels is similar to the treatment of infertility in females through prolonged use and/or high doses of xenoestrogens/phytoestrogens. This is achieved by delivering progesterone and estradiol benzoate through an intravaginal device for an extended period (71, 72) or by injecting progesterone-in-oil (progestin) for 10–15 days along with gonadotropin treatment (73). Hormones used in IVF are known risk factors for ovarian carcinoma in women (70). Hence, therapies such as Diethylstilbestrol (synthetic estrogen) present a potential risk for clear-cell adenocarcinoma of the vagina and cervix (74), while synthetic progestogens (progestin/lynestrenol) may increase the risk of breast cancer (75), and HCG can influence endometrial adenocarcinoma and breast cancer formation and metastasis (76). Moreover, there are widespread practices of illegal use of anabolic steroids (AAS) such as Stanozolol analogs (tanozolol) during camel racing (12). These drugs could cause substantial DNA damage, with an increased risk of liver, testicular, prostate, and colorectal cancers (77). In addition, corticosteroids (cortisone, prednisolone, methylprednisolone, dexamethasone, betamethasone, and hydrocortisone) are commonly used drugs in the treatment of diseases in the dromedary (78). Long-term use of glucocorticoids could be a risk factor for overall cancer, especially that of the liver and lungs, or may act as predisposing factors for hormone-induced cancers (79). IVF practices must be under close surveillance to evaluate their potential hazards. Anabolic steroids (AAS) for veterinary use must also be classified as controlled substances, and their use in racing events should be discontinued.

### 3.5 Carcinogenic/genotoxic plants

The dromedary camels browse on more than 332 shrubs and trees, and to a lesser extent, graze on grasses (80). Many toxic plants are suspected to be carcinogenic and genotoxic; however, there are no reports of cancer cases attributed to these plants in the Arabian camel. Plants growing in camel habitat that act as risk factors for cancer include *Senecio, Crotalaria, Echium plantagineum, and Heliotropium europaeum*, which contain pyrrolizidine alkaloids, a well-known group of carcinogens (81, 82). *Aristolochia bracteata* and *Aristolochia fangchi* contain aristolochic acid and aristolochine, both of which are carcinogenic (81, 83). *Aloe barbadensis Miller*, a native plant of the Arabian Peninsula, contains anthraquinone and saponins, which are known genotoxic and carcinogenic compounds (84). *Croton* plants and seeds, an evergreen flowering shrub consumed by camels when accessible, contain the carcinogen phorbol esters (83, 85). *Trema tomentosa* and *Acacia nilotica* are very rich sources of tannins (86–88), and their toxicity has been reported in dromedaries (87, 89). Tannins and tannic acid are alleged to be carcinogenic (83). *Ricinus communis* produces castor seeds that contain ricin, with toxicity reported in dromedaries (90). Castor oil is anticipated to be a likely human carcinogen (91, 92). *Peganum harmala* is extensively used to purge camels just before racing in the Gulf region. The plant has different alkaloids that are teratogenic and genotoxic (93, 94). *Indigofera* plants contain indospicine and 2-aminopimelic acid, which cause chronic hepatotoxicity in feral camels and accumulate in meat, contaminating the food chain (95–97). Chronic hepatic toxicity is sometimes associated with cancer. The plant is highly cytotoxic but has not been tested for genotoxicity. *Calotropis procera*, a laticiferous evergreen plant with many therapeutic activities at lower doses and low toxicity (98, 99), contains latex with steroidal components and soluble laticifer proteins (SLPs) that are genotoxic and mutagenic *in vitro* at higher doses (99). Phytoestrogenic plants consumed by dromedaries, which may pose a carcinogenic risk, include soybean (genistein), alfalfa (coumestrol), and whole grains (matairesinol lignan). Pods of popular plants in the Gulf area, like *Prosopis cineraria* (ghaf) and *P. chilensis* (mesquite), are consumed by dromedary camels, having low piperidine alkaloid toxicity but containing phytoestrogenic effects similar to isoflavones daidzein and genistein in male and female Wistar rats (100). Additionally, plants that may pose a carcinogenic risk to dromedaries include *Pteridium aquilinum* (Bracken fern), which contains ptaquiloside; *laburnum* shrubs containing thiourea*; Dipteryx odorata, Cassia actifolia*, and *Galium odoratum*, which contains coumarin; and *cycad* trees containing cycasin and macrozamin. Some *Euphorbia* species produce latex containing diterpene derivatives/phorbol esters (81, 101, 102), with reports of toxicity in dromedaries (103). *Citrullus colocynthis* is toxic in high doses (81), and the seed tar is used by nomads to treat dromedary wounds; it has been shown to be carcinogenic to mouse skin after long exposure (104). *Jatropha curcas* has been endorsed as an oilseed crop; however, it is also toxic and carcinogenic due to phorbol esters (105). Nanoparticle technology may one day be able to utilize some compounds from the abovementioned plants or others to develop drugs for cancer treatment. *Citrullus colocynthis* is one example (81).

### 3.6 Gold mining

Mercury and cyanide are extensively used in gold mining (106, 107), resulting in the generation of toxic quantities of heavy metal waste including lead (Pb), arsenic (As), cadmium (Cd), chromium (Cr), and nickel (Ni), as well as other trace elements and sulfate ions (48). As, Cd, Ni, and Cr are classified as carcinogens and mutagens implicated in a wide range of cancers (108–110). Additionally, gold mining increases health and environmental hazards by generating dust and airborne pollutants that are carried far away by the wind. Industrial dust and large quantities of CO_2_ emitted contribute to ozone destruction, global warming, and cancer. Acidic waste also escapes into ground and surface water through rain, causing contamination of rivers, soil, and accumulation in crops, grasses, shrubs, and trees (107). Gold mining is practiced in many countries populated with camels, such as Sudan, Northeast Somalia, Iraq, and Iran (111). In Iran, for example, Asli et al. (112) reported higher heavy metal (As, Cr, and Pb) concentrations in dromedary livers and meat, along with other reports of high tumor incidence in Iranian camels (113, 114). Similarly, uncontrolled gold mining occurs in rampant areas in Sudan, leading to severe contamination of soil, air, water, and pasture (25), with high levels of cancer (Table 1) in locally slaughtered and exported Sudanese camels (11, 115–117). Haphazard gold mining and environmental pollution are serious issues in many countries and must be strictly regulated.

### 3.7 Petroleum and byproducts

Crude petroleum oil operations can cause environmental contamination, as volatile hydrocarbons lead to air pollution during drilling. Crude oil and formation water pumped out with oil and dumped into small ponds can cause substantial contamination with heavy metal carcinogens (Pb, As, Cd, Cr, Ni, and Hg) (24). Thus, oil extraction processes lead to groundwater and river contamination, with water-soluble hydrocarbons overflowing from ponds and exacerbated by rain, dispersing into open fields and streams, resulting in widespread pollution of soil, agricultural land, and grazing areas. Moreover, heavy crude petroleum components such as benzo(a)pyrene persist in areas of oil extraction. Some fractions of Total Petroleum Hydrocarbons have been implicated in cancer through inhalation, oral exposure, and skin contact after long-term exposure (118, 119). Petroleum oil byproducts such as benzene and benzo(a)pyrene are classified as carcinogens, while 1,3-butadiene is considered a probable carcinogen and acetaldehyde as a possible carcinogen (120), constituting risk factors for leukemia, lymphomas, and lung cancers. Sudan is heavily populated with livestock, including camels; however, wastewater extracted with oil has been indiscriminately disposed of in ponds and near large river delta, resulting in extensive area contamination and serious impacts on human and animal health (24, 121).

Plastic waste has been widely distributed by wind in the Sahara of some Arab countries (12, 122, 123). A large number of camels, exceeding 30,000, have been found to ingest plastic bags and ropes, with a mortality rate of 1% (124). When these petroleum byproducts are ingested, large stone-like masses known as polybezoars are formed, causing ruminal impaction. Analysis of the polybezoars from camel rumen revealed the presence of polyethylene and polypropylene (124, 125). Carcinogenic chemicals, including dioxins, phthalates, and polychlorinated biphenyls from plastic sources, have been reported in the milk, tissues, and rumen liquor of stray ruminants (126). These chemicals may be released, absorbed, and secreted in camels' milk or retained as chemical residues in meat (127), posing a potential cancer risk. The use and disposal of plastic bags and ropes are regulated in some Gulf countries but need to be addressed in others. Moreover, there is a need to raise awareness among camel owners about this issue.

### 3.8 Viral infections

Papillomatosis, caused by the papilloma virus, is widespread in humans and animals. Outbreaks of camel papillomatosis have been observed around the mouth and nostrils of young camels (18, 19, 21, 128, 129). The lesions were primarily seen in the fetlock area, skin of the neck, brisket, and cornea in adult camels (17, 22, 130), with spontaneous recovery reported. Papillomavirus types 1 (CdPV1) and 2 (CdPV2) were isolated from cauliflower-like lesions and rounded oval nodules, respectively, in young infected camels, and their genomes were characterized and grouped under Delta papillomavirus (21). Bovine papilloma viruses (BPV)-1 and BPV-2 bovine strains have been isolated from corneal papillomas in an adult dromedary (130). Different strains of papillomavirus can be implicated in various lesions in cattle; thus, BPV-2 and BPV-5 cause fibropapillomas, while BPV-3, BPV-1, and BPV-6 induce epithelial papillomas. BPV-4 has been reported in the upper alimentary tract, and BPV-5 and BPV-6 infect teats and udders, causing papillomas or fibropapillomas (131). Papillomavirus was diagnosed by PCR from mucocutaneous fibropapilloma lesions in adult camelids (132), exhibiting similar histopathological features to equine sarcoids caused by BPV types 1 and 2 (133, 134). There is a possibility of associations between some PV types and certain skin tumors in dromedaries.

Enzootic bovine leucosis (EBL) virus was suspected but could not be diagnosed in the sera of Arabian camels with high lymphoblastic leukemia (135–138). Hepatitis E virus is transmitted to humans through the consumption of contaminated food and can cause cancer. The virus has been isolated from camel serum, feces, and meat (139–141), with no reports of its implication in camel neoplasia, and it is not known if the camel contracts the disease with tumor formation or if it merely acts as a reservoir. Beta retrovirus infection has been reported in dromedaries, possibly constituting a species jump from sheep and goats (142). The virus caused tumor-like lesions around the nostrils and eyes, with involvement and damage to the ethmoid bone and possibly the nasal turbinate bones. Further studies are needed to elucidate whether the virus is related to the sheep retrovirus causing Jaagsiekte. With the advancement of recent virological methods, more PV viruses implicated in tumors in camels, especially skin tumors, are expected to be discovered.

### 3.9 Long exposure to sunlight

Long exposure to high ultraviolet radiation (UV) could damage DNA in the cutaneous epithelium, potentially causing neoplasia. Dromedary camels graze naturally in open areas and are exposed to prolonged sunlight. Cutaneous squamous cell carcinoma (SCCs) was more frequently seen in the abdominal wall of white-coated dromedary breeds (69.2%) compared to dark brown to black breeds (6). Furthermore, skin that is devoid of hair, such as the skin around the eyes, is particularly vulnerable (130, 143). UV radiation is a major cause of dermal hemangiomas and hemangiosarcoma in dogs (8, 144), cutaneous neoplasia in equines (8, 145), and squamous cell carcinoma in cattle, sheep, and goats (8, 146, 147), especially in exposed areas and in animals with white coats (8, 148). Additionally, the depletion of the ozone layer in the Earth's stratosphere, along with global warming, has a negative impact on human and animal health. An increasing incidence of basal cell carcinoma (BCC) and squamous cell carcinoma of human skin has been reported in areas of severe ozone depletion (149).

### 3.10 Inherited cancer genes

Most neoplastic diseases are not of familial origin, but the inheritance of cancer genes can increase the risk of specific types In general, some cancers are linked to mutations in genes inherited from parental lineage, such as BRCA1 and BRCA2, PTEN, WT1, WTX, CTNNB1, TP53, APC, and DICER1 (3, 5). Cancers caused by inherited defective genes have not been fully investigated in dromedary camels, and only a few tumor cases inherited through genetic mutations have been reported in different body systems. These include hamartomas caused by the PTEN gene and nephroblastomas primarily linked to the WT1 gene, and sometimes to WTX, CTNNB1, and TP53 genes (131). Primitive neuroectodermal tumors (131), with familial origins, have been reported in dromedaries. Arrhenoblastoma/Sertoli–Leydig cell tumors in dromedary camels (7, 10, 150) may be associated with mutations in the DICER1 gene, similar to those observed in children. Familial history of some ovarian teratomas in humans has also been linked to mutations in germ cell genes. We reported high ovarian and extra-gonadal teratomas with a relative frequency of 7.78%. Future genetic research on camel teratomas may suggest this species as an animal model for predicting malignant teratomas in humans and for recommendations regarding early surgery.

### 3.11 Age

Old age is associated with cancer risk in humans and animals. With advancing age, there is a buildup of damage within the cell, causing an accumulation of genetic changes. The DNA changes may increase the synthesis of some proteins that sustain cell growth, decrease the synthesis of other proteins that regulate cell growth, or halt the synthesis of proteins responsible for cell apoptosis. Old barren female dromedary camels were more likely to develop cancer compared to those of younger age (9, 130, 151).

## 4 Systemic tumors

The types of neoplasia in the different body systems of the dromedary are covered in Sections 5–13.

## 5 Digestive system

Reports of neoplasia in the digestive system of the Arabian camel are limited, except for the liver, which is a major site (Supplementary Table S2). Primary liver tumors in the dromedary can arise from liver cells, the biliary system, blood vessels, and lymphatics, or from combinations of these, whereas secondary neoplasia arises from hematogenous or lymphatic metastasis from different body sites or from invasion by neighboring organs (152). These include carcinomas, lymphomas, and fibromyxosarcomas.

### 5.1 Cholangiocarcinoma

Cholangiocarcinoma arises from intrahepatic or extrahepatic biliary cholangiocytes and is mostly observed in connection with inflammation and liver cirrhosis (153). Cholangiocarcinoma was reported concurrently with seminoma in an 18-year-old dromedary (154). Grossly, the liver was enlarged, discolored, and firm, with depressed areas and multiple small to medium lesions. Histopathology depicted pleomorphic tumor cholangiocytes with abnormal mitosis, arranged in irregular glandular or solitary structures of different sizes and shapes in the stroma, surrounded by fibrous septa. Multinucleated giant cells were occasional. Vascular invasion and capsular infiltration by tumor cells were evident. In addition, cholangiocarcinoma was described in the livers of two dromedaries (155). The hepatobiliary epithelium was well-differentiated within a connective tissue capsule. Again, cholangiocarcinoma was reported in an enlarged camel liver with many gray/white nodules of variable sizes (156). There was atypia of the biliary epithelium and tumor cell proliferation forming abundant ill-defined biliary ductules interspersed with fibrous tissue stroma. Hepatocellular hyperplasia was evident. Moreover, two cases of cholangiocarcinoma were reported in camel livers that were firm with thickened bile ducts and biliary cirrhosis (157). The tumor cells were well-differentiated and arranged in glandular or tubular structures of variable sizes and shapes. The neoplastic cells were separated by desmoplastic stroma. Again, Al-Hizab et al. (37) reported bile duct cholangiocarcinoma and hepatocellular carcinoma showing an array of pathological lesions due to very high levels of Aflatoxin B1 in the liver. Other cholangiocarcinoma cases were covered under the title “multiple primary tumors” in Section 5.6.

### 5.2 Squamous cell carcinoma

Squamous cell carcinoma was reported as multiple ulcerated cauliflower-like lobulated nodules attached among the papillae of the right cheek mucosa of a 12-year-old female (158) and was also reported as large masses at the ducal area of an adult male and at the gums of female dromedaries (6). Signs of dysmastia, dysphagia, drooling of blood, and loss of condition were observed. Grossly, the tumor was soft to firm with an irregular rough surface, grayish-pink in color, and may show multilobulation (6, 158). Histopathology depicted irregular tumor nests of stratified squamous epithelium invading the submucosa. The tumor cells displayed abundant keratinized cytoplasm and pleomorphic nuclei with high mitotic activity. Numerous keratin pearls and variable amounts of pale-staining fibrous connective tissue stroma were observed, but no evidence of metastasis (6, 158). Hepatocellular carcinoma was also reported in camel livers in abattoir surveys (37, 155). Grossly, the lesions appeared as multiple irregular nodules on the liver surface. In histopathology sections, the cells were predominantly arranged in the form of trabeculae, acini of different shapes, or as solid sheaths. The tumor cells were pleomorphic with large vesicular hyperchromatic nuclei and conspicuous nucleoli within a thin connective tissue stroma.

### 5.3 Adenocarcinoma

Omaso-abomasal adenocarcinoma was reported in a 15-year-old female dromedary with long-term clinical signs of anorexia, colic, cachexia, anemia, leukopenia, neutrophilia, and lymphocytopenia (159). Ultrasonography (US) showed a thickened, hyperechoic, vascularized, and corrugated omaso-abomasal wall. Laparotomy disclosed a very large hard mass with dense heterogeneous contents. Adhesions between the omasum and abomasum and the parietal surface of the peritoneum were observed. Grossly, the cut surface revealed thickened, congested, and ulcerated omaso-abomasal folds. Histopathology depicted acini of variable size lined by neoplastic epithelial cells with moderate atypia, separated by desmoplastic stroma and infiltrated with different inflammatory cells. The tumor was non-metastatic, invasive, and well-differentiated. Severe necrosis and suppuration were evident. It is worth noting that gastric adenocarcinoma was diagnosed in a 16-year-old Bactrian camel with extensive metastasis to different organs such as the liver, hepatic lymph nodes, lung, heart, portal vein, and aorta (160). Reports of omaso-abomasal adenocarcinoma are expected to increase because of the ingestion of plastic bags and ropes (124–126). In addition, one case of vaginal adenocarcinoma in a dromedary showed metastasis to the iliac lymph node, mesentery, and liver (161). Moreover, salivary fibro-adenocarcino-sarcoma with a pedunculated swelling at the lateral aspect of the left gum was diagnosed in an 8-year-old emaciated female dromedary (162).

### 5.4 Lymphomas

Lymphoma was reported in the liver of an adult male dromedary (151). Grossly, multiple whitish-gray nodules embedded in the parenchyma with focal infiltration were observed. Histopathology disclosed pleomorphic lymphocytic tumor cells arranged in follicles or sheets supported by fibrovascular stroma. The infiltrative lymphocytes contained eosinophilic granular cytoplasm with polygonal hyperchromatic anisokaryotic nuclei and mitotic figures. Moreover, a multicentric T-cell lymphoma was reported in a 7-year-old female dromedary camel with a high white blood cell count (163). Grossly, the tumor had metastasized to different organs, including the liver and small intestine. The liver displayed scattered pale median to large nodules, while the small intestines showed segmental thickening, and the lymph nodes were pale and enlarged. Histologically, all involved organs constituted tumor masses of rounded, monomorphic cells, depicting pale scanty cytoplasm and pleomorphic nuclei with conspicuous nucleoli and high mitotic figures. Metastatic lymphosarcoma was diagnosed in another dromedary. Grossly, the lesion showed multiple gray and white masses in the liver and other organs (157). Histologically, the tumor showed aggregates of pleomorphic neoplastic cells demarcated by thick fibrous stroma and inflammatory cells. Similarly, T-cell lymphoma was diagnosed in a 10-year-old male dromedary with a long history of illness (164). The gross lesions included multiple whitish, well-demarcated nodules of variable size in the liver and spleen, as well as enlarged abdominal and thoracic lymph nodes. Histopathology displayed infiltration of small to median-sized pleomorphic neoplastic lymphocytes with effaced organ structure. Furthermore, disseminated gamma-delta T-cell lymphoma was reported in a 12-year-old female dromedary, grossly showing a large mass on the right eye with metastasis to the liver and mesenteric lymph nodes, which showed multiple nodules (165). With the aid of flow cytometry, the camel's blood displayed a very high percentage of gamma-delta lymphoblasts. Immunohistochemistry (IHC) showed that the T-lymphoma cases were strongly positive for CD3 and negative for CD20, CD79a, MUM, and CD68 (151, 163, 164). Other myeloid lesions were observed in a complex benign tumor, myelolipoma with osseous metaplasia, diagnosed in the liver of a 10-year-old male dromedary (151). Grossly, the lesion was a grayish, circumscribed, hard small nodule. The tumor replaced the liver parenchyma and compressed adjacent hepatic tissue. Histopathology depicted a well-defined unencapsulated focal mass of mature and immature myeloid and erythroid hematopoietic cells, lymphocytes, macrophages, mature adipocytes, and immature woven bone arranged in sheets within a fibrovascular stroma. Hepatocellular degeneration, necrosis, and venous congestion were observed, along with amyloidosis, which was positive for Congo red stain.

### 5.5 Fibromyxosarcomas

Fibromyxosarcomas, a slow-growing malignant soft tissue tumor, were reported in two female dromedaries (6). Grossly, the lesions were numerous, small to medium, multilobular masses located on the gums, tongue, cheeks, and lips. Microscopically, the tumor displayed pleomorphic spindle-shaped and stellate-shaped fibroblasts, along with large pleomorphic dark-stained nuclei with a high mitotic index. The tumor stroma consisted of myxoid connective tissue and blood vessels. Multivacuolated cells filled with mucin were evident.

### 5.6 Multiple primary liver tumors

Cholangiocarcinoma-leiomyosarcoma was reported in the liver of a 15-year-old male dromedary (151). Grossly, the tumors showed multiple, firm, focally distributed whitish nodules, sometimes with centrally depressed areas. Histopathology depicted the occurrence of intrahepatic cholangiocarcinoma and leiomyosarcoma. The cholangiocarcinoma was unencapsulated, infiltrative, occupied most of the liver parenchyma, and circumscribed a few islets of degenerative cells. The neoplastic cells were arranged in ductal and acinar forms, sometimes with intraluminal papillae in bile ducts, and were separated by dense fibrous stroma. Focal areas of poorly differentiated pleomorphic cells with polymorphic nuclei were observed, which were positive for Cytokeratin 20. Histologically, the leiomyosarcoma displayed multifocal, circumscribed abundant interlacing bundles of smooth muscle cells separated by fibrovascular stroma. These bundles were separated by aggregates of fusiform tightly packed neoplastic cells with elongated nuclei and eosinophilic vacuolated cytoplasm arranged in a trabecular pattern. Anisokaryosis, bizarre nuclear atypia with multinucleoli, and frequent mitotic figures were observed. The leiomyosarcoma showed necrosis and infiltration by macrophages and lymphocytes. It was positive for alpha-smooth muscle actin (alpha-SMA) and stained yellow with Van Gieson's. Yet another case of hemangiosarcoma-cholangiocarcinoma-leiomyoma was reported in the liver of a 9-year-old male dromedary (151). Grossly, the liver displayed small, dark red or whitish well-circumscribed nodules. Histopathology depicted three types of tumors, including cholangiocarcinoma, hemangiosarcoma, and leiomyoma. The cholangiocarcinoma was a poorly circumscribed tumor formed in a large bile duct, with tumor cells poorly forming solid sheets and trabeculae within the lumen or infiltrating the wall to form ductules and acini supported by fibrous stroma. The cuboidal cholangiocytes showed eosinophilic granular/vacuolated cytoplasm and round/oval vesicular hyperchromatic nuclei with variable mitotic activity. Hemorrhage, mononuclear cell infiltration, necrosis, fibrosis, and/or cirrhosis were observed. The hemangiosarcoma showed unencapsulated infiltrative pleomorphic spindle cells forming irregular vascular cavities engorged with blood and replaced the liver parenchyma with a rare occurrence of solid sheets. The neoplastic cells exhibited anisokaryotic nuclei with one or two nucleoli and mitotic activity. Amyloidosis, atrophy, fatty degeneration, fibrin thrombi, and a few mononuclear cell infiltrations were observed. The tumor cells were positive for CD31. The vascular channels of hemangiosarcoma stained dark yellow with Van Gieson's stain. The leiomyoma was similar to that described above under the same title.

Salivary fibro-adenocarcino-sarcoma was diagnosed in an 8-year-old emaciated female dromedary (160). During clinical examination, a pedunculated swelling was observed at the left gum adjacent to the second premolar tooth, occupying the intermandibular space and extending to the lateral aspect of the ramus. The lesion was hard with a smooth surface, friable, and hemorrhagic. Grossly, the cut surface was gritty and presented cystic cavitation. Histopathology displayed anaplastic fibro-glandular neoplasm. The tumor cells were basophilic, granular, pleomorphic, and organized into tubules, acini, or solid sheets, seldom containing mucin, resembling the serous cells of the salivary gland, with nuclear atypia. The matrix stroma was myxomatous or fibrous and infiltrated by adenocarcinoma, whereas areas adjacent to the bone displayed malignant giant cells resembling anaplastic osteoclasts with multiple nuclei. The tumor capsule was infiltrated by lobular extensions of tumor cells. Mandibular bone invasion, osteolysis, cell necrosis, and sequestration were observed. The tumor possibly originated from the sublingual salivary gland.

### 5.7 Metastatic and invasive tumors

Tumors reported to metastasize to the dromedary liver included neuroectodermal tumor, probably from the spinal cord (166); multicentric fibro-myxoid peripheral nerve sheath tumor from peripheral nerves (167); disseminated gamma-delta T-cell lymphoma from the right eye (164); and metastatic vaginal adenocarcinoma from the vagina (161), along with other invasive tumors. These were covered under their proper body systems.

### 5.8 Leiomyoma

Leiomyoma, a rare smooth muscle tumor, originates from either the smooth muscles of the biliary system or the large veins of the liver and exhibits variability in size and number of lesions (168). Leiomyoma has been reported in the livers of two camels (113, 155) and in three adult males (151). Additionally, leiomyoma was diagnosed as part of a mixed Hemangiosarcoma-cholangiocarcinoma-leiomyoma tumor in the liver of a 9-year-old male dromedary (151). Moreover, leiomyoma was identified in a pedunculated mass attached to the abdominal wall, along with tumor masses in the wall of the left abdomen, kidney, and spleen of a 2-year-old male dromedary (169), with possible spread from smooth muscles due to expansion. Grossly, the lesions appeared as either small depressed areas (113), multiple whitish firm nodules, or large and small masses in different organs (151). Histopathology revealed multiple non-capsular, circumscribed lesions replacing the normal liver parenchyma. The tumor exhibited either a moderate or a large number of tightly packed interlacing bundles of spindle-shaped smooth muscle cells within a fibrovascular stroma. The tumor cells showed intense eosinophilic cytoplasm and cigar-shaped vesicular nuclei with rare mitotic activity. Bile duct hyperplasia and peribiliary fibrosis were evident. The tumor cells were positive for alpha-SMA and stained red by Masson trichrome against the blue staining of connective tissue, indicating proliferated smooth muscle cells rather than fibroblasts.

### 5.9 Fibroma

Fibroma was diagnosed in the digestive system of the dromedary (170). Additionally, a soft palate fibroma was described in an adult male dromedary with longstanding dysphagia, presenting as a large, hard, pink pedunculated ulcerated mass adherent to the soft palate (171). The tumor was also reported in a 5-year-old female camel exhibiting straining, rectal prolapse, and a large pendulous mass cranial to the anus (172). Diffuse proliferating fibrocytes with copious collagen deposits were observed under light microscopy (171). Moreover, a pedunculated irregular small fibrous epulis-like mass on the labial part of the upper gum was reported in a 2-year-old male dromedary (173). Grossly, the lesion was hard, lobulated, and had a rough surface. Histologically, the tumor was composed of fibroblasts and fibrocytes presented as whorls and bundles with collagen production. There were two keratin foci bordered by multinucleated giant cells, located deeply within the lesion. The squamous cells on the outer surface exhibited hyperplasia. The lesion resembles fibrous epulis in humans (173).

### 5.10 Multicentric schwannoma

Multicentric schwannoma was reported in a 4-year-old male Arabian camel (167). It constituted many small to large nodular masses in the serosa of the forestomach, large intestines, mesentery, liver, and spleen. Grossly, the lesions were discrete, rounded, white to gray, smooth, and bulged from the serosa with a homogenously cut surface. Histologically, the tumor cells were round or spindle-shaped, forming whorls and showing atypia. The discrete lesions were surrounded by a loose collagenous fiber stroma. In IHC, the tissue slides were positive for S100 and negative for CD34. The diagnosis was confirmed by electron microscopy.

### 5.11 Lipoma

Lipoma was reported in the livers of three slaughtered camels (113). The livers showed multiple raised white nodules. Light microscopy revealed non-capsulated masses between hepatocytes with multiple large vacuolated mature lipocytes. Leukocytic infiltration, fatty degeneration, and osteolipomatous metaplasia were observed. Similarly, an unusual myelolipoma with osseous metaplasia was reported in the liver of a 10-year-old male dromedary (151). In addition, lipoma was reported in the liver of an Arabian camel (10) and in the left ischiorectal fossa underneath the skin of a 7-year-old male dromedary (174). The leptocytes were positive for Congo red stain.

### 5.12 Cavernous hemangioma

Cavernous hemangioma was reported in the livers of two camels (157, 175). The tumor was a small spongy dark red rounded mass engorged with blood. Histopathology revealed irregular blood cavities lined by a single layer of endothelium. The adjacent liver tissue underwent hepatocellular necrosis and pressure atrophy. Similarly, El Miniawy et al. (155) reported two cases, and Rezaie et al. (113) reported one case of cavernous hemangioma in dromedary livers. Grossly, the liver showed brown raised lesions separated by a fibrous connective tissue capsule (113).

### 5.13 Summary of tumors relative frequencies in the digestive system

The relative frequency of tumors reported in the digestive system of dromedary camels was 6.31% (Supplementary Figure S1). Carcinomas were the main tumors in the camel digestive system, with a relative frequency of 2.20%, primarily due to liver metastasis. Carcinomas have previously been reported to be high in the digestive systems of different animal species (8, 10), raising global public health concerns in humans (153). Recently, multiple primary liver tumors were reported (151). The co-occurrence of these multiple tumors is abnormal and may possibly indicate different interactions of tumor risk factors, as the liver is the organ responsible for detoxifying xenobiotics (63, 65). The relative frequencies of other malignant tumors in the camel digestive system were as follows: lymphomas 0.733%; sarcoma 1.613%; and primitive ectodermal tumors 0.147%. Pancreatic and colorectal malignancies, mostly observed in older individuals, were not reported in this species, although most of the abattoir cases were old barren females. The relative frequencies of benign tumors were not high (Supplementary Figure S3) in the digestive system of the camel, i.e., fibroma 0.587%; hemangioma 0.88%; lipoma 0.733%; and leiomyoma 0.88%.

## 6 Respiratory system

The neoplasia of the respiratory tract in the dromedary (Supplementary Table S3) was mostly malignant.

### 6.1 Carcinomas

Bronchioloalveolar adenocarcinoma, an epithelial malignant neoplasia, was reported in a 12-year-old emaciated female camel (176). The neoplasia was primary and likely originated from the alveolar and/or bronchiolar epithelial layers. Grossly, the tumor showed multiple, pale yellow-gray spherical masses of variable size, with fragile consistency and a smooth cut surface, either projecting from or embedded in both lungs. The bronchial lymph node was enlarged, irregular, and displayed a hemorrhagic cut surface. Histopathological findings depicted alveolar walls lined with anaplastic cuboidal cells and intra-alveolar spaces obliterated by pleomorphic anaplastic cells with large hyperchromatic nuclei. Around the alveoli, the cells were arranged in solid sheets, clumps, or acini within a thin stroma. Degenerative and inflammatory lesions were observed in lung tissue. The texture of lymph nodes was replaced by pleomorphic neoplastic cells similar to those in the lungs. The neoplastic cells showed moderate mitotic figures. Metastasis was noted in lymphatic vessels. Another case of bronchoalveolar adenocarcinoma was also reported in the lungs of a dromedary (177). Histopathology of the neoplasm displayed well-differentiated tall pleomorphic cells with basal nuclei, arranged in sheets or acini with mucin secretion. Moreover, pulmonary papillary carcinoma was reported in four 15-year-old emaciated female dromedaries (115). Grossly, the lesion constituted multiple, variable-sized, pale yellow, well-defined soft masses distributed throughout the lungs with a hemorrhagic cut surface. Histopathology revealed primary papillary projections with secondary and tertiary branches completely obliterating the lung tissue, lined with overlapping clumps of small cuboidal/columnar tumor cells in a fibrovascular core. Psammoma bodies were identified. These tumor cells showed nuclear atypia, with vesicular empty glass nuclei and prominent dark nucleoli. The stroma showed mononuclear cell infiltration, hemorrhage, necrosis, and edema. A poorly differentiated anaplastic mammary carcinoma metastasized to the lungs and bronchial lymph nodes of a 15-year-old female dromedary (178). In histopathology, the lung parenchyma, microvasculature, and lymph node showed anaplastic epithelial cells similar to those in the mammary gland. There was ventral atelectasis in the lungs, erythematous pleurae with red-brown pleural effusions of reactive mesothelial cells, and a thickened pericardium.

### 6.2 Sarcomas

The sarcomas reported in the dromedary lungs were mostly metastatic. Osteosarcoma metastasizing from the left ulnar to the lungs was reported in a 7-year-old female dromedary (179). Clinically, the animal manifested tachycardia, tachypnea, and mouth breathing. Grossly, the lungs were filled with a large quantity of red turbid pleural effusion. Many nodules of variable sizes, pale to white, with necrotic centers were identified along with a ventral fibrous adhesion. Extensive hemorrhage and necrosis were observed within the centers of the nodules. The nodules were non-encapsulated and histologically demonstrated densely populated, haphazardly arranged pleomorphic spindle-shaped neoplastic cells intermingled with a few bundles of collagen and vasculature. Osteoid was occasionally observed. The neoplastic cells were hyperchromatic with a moderate number of mitotic figures. The thoracic lymph nodes were mottled, enlarged, edematous, hemorrhagic, but did not disclose any neoplastic cells. In another case, chondrosarcoma metastasizing to the lungs from the left carpal joint was reported in a 4-year-10-month-old female dromedary with long-term lameness (180). The lung lesions consisted of gray, firm, multiple nodules. Histopathology displayed poorly demarcated nests of non-encapsulated, invasive chondrocytes in a cartilage matrix with cellular atypia similar to the primary neoplasia. The cytoplasm of the neoplastic cells showed diffuse brown vimentin and S-100 staining by IHC. A disseminated sarcoma was reported in an adult female dromedary which showed a deep wound above the right tarsus (181). Aside from the wound, multiple abscesses of different sizes were grossly observed to be disseminated in the lungs, along with one in the thoracic wall and another in the pericardial sac. The histological picture disclosed multifocal areas of massive accumulation of neoplastic cells in the lungs and the primary site (wound). On another occasion, a poorly differentiated rhabdomyosarcoma was reported in a 9-year-old female dromedary (182). Clinically, the tumor presented as a pedunculated, hard, cauliflower-like nodular growth attached to the ventrolateral part of the nasal mucous membranes and obstructing the right nostril.

### 6.3 Lymphomas and leukemia

Pulmonary lymphosarcoma was reported in a 16-year-old female dromedary (183) which collapsed and died. Necropsy showed very solid lungs, especially on the left side. Histopathology revealed diffuse lymphosarcoma of small lymphocyte-like cells in the lung tissue without involvement of lymph nodes. Edema, emphysema, and pneumonia were observed in the lung tissue. In addition, T-cell lymphoma was reported in ten dromedary camels that died within 6 months of diagnosis (137). All affected camels developed leukocytosis with dominance of lymphoblastic cells. Histopathology showed extensive infiltration of lymphoid cells in the lungs, mediastinal lymph node, and spleen, which were CD3 positive by IHC. The sera and tissues of affected camels were negative for bovine leukemia virus.

### 6.4 Benign mesenchymal tumors

A multiple leiomyomatous hamartoma, an extremely rare condition, was diagnosed in the lungs of a female dromedary by EM (184). Generally, the tumor contains two or more mesenchymal elements. It was therefore assumed to originate from contractile interstitial cells along with type II pneumocytes. On another occasion, pulmonary leiomyoma was reported in two male dromedaries, aged 8 and 10 years. The tumor is rare in the lungs (184). The camels were asymptomatic, and the neoplasia was discovered only during post-mortem examinations. Grossly, the tumor appeared as neoplastic masses in the visceral pleura of one camel and in lung tissue in the other (185). Light microscopy displayed proliferating neoplastic cells closely resembling smooth muscle in one case, while the other showed well-differentiated spindle-shaped smooth muscle cells in the form of interlacing bundles within scant fibrous stroma, demarcated by a connective tissue capsule. Other lung lesions included atelectasis and emphysema in some areas of the alveoli. The leiomyoma was suggested to originate from the smooth muscle layer of the pleural blood vessels. Another tumor, lobular capillary hemangioma (pyogranuloma), appearing as an overgrowth in the retropharyngeal region, was reported in a dromedary by ultrasound (US), and the diagnosis was confirmed by histopathology (186).

### 6.5 Summary of tumors relative frequencies in the respiratory system

The relative frequency of tumors in the respiratory system of camels accounted for 3.81% (Supplementary Figure S1). The tumor types and their relative frequencies among our collected data were carcinomas (1.02%); lymphomas (1.47%); sarcomas (0.73%); leiomyoma (0.44%); and hemangiomas (0.147%). The relative frequency of malignant lung tumors in dromedary camels is not as high as that generally reported for humans. Smoking and air pollution in crowded towns and industrial areas are the principal risk factors for lung cancers and deaths worldwide (15, 107, 119, 187). Tumor metastasis from various body organs to the lungs is common in camels and similar to other species (8, 82).

## 7 Lymphatic and hematopoietic systems

The neoplasia of the lymphatic and hematopoietic systems (Supplementary Table S4) includes lymphoma and leukemia, which are malignant and originate from the lymphatic system and bone marrow/lymph nodes, respectively, whereas hemangiomas are benign tumors of poorly understood etiology (188).

### 7.1 Lymphoma

Lymphoma is a group of malignant tumors of the lymphatic system, primarily seen in adults (8, 187). The primary extranodal lymphomas arise outside the lymph nodes. Epstein-Barr virus, HIV, and autoimmune diseases are commonly implicated in human lymphomas (189). Hodgkin lymphoma (characterized by Reed-Sternberg cells) and non-Hodgkin lymphoma (NHL; the common type) are the main categories of these tumors. B- and T-cell lymphomas are types of NHL, arising from B and T lymphocytes, respectively, with B-cell types constituting 90% of cases in humans. However, T-cell lymphomas were the only types reported and are relatively common in dromedaries, often with a fatal outcome. Lymphoma has been identified in different body parts of the camel, and the lesions are detailed under the specific system where they occur. Consequently, pulmonary T-cell lymphoma and lymphosarcoma (137, 183) are described under the respiratory system; liver lymphosarcoma (157) under the digestive system; multicentric T-cell lymphoma (163) is included in the digestive and urinary systems; disseminated gamma-delta T-cell lymphoma (164) was included under the digestive system as a metastatic condition from the eye, and superficial skin lymphosarcoma nodules were reported under the skin and appendages of the neck (186). In addition, lymphatic lymphomas were reported (190, 191) in the dromedary, but the involved organs could not be specified. On the other hand, B lymphoma was not reported in the dromedary as in other domestic species, and its sera were negative for retroviruses causing enzootic bovine leucosis, which infects B cells (137). Similarly, the New World camelids diagnosed with lymphoma were negative for Epstein–Barr virus and ovine pulmonary adenomatosis antibodies (192, 193). The risk factors for non-Hodgkin's lymphoma are not known; however, autoimmune diseases, low immunity, chronic bacterial and viral infections, chemicals, and old age may be among the causes (194).

### 7.2 Leukemia

Leukemia, mostly diagnosed in children, starts in the bone marrow and can be acute or chronic, where mutated lymphocytes outnumber other white blood cells (195). A high count of immature lymphocytes in peripheral blood is mostly indicative of leukemia. These tumors are classified into fast-growing acute myeloid leukemia or slow-growing chronic myeloid leukemia (CML). When leukemias originate in the bone marrow and later spread to lymphoid tissue, they are classified as acute lymphocytic leukemia (ALL) or chronic lymphocytic leukemia (CLL) (196, 197).

Chronic aggressive myeloid leukemia (CML) was diagnosed in a 10-year-old female dromedary (198). The clinical signs included emaciation, dullness, anorexia, and anemia without enlargement of lymph nodes. Microscopically, the total white blood cell count was very high, with 97% being lymphoblastic and lymphocytic cells. The lymphoblasts were large and pleomorphic, with strongly basophilic cytoplasm, pleomorphic nuclei, prominent nucleoli, and frequent mitotic figures. Remarkable cellular and nuclear atypia, including cytoplasmic blebs, indented or double nuclei, and increased nuclear:cytoplasmic ratio, were observed. In another case, lymphocytic leukemia (CLL) characterized clinically by cachexia and enlarged lymph nodes was diagnosed in 10 dromedaries over 8 years of age within a period of 6 years (137). The camels showed leukocytosis in blood cell count, with 90% of these counts being lymphoblasts. All the affected camels died within 6 months of diagnosis. The necropsy revealed enlarged lymph nodes, along with other secondary lesions. Histopathology depicted extensive infiltration of neoplastic lymphoid cells in the lungs, lymph nodes, and spleen. Offspring of two infected females were born with normal WBC counts. Two healthy camels injected with heparinized blood from the leukemic animals showed normal blood counts after follow-up for 1 year. Lymphocytic leukemia was again reported in dromedary camels (199). The neoplasm was exclusively of lymphoblasts (870 10^9^/L; 100%). Moreover, acute lymphoblastic leukemia (ALL) was reported in 10 (12) and in two camels with very high leukocyte counts that died 16 days after diagnosis (138). The etiology of lymphocytic leukemia has not yet been revealed, and the sera of camels with lymphoblastic leukemia, tested in India (135) and UAE (136–138), were negative for enzootic bovine leucosis (EBL) virus antibodies.

### 7.3 Hemangiomas

Hemangiomas, a benign vascular neoplasia, can be found anywhere in the body but are common in the skin of the face and neck. These are categorized as either cavernous or capillary hemangiomas (200). The cavernous type is characterized by thin-walled, widely packed larger lumens of clustered blood vessels with thick collagenous trabeculae and occurs in adults. The capillary hemangioma displays many clusters of tightly packed small capillaries with endothelial linings and is mostly present at birth. Cavernous hemangioma was reported in the liver of a dromedary and detailed under the digestive system (155, 157, 175). Additionally, cavernous hemangioma was reported in the left ventral superficial cervical lymph node of an emaciated 10-year-old she-camel (201). Clinically, the lymph node was a very large hard mass protruding from the skin cranial to the chest pad. The cut surface of the lymph node was dark red in color. In histopathology, the tumor lesion comprised small and medium non-capsulated thick or thin-walled vascular clusters filled with blood and separated by conspicuous stroma, which was demarcated by many crowded proliferating capillaries. Yet in another case, lobular capillary hemangioma (pyogranuloma) was reported in the retropharyngeal region of a dromedary, detected using US and confirmed by histopathology (186).

### 7.4 Mastocytoma

Mastocytoma, a benign or malignant round-cell tumor of mast cells, has been reported in many species but is most commonly seen in dogs (9). The neoplastic mast cells can accumulate focally or diffusely in various organs. The tumor produces histamine, heparin, and various other proteins and chemicals causing allergies and attracting WBCs. Mastocytoma was reported in the dromedary without any details in the report (191).

### 7.5 Summary of tumor relative frequencies in lymphatic and hematopoietic system

The lymphatic and hematopoietic system represented a relative frequency of 6.891% (Supplementary Figure S1). The tumor types and their relative frequencies in the system were lymphomas (2.49%), leukemia (3.37%), hemangioma (0.88%), and mast cell tumors (0.15%). Lymphomas and leukemia were common occurrences in humans and domestic animals (8, 50, 189, 193, 195, 197). All reported cases of lymphomas in camels were NHL T-cell types. NHL B-cell lymphoma, more common and less aggressive in humans and animals, and Hodgkin lymphomas have not yet been diagnosed in the dromedary. More research is required to elucidate the risk factors behind lymphomas and leukemia in the dromedary camel, including oncogenic viruses and pesticides (50). Unlike the case in small animals (8), mast cell tumors had a very low relative frequency in camels.

## 8 Musculoskeletal system

The tumors of the musculoskeletal system reported in the dromedary included squamous cell carcinoma, osteosarcoma, chondrosarcoma, osteomas, myxoma, and fibroma (Supplementary Table S5).

### 8.1 Squamous cell carcinoma

Intraosseous squamous cell carcinoma was reported in a 6.5-year-old female dromedary with a 2-month history of a very large firm mass at the right caudal maxilla, with focal skin ulceration, third eyelid protrusion, and difficulty chewing (164). Radiography showed an expansile radiopaque mass in the maxilla. Histopathology displayed polygonal epithelial cells with eosinophilic, sometimes vacuolated cytoplasm, central nuclei with few mitotic figures, and connections via intercellular bridges. The neoplastic cells were arranged in cords, islands, and plexiform ribbons within scant collagenous stroma. Central keratinization was observed. The tumor lacked odontogenic epithelium and central stellate reticulum. IHC depicted strongly positive neoplastic epithelium for pancytokeratin and vimentin for tumor stroma. Similarly, squamous cell carcinoma was reported in a 9.5-year-old female dromedary with a rapidly growing large mass in the middle of the left maxilla after 5 months of excision (164). The epithelial arrangements, tumor stroma, and tumor immunostaining were similar to those of intraosseous squamous cell carcinoma. However, central cystic spaces suggested odontogenic epithelium with numerous small islands of woven bone but without peripheral palisading. Again, squamous cell carcinoma, diagnosed by US and histopathology, was reported in a dromedary showing superficial swelling at the maxilla (186). Moreover, a well-differentiated squamous cell carcinoma was reported in a 7-year-old male dromedary located at the skin of the right hock joint and invading the tarsal and metatarsal area (202). The tumor was described under skin and appendages (Section 12.1).

### 8.2 Osteosarcomas

Osteosarcoma was reported in a 7-year-old female dromedary camel presented to the clinic with severe lameness, a swollen left elbow joint, dullness, tachycardia, and tachypnea (179). Anemia, leukocytosis, low ionized calcium, and severe metabolic acidosis were reported. A pathological fracture of the proximal left ulnar was diagnosed by radiography. Necropsy showed a granular fractured surface, eroded articular surfaces, cavitated areas, friable bones, and necrotic tissue. The tumor displayed metastasis, causing small nodular masses in the lungs. Bone specimens from the lesion for histopathology revealed spindle neoplastic cells similar to those observed in the lung masses. A meager amount of osteoid was occasionally observed in sections, confirming the diagnosis of osteosarcoma along with features of pathological fracture, i.e., granular surface of fractured bone, bony lysis, and hemorrhage. Similarly, osteosarcoma was diagnosed ventrally in the mandible of a dromedary camel by US and histopathology (186). In addition, salivary fibro-adenocarcino-sarcoma was diagnosed in an 8-year-old emaciated female dromedary (160). Although the tumor had osteolytic activity and local mandibular bone invasion, no metastasis was observed. The tumor lesion was described under the digestive system (Section 5.3).

### 8.3 Osteomas

Vertebral osteoma, uncommon in domestic animals, was reported in a 4-year-old female dromedary in a zoo (203). The animal manifested progressive ataxia, hind limb weakness, and recumbency. Laboratory investigations revealed anemia and high ALP enzyme levels, which originate only from the liver and bone and reflect osteoblastic activity (65). Necropsy revealed that the 10th thoracic vertebral body presented expansive hard tumor growth attached at the right side, compressing the vertebral canal. The periosteum presented fibrous and osteogenic layers, and the cortical margins depicted irregular areas of compact bone with incomplete mineralization and deep bony trabeculae. Furthermore, maxillary osteoma was reported in an old female dromedary (9). The tumor presented as a very large firm swelling on the right maxilla extending to the orbit. Surgical excision revealed a very large, rounded mass with a rough surface, and diagnosis was achieved by histopathology.

### 8.4 Chondrosarcoma

Primary chondrosarcoma was reported in a 4-year and 10-month-old female dromedary (180). The camel showed progressive long-term lameness and swelling of the left carpal joint. Radiography revealed multifocal radiolucent marinated lesions involving the carpus and proximal metacarpus. Necropsy displayed a thickened joint capsule, a large whitish pale multi-lobulated synovial membrane, and two smaller masses at the carpal joint. These two masses infiltrated and multifocally eroded the articular surface of the carpal/metacarpal bones with loss of osteoid. The tumor metastasized to the lungs, presenting multiple gray firm small nodules. Histopathology showed that the tumor was unencapsulated and poorly delineated. It presented invasive islets of neoplastic chondrocytes in a cartilage matrix separated by dense fibrous connective tissue and infiltrated by lymphocytes, plasma cells, and macrophages. The malignant chondrocytes showed atypia, pleomorphic cells with abundant eosinophilic cytoplasm, rounded eccentric nuclei/multiple nuclei with mitotic figures. Carpal and metacarpal bone lysis with loss of osteoid was observed. IHC displayed diffuse positive cytoplasmic staining for vimentin and S-100. Chondrosarcoma was also reported in a 14-year-old female with a wound that recurred two times after surgery. The wound was of median size on the solar aspect of the left hind foot (204). A pinkish multiple papillae-like lesion was removed, and the tumor was diagnosed by histopathology.

### 8.5 Rhabdomyosarcoma

A pleomorphic rhabdomyosarcoma with poorly differentiated primitive mesenchymal cells was reported in a 9-year-old female dromedary (182). The lesion was attached to the ventrolateral part of the nasal mucous membranes and obstructed the right nostril. It was circumscribed with a rough mottled and soft gray cut surface. Histopathology revealed that the tumor was densely populated with clusters of small to medium-sized pleomorphic cells that were haphazardly arranged. These cells were intermingled with large polygonal rhabdomyoblasts embedded in a richly vascularized scant stroma. The tumor cells displayed pleomorphic nuclei with frequent mitotic figures. The rhabdomyoblasts were scarce, striated, and strongly positive for desmin by IHC. Langhans giant cells were frequent.

### 8.6 Metastatic tumors

A neuroectodermal tumor, probably originating from the spinal cord, metastasized to the third lumbar vertebra and liver, as diagnosed and described under the nervous system (166).

### 8.7 Ameloblastoma

Conventional ameloblastoma, a rare benign, locally invasive, solid, multicystic intraosseous odontogenic epithelial tumor, was diagnosed in a 5-year-old female dromedary with a history of a large, firm, ulcerated mass at the right caudal maxilla, along with hypersalivation and oral mucosa ulceration (205). A large multicystic mass in the maxillary bone, sinuses, and third molar tooth was revealed by radiography. Histopathology displayed an infiltrative, unencapsulated tumor in the submucosa. The tumor was composed of large lobules and broad interlacing trabeculae separated by dense fibrovascular stroma. The tumor was covered by odontogenic epithelium consisting of 1–2 layers of palisading columnar cells, with antibasilar nuclei and basally vacuolated cytoplasm. The centers of the neoplastic lobules and trabeculae were composed of less cellular keratinized stellate reticulum; however, dense areas of interlacing bundles or whorls with spindle cells were observed. Cysts with proteinaceous material were occasionally noted within the lobules. The fibrous stroma was less cellular, with collagenous bundles and focal areas of woven bone and osteoid. The submucosa was infiltrated by lymphocytes and plasma cells. The IHC showed strong reactivity of the odontogenic epithelium and stellate reticulum for pancytokeratin and was mostly negative for vimentin, except in areas neighboring the odontogenic epithelium. However, the stromal cells were vimentin positive and pancytokeratin negative.

### 8.8 Odontogenic fibroma

A central odontogenic fibroma, a rare fibrous tissue tumor of the musculoskeletal system, was reported in an 8-year-old female dromedary with a history of a very large mass at the right maxilla and a missing second molar tooth in the area (205). Radiography revealed a large radiopaque structure with focal osteolysis at the right maxilla. In histopathology, the neoplasm presented scarce cellularity, primarily consisting of fibrous connective tissue, with lobular woven bone occupying 70% of the tumor tissue and scattered islands of odontogenic epithelium to confirm the diagnosis of odontogenic fibroma. A dense collagenous stroma and a few uniformly spindle-shaped cells with scant cytoplasm and oval-round nuclei were observed. In IHC, the odontogenic epithelium, which showed peripheral palisading and antibasilar nuclei, was strongly positive for pancytokeratin. However, the spindle cells of the connective tissue were strongly positive for vimentin.

### 8.9 Intramuscular myxoma

Benign intramuscular myxoma, a mesodermal tumor, was reported in the anterior aspect of the hock joint of an 8-year-old female dromedary (206). The tumor was very large and covered with dark, hairless skin. Grossly, the tumor appeared as a whitish, solid mass similar to connective tissue, infiltrated and encapsulated within striated muscles, and oozed blood. Histologically, the tumor was composed of stellate to spindle-shaped cells with scant cytoplasm and small hyperchromatic nuclei scattered in a dense myxoid matrix. The tumor exhibited low cellularity with focal areas of hypercellularity, blood congestion, and eosinophilic infiltration.

### 8.10 Summary of tumors relative frequencies in musculoskeletal system

The relative frequency of tumors in the musculoskeletal system of camels was low, accounting for 2.49% (Supplementary Figure S1). The frequencies of individual musculoskeletal tumors included a few malignant types: carcinomas (0.73%), sarcomas (0.88%), and primitive ectodermal tumors (0.15%). The relative frequencies of benign tumors were also low, namely, osteomas (0.29%), fibromas (0.15%), myxomas (0.15%), and ameloblastomas (0.15%); however, chondromas were not reported. Odontogenic tumors, a diverse group of malignant and benign tumors arising from components of tooth development, were diagnosed in some oral cases, including squamous cell carcinoma, ameloblastoma, and odontogenic fibroma, with a relative frequency of 0.44%. These tumors are common in humans and dogs and directly affect feeding and general health.

## 9 Reproductive system

The main tumors reported in the reproductive system of the dromedary (Supplementary Tables S6a, b) included teratomas, adenomas, sex cord-stromal tumors, and fibromas in the ovaries (11, 207, 208); adenocarcinomas and cavernous hemangiomas in the uterus (150, 161, 208); adenocarcinomas in the vagina and cervix (23, 161); and adenocarcinomas, adenomas, and fibromas in the mammary gland (116, 178, 209, 210). Male tumors were rarely reported (154, 211, 212).

### 9.1 Testicular and ovarian tumors

The tumors of the ovaries and testicles were variable and included germ cell tumors (95%) and sex cord-stromal tumors (5%), which occur in both sexes. Ovarian tumors are of special importance as they have a direct impact on reproduction (213, 214). Ovarian neoplasia accounts for about 0.06%−2.0% in dromedaries; however, higher incidences of 6.8% (117) and 8.30% (11) have been reported in certain countries (Table 1). Testicular tumors and associated structures in dromedary bulls are comparatively rare (12, 215). However, anabolic steroids and/or testosterone therapy to augment bull libido have been reported to reduce the testicular size sperm count and motility (213, 215).

#### 9.1.1 Germinal cell tumors

The germ cell tumors, which originate from germ cells producing sperm or eggs, include seminoma and non-seminomatous germ cell tumors (NSGCT). The germ cell tumors reported in dromedary camels include seminoma in males (154, 211, 212), dysgerminoma (an alternate name for seminoma) (207), and teratomas in females (208, 216–218). The NSGCT consists of several types and can secrete hormones (217).

##### 9.1.1.1 Seminoma

Seminoma is a malignant, solid germ cell neoplasia that arises from the germinal epithelium of the seminiferous tubules and is commonly confined to the testicles. It presents three types in humans (219) and secretes testosterone and chorionic gonadotropin. The tumor was diagnosed in male dromedary camels, with an incidence of 0.3% (211). Seminoma of the right testicle and intrahepatic cholangiocarcinoma in the liver were reported simultaneously in an 18-year-old dromedary (154). Additionally, a well-differentiated seminoma was diagnosed in the right testicle of a healthy 10-year-old dromedary bull with a breeding history of exclusive females (212). Clinically, the lesions were confined to the testes. The right testicles were hard, freely movable, and enlarged (154, 212). The US of the right testicle revealed diffuse heterogeneous parenchyma with echogenic lobules (212). The gross examination and histopathology showed some differences. The testicular gross lesions showed either a lobulated multicolored cut surface (154) or enlargement with a loss of texture and numerous soft bulging nodules with an impalpable epididymis (212). The histopathology depicted pleomorphic rounded or oval tumor cells with vesicular or hyperchromatic single or multinuclei and prominent nucleoli (154). The tumor cells were distributed in the stroma, indicating diffuse type seminoma. However, Ali et al. (212) reported aggregative, closely packed, sharp-bordered, polyhedral pleomorphic cells with round or oval nuclei and prominent nucleoli, closely resembling germinal epithelium (spermatogonia). Few mitotic figures were evident. Fibrous trabeculae, focal hemorrhage, necrosis, and mononuclear cell infiltration were observed in the stroma.

#### 9.1.2 Female germ cell tumors

##### 9.1.2.1 Dysgerminoma

Dysgerminoma, a malignant germ cell ovarian tumor, has no or very rare endocrine manifestations. Dysgerminoma was reported in the left ovary of a dromedary over 16 years old (207). The tumor was pale gray and soft, with two small lobes protruding from the ovarian surface. Microscopy revealed cell masses arranged in compartments with scant stroma, separated from other tissue by a thin layer of connective tissue. Tumor cells were uniformly large, mitotically active, polyhedral, with scant cytoplasm and hyperchromatic nuclei containing one or more nucleoli. Focal hemorrhage, cellular necrosis, and accumulation of lymphocytes and histiocytes were evident. The tumor cells showed lymphatic and adjacent tissue invasion. A hyperplastic rete ovarii was located adjacent to the tumor base.

##### 9.1.2.2 Ovarian teratomas

Teratomas are benign germ cell tumors that can occur in various body parts, but are mainly found in the ovaries, testicles, and sarcoccygeal region. Ovarian teratomas are slow-growing and can be single or multiple, unilateral or bilateral, smooth, thick-walled, gray-colored hollow masses of variable size protruding from the ovarian surface or embedded in the ovarian medulla (12, 216, 220). The ovarian tumor can be diagnosed through rectal palpation, US, exploratory laparotomy, and histopathology, and has no effect on ovulation and conception when less than 30 mm in diameter (221). Ovarian teratomas are common in the dromedary and have been reported in ten (216), five (222), six (208), two (150), and one case each (220, 223–225) and on other occasions (226–228). Grossly, the teratoma comprises a thick fibrous capsule enclosing a cavity filled with a variety of heterogeneous tissues and materials, such as cartilage, bone, hair, sebaceous secretions, and brownish greasy keratinized materials (150, 223). In histopathology, the cavity is covered with stratified keratinized squamous epithelium, lined with masses of hair follicles, sweat and sebaceous glands, and enclosed by thick fibrous connective tissue and bundles of smooth muscle (150, 221, 223, 228).

#### 9.1.3 Sex cord-stromal tumors

Sex cord-stromal tumors occur infrequently (5%) and have been reported in both sexes. They include granulosa cell tumors (GCTs), Leydig cell tumors, Sertoli cell tumors, and mixed and unclassified testicular sex cord–stromal tumors (229), among others. Sex cord-stromal tumors secrete hormones. Thecomas and granulosa cell tumors produce estrogen, whereas Sertoli and steroid cell tumors produce testosterone (230). Sertoli–Leydig cell tumors in humans may be associated with thyroid disorders and DICER1 gene mutations and show positive results for blood inhibin and alpha-fetoprotein (AFP) (231).

##### 9.1.3.1 Granulosa cell tumors

Granulosa cell tumor is a non-epithelial, usually malignant, slow-growing sex cord-stromal tumor derived from ovarian granulosa cells that secretes estrogens. The tumor was reported in a 14-year-old multiparous dromedary female with a previous history of exhibiting male behavior (232). Moreover, an adult granulosa type was reported in a 7-year-old female (11) and in a non-pregnant adult dromedary female (233). The tumor presented on US as a honeycombed echogenic ovary mixed with focal hypoechoic lobular masses (232). Grossly, the tumors displayed different forms, i.e., as an enlarged grayish-white firm multinodular mass (232), a nodule attached to an enlarged ovary by a long stalk, or as variable-sized masses (233). The tumors showed a polycystic cut surface containing hemorrhagic fluid. Light microscopy revealed obliteration of the normal ovarian tissue architecture by proliferating nests or solid sheets of a dense number of mature polyhedral granulosa cells. These cells exhibited indistinct boundaries, scant cytoplasm, and round to oval nuclei with active mitosis or pale nuclei with nuclear grooves. Occasionally, Call–Exner bodies were seen intermingling with the granulosa cells. The tumor cells were supported by a light fibrovascular stroma and enclosed by a thick fibrous capsule invaded by neoplastic granulosa cells. Multifocal sarcomatous changes, constituting pleomorphic round and spindle-shaped cells with bizarre nuclei and mitotic figures, were observed (11). Congestion and hemorrhage were occasional (11, 232). The tumor cells were vimentin positive by IHC (11).

##### 9.1.3.2 Thecoma/fibrothecoma

Thecoma and fibrothecoma are benign, uncommon sex cord-stromal tumors that secrete estrogen. The tumor was reported in the ovaries of two dromedary females, aged 5 and 11 years (11). The left ovary was significantly enlarged and contained small firm gray-white nodules. Microscopy revealed effacement of the ovarian cortex by non-encapsulated, densely packed tumor cells. The tumor exhibited oval and spindle-shaped streaming thecal cells with unclear boundaries, occasionally displaying pale vacuolated cytoplasm (lipid-rich) and round to oval nuclei. The cells clustered in diffuse or variable nodular patterns and were supported by bundles of fibrovascular stroma composed of collagen fibers. An occasional fibrothecoma, a mixture of fibroma and thecoma, was observed in the tumor.

##### 9.1.3.3 Granulosa-theca

Granulosa-theca cell tumor, a benign mixed sex cord-stromal tumor, was reported in the ovaries of ten 5- to 11-year-old females (11). The ovaries were enlarged with firm gray and white nodules of variable size, along with several yellow cysts. Histopathology depicted an unencapsulated expansive tumor of high cellularity that obliterated the ovarian tissue. The granulosa cells, with unclear boundaries and nuclear grooves, coexisted with streaming spindle theca tumor cells that had clear cell borders, scant eosinophilic cytoplasm, and oval to round nuclei, forming different tumor patterns. These patterns included macrofollicular, trabecular, and diffuse patterns, with the latter two showing Call–Exner bodies along with various uncommon forms. The tubular-like pattern was strongly and diffusely positive for vimentin and focally positive for inhibin, whereas the Sertoli-like pattern showed positive cytoplasm for vimentin and Melan A and was negative for inhibin.

##### 9.1.3.4 Leydig cell tumors

Interstitial Leydig cell tumor, a rare benign sex cord-stromal tumor of the ovaries and testicles, arises from Leydig cells and secretes testosterone. Interstitial (Leydig) cell tumor was reported in association with cryptorchidism in a male dromedary camel (234). Additionally, the tumor was reported in the ovary of a 12-year-old female (11). The lesion consisted of an enlarged left ovary with a firm grayish-white nodule attached to and compressing the mesovarium structure. Microscopy depicted a circumscribed, encapsulated, lobulated, moderately cellular tumor. The neoplastic Leydig cells, large polygonal/polyhedral in shape and closely resembling interstitial cells, were organized in sheets, cords, nests, or acini, separated and supported by fibrovascular stroma. The tumor cells showed distinct boundaries, eosinophilic vacuolated to granulated cytoplasm, and eccentric ovoid to rounded nuclei with rare mitotic activity. The cytoplasm of neoplastic cells was vimentin positive by IHC.

##### 9.1.3.5 Sertoli cell tumors

Sertoli cell tumors, derived from Sertoli cells, secrete estrogen. A Sertoli cell tumor was diagnosed in an extremely large right ovary of a 16-year-old female dromedary (207). The tumor was a firm lobulated mass enclosed by the tunica albuginea of thick collagenous fibers. The cut surface depicted hemorrhage, caseous necrosis, occasional calcification, and variable-sized cavitation filled with serous fluid. Histopathology showed tumor cells arranged in solid, cord-like trabecular or spherical structures with cystic tubules in the tumor center. The endometrium displayed cellular hypertrophy, which might be associated with estrogen secreted by the tumor. Sertoli cell tumors were very rare in male camels, with only one report (211).

##### 9.1.3.6 Sertoli–Leydig cell tumor

Sertoli–Leydig cell tumor (androblastoma/arrhenoblastoma) is a rare, benign or malignant mixed sex cord-stromal testosterone-secreting tumor. It was reported in one left and one right ovary adjacent to the rete ovarii in two old dromedaries (207); in the ovary of an adult female dromedary (150); and in the right ovary of an 8-year-old primipara non-lactating dromedary with a history of conception failure (7). The US revealed enlarged, mostly homogeneous areas, sometimes with hypoechogenicity (7). Grossly, the tumor presented as small, whitish, firm, rounded nodules linked by stalks to the ovary (150) or as a large, smooth-surfaced, greasy shiny, bulging mass in the ovary, displaying multiple cavities when cross-sectioned (7). Histopathology depicted spindle Sertoli cells as well as Leydig cells (7, 150, 207). Thin layers of connective tissue stroma were observed. The ovaries were either inactive or hypoplastic (207). A Meyer type II Sertoli–Leydig cell tumor, with intermediate differentiation, was diagnosed in one case (7) with possible hormonal activity (229). Most tumor cells uniformly express alpha-inhibin in the cytoplasm (7) by IHC.

##### 9.1.3.7 Steroid cell tumor nos

Steroid cell tumor nos is a rare benign sex cord-stromal tumor manifested by symptoms of excess androgen production (235). The tumor was reported in the left ovary of an 8-year-old female dromedary (11). Grossly, the ovary was enlarged with firm white-gray to yellow nodules and a solid cut surface. Histopathology revealed multiple non-encapsulated foci of medium to large round to polygonal neoplastic cells. These cells were arranged in nests, sheets, or cords, obliterating and replacing the normal structure of the ovarian cortex while intervening with thin fibrovascular stroma. The neoplastic cells contained abundant foamy, clear, eosinophilic, or pigmented cytoplasm and small eccentric, round to oval, granulated nuclei. These tumor cells were surrounded by a thick layer of streaming spindle theca cells. The cytoplasm of tumor cells was positive for vimentin and melanin A and negative for inhibin.

#### 9.1.4 Ovarian adenomas

Ovarian adenomas are benign tumors of rare occurrence and, unlike germ cell and sex cord-stromal tumors, originate from the surface epithelium lining (236). Several types of adenomas have been reported in dromedary ovaries. Eight papillary adenomas in both ovaries were reported in older dromedary females (207); one case of ovarian adenoma was reported in a non-pregnant adult female (223); and two cases of unilateral ovarian papilliferous cystadenoma and cystic adenoma were grossly identified in abattoir specimens obtained from adult dromedary females (237). The ovarian adenomas constituted firm, nodular lobulated masses that contained cysts of different sizes filled with serous fluid, separated by whitish dense connective tissue septa (223). The histopathology of the papillary adenoma showed hyperplastic rete ovarii. The ovarian medulla was irregular and extensively replaced by tubular papillae and acini with cuboidal or columnar lining, containing basophilic nuclei, eosinophilic cytoplasm, and few fat droplets. The acini of tumor cells were packed with copious, PAS-positive pale eosinophilic secretions (207). In another case, the ovarian adenoma showed cystic compartments of varying sizes filled with eosinophilic fluid and lined by low cuboidal epithelium (223). Furthermore, another case of cystic ovarian adenoma exhibited large focal areas arranged in an alveolar form (237). However, the papilliferous cystadenoma displayed cystic structures containing pale eosinophilic mucinous substance. These structures were lined with simple cuboidal to columnar cells with vesicular cytoplasm, arranged in papillary or alveolar forms (237).

#### 9.1.5 Ovarian fibroma

Ovarian fibromas were reported in four healthy adult barren dromedary females (208) and in two additional adult non-pregnant dromedary females (228). The tumors were small, whitish, hard, round masses bulging from the ovarian surface. The tumors displayed bundles of interlacing spindle fibroblastic cells with connective tissue collagen fibers. Focal edema was reported in some cases.

#### 9.1.6 Ovarian hemangioma

Four cases of hemangioma were reported in adult non-pregnant dromedaries (208, 228, 238). The tumors were small, firm, dark to brown in color, with a smooth surface and prominent circular masses on the ovarian surfaces, with blood oozing from the lesions. Histopathology revealed hemangiomas showing contiguous congested blood vessels separated by stroma of fibroblastic connective tissue with a few macrophages (208, 228).

### 9.2 Uterus

#### 9.2.1 Malignant tumors

Adenocarcinomas were reported in the endometrium of two adult (150) and one elderly dromedary female (208). Additionally, one case involved an infertile, anemic, lymphocytopenic/monocytopenic multiparous adult female with a history of difficult mating and vaginal bleeding after copulation (161). Rectal palpation revealed diffuse masses that were thick and firm (161). The US disclosed homogeneous echogenic activity, sometimes accompanied by multiple areas of hypoechogenicity. The cut surface of adenocarcinoma displayed many firm, whitish, rounded, or gray, thick nodular masses (150, 161, 208). Various lesions were observed, including scirrhous nodules adherent to the uterine wall and peritoneum (208), cystic structures with homogeneous eosinophilic fluid in a greasy thickened uterine horn (150), and ulceration and necrosis of the uterus (161). Adenocarcinomas showed different microscopic appearances. In one case, there was complete morphological damage to the uterine glandular epithelium due to massive infiltration of poorly differentiated epithelial cells with large vesicular nuclei, conspicuous nucleoli, and average mitotic activity (208). The tumor metastasized via blood and lymphatics to the salpinx mucosa and urinary bladder. In another case, many cystic adenocarcinoma structures of variable sizes and shapes were observed (150). These were lined by two or more layers of neoplastic cells with copious cytoplasm, vesicular or hyperchromatic nuclei, and low mitotic activity. The tumor showed dense connective tissue stroma infiltrated by multiple solid structures of tumor cells. Some cysts accumulated homogeneous eosinophilic secretions. In a third case, endometrial adenocarcinoma with papillary glands of variable sizes was reported (161). These were lined by well-differentiated neoplastic cuboidal cells, along with myometrium invasion by irregularly shaped glands that secreted mucin. The IHC disclosed diffuse expression of EMA and Carcinoembryonic antigen (CEA) markers (161).

#### 9.2.2 Benign tumors

Leiomyoma was reported in the uteri of two mature female dromedaries (150). The tumor grossly displayed a large, firm, pinkish-brown, pedunculated single mass attached by a stalk to the uterine wall. Light microscopy revealed irregular bundles of smooth muscle fibers with cigar-shaped nuclei, haphazardly running and interlacing, with minimal connective tissue stroma. Lipoma was described in a mature female Arabian camel (150). The tumor mass was rounded, multilobulated, soft, pedunculated, whitish in color, and attached to the uterine serosa. Histopathology depicted well-differentiated, large-sized, irregular adipocytes forming lobules separated by thin septa of vascularized connective tissue. Hemangioma was reported in the uteri of eight adult barren dromedary females (208). The tumors showed many blood vessels filled with blood, packed within the endometrium and lined by endothelial cells.

### 9.3 Vaginal and cervical tumors

Cervical cancer is the fourth most fatal cancer in women, with Human papillomavirus (HPV) being the primary culprit (239, 240). Siddiqui and Telfah (9) presented and described vaginal tumors in female dromedaries during their work in camel surgery. These tumors were single or multiple circumferential growths cranial to the lips of the vulva and were mostly located at the vestibulovaginal junction, hindering penile intromission and causing infertility.

#### 9.3.1 Adenocarcinoma

Vaginal adenocarcinoma was diagnosed in an adult female dromedary showing regular estrous cycles without conception and bled after mating (23). Other reports included nine cases of vaginal adenocarcinomas and two cervical adenocarcinomas, which led to narrowing or obliteration of the passage to the uterus and bleeding (161). Hematological data showed anemia, lymphocytopenia, and monocytopenia. US disclosed homogeneous echogenic activity, but sometimes revealed multiple areas of hypo-echogenicity (161). Grossly, Ali et al. (161) reported hard, whitish, nodular tumors with ulceration and necrosis, whereas Ali et al. (23) reported numerous rounded masses of varying sizes, with clotted blood, connected to the vaginal wall. One of the cases showed metastasis to the iliac lymph node, mesentery, and liver (161). The liver was enlarged with multiple firm nodules, the mesentery displayed numerous small nodules arranged in a rosary-like pattern, whereas the lymph node was simply enlarged. The histopathology of the adenocarcinomas in the vagina, cervix, and other metastatic organs displayed glands of variable size lined by well-differentiated or, rarely, moderately differentiated cuboidal cells (161). Focal hemorrhage was observed. IHC disclosed diffuse expressions of EMA and CEA tumor markers.

#### 9.3.2 Lipoma

A lipoma was reported in an infertile adult dromedary with regular estrous cycles (23). The lesion, diagnosed by rectal examination, vaginal exploration, and rectal ultrasound, comprised a large, single, soft mass lodged in the lateral vaginal wall a few centimeters from the vulva, hindering penile intromission. Similarly, a lipoma was reported in another infertile adult female dromedary (9). This tumor was a large lobulated mass on the ventral commissure of the vulva, also hindering intromission.

### 9.4 Preputial tumors

These male tumors occur on the lateral aspect of the prepuce and can be of any type, including papillomas, fibromas, squamous cell carcinomas, and mast cell tumors. The tumors can be small or large enough to interfere with movement or push the preputial opening, thereby impeding mating. Surgical procedures have been described for treatment (9).

### 9.5 Mammary gland tumors

The mammary gland tumors can present as solitary or multinodular lesions and can be malignant, benign, or exhibit both types concurrently. The tumor may involve the entire gland or only a portion of the glandular tissue, with or without involvement of the nipples. Generally, mammary gland tumors are infrequent in camelids (241). Adenocarcinomas (209), carcinoma (178), mammary papillary carcinoma with fibroadenoma (116), and intracystic papillary carcinoma (IPC) (210) have been reported in the dromedary.

#### 9.5.1 Adenocarcinomas

One case of simple tubular adenocarcinoma and twenty cases of mammary adenocarcinomas were reported in adult dromedary females (116, 209). Histopathology of the adenocarcinomas revealed pleomorphic cells arranged in tubular or ductal forms, with pleomorphic nuclei and occasional mitotic figures. The tumor cells were supported by moderate stromal tissue with sclerotic areas and varying numbers of lymphocytes and plasma cells (209).

#### 9.5.2 Carcinomas

Poorly differentiated mammary gland carcinoma with metastatic lesions was reported in a captive multiparous 15-year-old dromedary female (178). The camel was bright, had a normal appetite, good body condition, and normal routine blood tests. It exhibited unilateral enlargement of the right fore and hind quarters of the mammary gland. The lesions were discrete, cool, and firm large masses on each quarter, with cord-like structures extending toward the inguinal area. Biopsies were accompanied by severe bleeding, and the animal consequently died. Necropsy revealed a firm, pale, homogeneous mass. Duct or acini remnants depicted poorly differentiated epithelial-like cells with heavy vasculature, metastasizing to the lungs and pulmonary lymph nodes. Additionally, a mixed tumor of papillary carcinoma and fibroadenoma was reported in a non-lactating 9-year-old pregnant dromedary (116). The udder was swollen and hard, with one distended quarter displaying two reddish masses. The larger mass was hard, circumscribed, non-encapsulated, and connected to nodules blocking the teat canal, whereas the other was an average-sized, cauliflower-shaped, and loose mass. Microscopically, the udder tissue was completely obliterated by invasive tumor lesions. There were small compartmental nodules showing papillary fronds with secondary or tertiary papillae based on a congested fibrovascular core. The papillae were lined by pleomorphic, poorly differentiated pseudostratified cells with vesicular nuclei. Micropapillary patterns were occasionally observed. The connective tissue stroma was either dense and diffuse or myxomatous. Necrosis and leukocyte infiltration were frequent. Moreover, fibroadenoma with many branching tubules or ducts embedded in a myxomatous or extensively fibrotic stroma was reported (210). The tumor cells were pleomorphic with pleomorphic nuclei along with calcospherite deposits. The IHC of papillary carcinoma showed positive nuclear staining for estrogen and negative staining for progesterone receptors, whereas fibroadenoma was negative for both receptors. Furthermore, intracystic papillary carcinoma (IPC) was reported in a non-pregnant, non-lactating adult dromedary female with a chronic udder lesion (210). The udder was hard and swollen, with an enlarged left hind quarter teat obliterating the teat canal. The cut surface of the udder was lobulated, gray-white, and arborescent in shape, delineated by fibrous septa. Histopathology displayed complete destruction of normal udder tissue, replaced by tumor masses of dense primary, secondary, or tertiary branches of papillae, entrapped in cysts and supported by a fibrovascular core. Various sizes of fibrous tissue stroma were evident. The xerophytic intracystic papillary lesions exhibited pleomorphic, poorly differentiated epithelium with vesicular or ground-glass nuclei. Invasion of lymphatics and infiltration of tumor cells into normal acini were observed. Mononuclear cell infiltration and dystrophic calcification were occasionally noted in the tumor stroma. Using IHC, tumor cells were positive for CK5/6 and negative for estrogen and progesterone.

#### 9.5.3 Fibromas and fibroadenomas

Three cases of intraductal fibroma were reported in the mammary glands of dromedary females (209). The tumor consisted of papillary fibrous growth with intraductal protrusion. In addition, eight cases of fibroadenoma were reported in the mammary glands of adult dromedary females (116). Grossly, the lesions were firm, enlarged, and showed a whitish-gray cut surface. In histopathology, the tumor cells displayed narrow or dilated tubules with single or multilayers of haphazardly organized pseudostratified pleomorphic cuboidal cells and occasionally contained homogeneously pink material. The tumor cells showed pleomorphic nuclei without mitotic activity. The tumors had moderate fibroblastic stroma, sometimes sclerotic, with infiltration of plasma and lymphocytic cells. Hyperprogesteronism may be implicated in tumor formation (242). Moreover, fifteen cases of cystic adenomatous hyperplasia, representing benign tumors, were also reported (116).

### 9.6 Summary of tumors relative frequencies in the reproductive system

The tumors of dromedary camels in the reproductive system were prevalent, presenting a relative frequency of 23.02% (Supplementary Figure S1). Carcinoma was the most common, with a relative frequency of 5.87%. The tumors were mainly diagnosed in the mammary gland, vagina/cervix, and uterus, but their etiology remains unknown. Mammary, cervical, and vaginal cancers are very common in women and are primarily associated with HPV (239, 240). Prostate tumors, which primarily occur in aged men, were not reported in camels. This may be due to the few stud camels kept for breeding, while the rest are killed, castrated, or disposed of at an early age. Ovarian tumors in camels were mainly benign, with teratomas comprising the main germ cell tumor, showing a relative frequency of 4.25% but having little or no effect on fertility (221). The relative frequency of malignant germ cell tumors (0.59%), including dysgerminoma (0.15%) and seminomas (0.44%), as well as malignant sex cord stromal tumors (granulosa cell tumors at 0.29%), was low, constituting 0.88% and aligning with previous reports (10). On the other hand, ovarian cancers are highly prevalent in women, ranking sixth in incidence and fourth in mortality among all tumors (213). The relative frequency of sex cord stromal tumors in dromedaries accounted for 3.37% and was associated with infertility and hormonal imbalance (229–231). Future research needs to be conducted by breeding centers to select breeding animals free from oncogenes using state-of-the-art diagnostic equipment and cutting-edge cancer biomarkers aided by AI. The relative frequency of benign tumors was variable, with 4.99% adenomas, 1.76% hemangiomas, 1.47% fibromas, 0.44% lipomas, and 0.29% leiomyomas, all with minimal reproductive effects.

## 10 Urinary system

The tumors of the urinary system were comparatively rare in the dromedary camel (Supplementary Table S7).

### 10.1 Kidney

#### 10.1.1 Renal cell carcinoma

Renal cell carcinoma was reported as a large, ovoid expansive mass in the right kidney of a dromedary (243). The cut surface was light brown with foci of necrosis. The histopathology of the tumor displayed dense papillae or ramified papillary fronds projecting into micro-cysts, as solid masses or tubules with poorly delineated margins invading the renal medulla. The papillary renal carcinoma exhibited cellular atypia with a rich vascular stroma. The tumor cells were cuboidal or pleomorphic with hyperchromatic nuclei and few mitotic figures. Additionally, a large honeycomb renal cell carcinoma was reported (10). The tumor histopathology showed well-differentiated tubules separated by cellular stroma. Furthermore, renal cell carcinoma was reported in a 13-year-old female dromedary with abdominal pain, hematuria, chronic weight loss, and anemia (244). The US revealed a large, hypoechoic irregular mass in the parenchyma of the right kidney. Grossly, an extremely large cavitated, hemorrhagic irregular mass was observed. Histopathology displayed tubules lined by neoplastic cells with anaplastic nuclei. The tumor showed no metastasis or invasion.

#### 10.1.2 Nephroblastoma

Nephroblastoma (Wilm's tumor), an embryonic malignant renal tumor, was reported in a 6-month-old intact male dromedary presented with prolonged inappetence and weight loss (245). Necropsy revealed a large, well-demarcated, firm multilobulated renal neoplasm in the left kidney, whereas the right kidney showed polycystic dysplastic lesions and a prominent ductus arteriosus. Histopathology depicted extensive proliferation of primitive nephrogenic structures, mainly of blastemal epithelium and stroma. This type of neoplasia is common in children and linked to mutations in the Wilms' tumor suppressor gene (WT1). The condition is very rare in mammals other than monogastric animals (246, 247).

#### 10.1.3 Multicentric T-cell lymphoma

Multicentric T-cell lymphoma was diagnosed in a 7-year-old female camel presented with inappetence, weight loss, polydipsia, polyuria, and low urine specific gravity (163). The WBC, BUN, and serum creatinine levels were high. Necropsy revealed multiple pale neoplastic nodules of varying sizes distributed in both kidneys and scattered to other organs, along with pale and enlarged perineal lymph nodes. The IHC was CD-3 positive and 79a negative.

#### 10.1.4 Adenoma

Renal cell adenoma was reported in an adult dromedary (248). The tumor had a mixed tubule-papillary pattern. Histopathology displayed multiple foci of proliferative tubules or papillae with a well-vascularized loose connective tissue stroma projecting into cyst-like cavities. The tubules and papillae were lined with layers of cuboidal cells exhibiting extremely rare cellular and nuclear pleomorphism and cellular atypia. The IHC illustrated strong positive cellular staining for cytokeratin.

#### 10.1.5 Leiomyoma

Leiomyoma was reported in a 2-year-old male dromedary (169). The case presented with a very large pedunculated abdominal mass, decreased appetite, constipation, abdominal pain, and weight loss. Laboratory analysis showed hematuria, low hematocrit, high WBC, neutrophils, and low total protein. The US displayed echogenic deposits and anechoic zones in the left kidney, spleen, and abdominal wall. A large hypoechoic and well-vascularized mass was observed just below the left kidney and spleen. Exploratory puncture showed a very large firm pedunculated mass attached to the abdominal wall lateral to the rumen. Peritoneal tumor masses were also detected in the wall of the left abdomen. Histopathology confirmed leiomyoma in the kidney and other organs. Thickening of blood vessel walls, extensive areas of hemorrhage, necrosis, and infarctions were observed. The tumor seemed to spread from smooth muscle to different organs through expansion. Tharwat and Al-Sobayil (249) reported, via transrectal US, an echogenic mass in the medulla of the left kidney of a male dromedary with uneven thickening of the urinary bladder mucosa. In another case, a large encapsulated echogenic kidney mass was reported using US in a female dromedary with a history of weight loss (249). These two lesions were most likely kidney tumors but were not confirmed by histopathology.

### 10.2 Urinary bladder and urethra

#### 10.2.1 Adenocarcinoma

Urinary bladder adenocarcinoma cell emboli metastasizing from a primary adenocarcinoma originating from the endometrium were reported in an old dromedary (208). Histopathology depicted poorly differentiated epithelial cells with vesicular nuclei, prominent nucleoli, and average mitotic activity. Tumor emboli were identified within the blood and lymphatic vessels of the urinary bladder, indicating metastasis.

#### 10.2.2 Fibroma

Urethral fibroma was reported in a male dromedary with consequent urine retention (170). Additionally, a fibroma was diagnosed at the neck of the urinary bladder (250).

### 10.3 Summary of tumors relative frequencies in urinary system

The tumors in the urinary system of the dromedary camel were low (Supplementary Figure S1), with a relative frequency of 1.47%. The tumor types and their relative frequencies were as follows: carcinomas (0.59%), lymphosarcoma (0.15%), and nephroblastoma (0.15%). The relative frequencies of benign tumors comprised 0.29% (fibroma), 0.15% (adenomas), and 0.15% (leiomyoma). Only one case of nephroblastoma (Wilm's tumor) was reported in a young camel. Generally, nephroblastoma is very rare in cattle, sheep, and goats but common in children and monogastric animals (8, 239, 246, 247). Bladder cancers were extremely rare in the dromedary, with only one reported case. However, bladder cancer is common in humans exposed to risk factors such as tobacco smoke, mycotoxins, and gold mining (28, 239).

## 11 Nervous system

There were very few tumors reported in the nervous system of the dromedary (Supplementary Table S8).

### 11.1 Neuroectodermal tumor

A primitive neuroectodermal tumor (pPNET), a malignant neoplasia, was reported in a castrated 9-year-old dromedary (166). The camel was weak and ataxic and proceeded to lateral recumbency with hind leg paralysis. Necropsy findings showed multiple tumor masses in the liver and a mass in the third vertebra of the lumbar region, causing spinal cord compression. Histopathology of the liver and vertebral lesions revealed primitive cells arranged in perivascular pseudo-rosettes resembling ependymal cells, with a few neuroblasts arranged in Homer-Wright rosettes (spoke-and-wheel shaped). The neoplastic cells were positive for vimentin and variably positive for neuron-specific enolase and glial fibrillary acidic protein (GFAP) by IHC, reflecting their origin as primitive multipotent neuroepithelium. These findings pointed to a pPNET of neuroplastic glial and ependymal cells similar to the Ewing's sarcoma family, resembling that in adult humans. The tumor likely originated from the spinal cord and metastasized to the liver.

### 11.2 Multicentric schwannoma

Schwannoma is usually a slow-growing benign tumor composed of homogeneous capsular masses of Schwann cells, which typically reside outside peripheral nerves and are involved in the formation of the myelin sheath. A multicentric schwannoma was reported in a 4-year-old dromedary camel (167). The tumor was composed of numerous small nodular masses in different organs (see Section 5.9; tumors of the digestive system) and was diagnosed by IHC (positive for S100 and negative for CK, c-kit, and CD34) and confirmed by electron microscopy. Another case of schwannoma was reported in a dromedary, presented as a clinical case, with diagnosis confirmed by Alsobayil et al. (10).

### 11.3 Papilloma of choroid plexus

Papilloma of the choroid plexus was diagnosed in a 6-year-old male dromedary showing incoordination, paresis, and eventual paralysis of the hind limbs (251). Necropsy depicted a small, well-circumscribed pinkish-gray mass in the dilated fourth ventricle. Hydrocephalus, presented by numerous cystic cavities of varying sizes, was observed in the brainstem and cerebral hemispheres. Edema, gelatinization, and necrosis were noted in some lumbosacral spinal nerves. Histopathology revealed non-encapsulated, densely proliferated branches of papillary fronds covered with clusters or nests of well-differentiated cuboidal/columnar epithelium. The fronds rested on a delicate, well-vascularized fibrovascular stroma infiltrated by mononuclear cells. The tumor cells showed round to oval hyperchromatic nuclei and eosinophilic cytoplasm. Unlike the hobnail appearance of choroid plexus epithelium, the cell luminal surfaces were straight and lacked cilia. Multiple areas displayed focal vacuolation. The malacic changes were accompanied by glial cell proliferation, perivascular cuffing, neuronal degeneration, and necrosis. The tumor cells were positive for cytokeratin 7 and negative for GFAP by IHC.

### 11.4 Summary of tumors' relative frequency in the nervous system

The tumors of the nervous system reported in the dromedary (Supplementary Figure S1) were generally few in types and low in relative frequency (0.59%). The tumor types in the nervous system showed relative frequencies of 0.29% (Schwannoma), 0.15% (Papilloma), and 0.15% (primitive ectodermal tumor). Meningiomas are not reported in the dromedary but are relatively common in humans and dogs (252).

## 12 Skin and integument

Skin and subcutaneous tumors are very common in domestic animals (8, 252, 253). Different types of skin tumors (Supplementary Tables S9a, b), mostly carcinomas, sarcomas, papillomas, and teratomas, have been reported in the skin and integuments of dromedaries (4, 9, 10, 114).

### 12.1 Carcinomas

Squamous cell carcinoma (SCC) is common, whereas basal cell and sebaceous carcinomas are rarely reported in the dromedary. SCC was reported in the skin of the left forelimb, above the coronet, of a 9-year-old male (254), in 12 adult dromedaries (6), as a clinical case (8), and in a large number of toenails of different breeds, predominantly on the medial aspects of the fore and hind limbs (255, 256). In addition, the tumor was reported in a swollen right hock joint of a 7-year-old male with a chronic granulomatous wound infiltrating the tarsal and proximal metatarsal bones (202). Moreover, the tumor was diagnosed in the maxilla and neck areas in two other cases (186), in a hemorrhagic ulcerative scrotal skin (257), and in two cases around the cornea (143). The SCC lesions were mostly aggressive, firm, and large, with oval, round, or irregular shapes, featuring a nodular rough surface and a hard grayish or pale pink cut surface. The lesions might also be irritable, fragile, painful, bleed easily, suppurate, or present as just superficial wounds (6, 143, 186, 202, 254–256). Histopathology showed well-differentiated (202), moderately differentiated (255), or poorly differentiated carcinomas (254). In the well-differentiated and moderately differentiated types, keratinized pleomorphic squamous cells with moderate cell atypia and low mitotic activity, entrapping central concentrically laminated eosinophilic keratin pearls, were observed (6, 143, 202, 255). The tumor cells deeply invaded the epidermis and were surrounded by desmoplastic fibrovascular stroma, which might show necrotic areas and infiltration with inflammatory cells (202, 255, 257, 258). The poorly differentiated SCCs showed scanty or non-keratinized large squamous neoplastic cells with high mitotic figures. Cellular and nuclear atypia were observed, but horn pearls were few or absent, and epithelial keratinization was very scanty. The epithelial masses were surrounded by pale-staining desmoplastic stroma with polymorphic inflammatory cells (6, 254, 255). Metastasis, invasion, or infiltration of SCCs from skin to other organs were observed (202, 258). The coexistence of multiple tumors in the skin is a rare occurrence. However, SCC and sarcoma were diagnosed at the ventral sternum pad in an old male dromedary with a large wound and subluxation of the front left fetlock joint (258). Severe unresponsive anemia and leukocytosis were evident (258). The SCC was well differentiated.

Trabecular and nodular variants of basal cell carcinoma (BCC) were reported in a 10-year-old female dromedary with a hard, lobulated, insidiously growing swelling at the right maxilla with intact skin (259). Grossly, the lesion was a circumscribed lobular mass in the dermis and subcutis with involvement of the posterior maxillary sinus. Histopathology displayed large groups of uniformly medium-size, infiltrative, well-differentiated basal cells of the epidermis with scant cytoplasm, hyperchromatic nuclei, multiple nucleoli, and frequent mitotic activity, arranged in cords, columns, solid masses, or cell nests separated by fibrous stroma with variable collagen thickness. BCC is a non-metastasizing invasive malignant epithelial tumor possibly caused by exposure to solar radiation. Sebaceous carcinoma of the eyelid was reported in an adult male dromedary (260). The tumor appeared yellowish-white, ulcerative, and alopecic. Histopathology depicted lobules of pleomorphic tumor acinar cells with lipid globules and pleomorphic hyperchromatic nuclei, displaying many mitotic figures. Focal cellular necrosis was evident.

### 12.2 Sarcomas

Myxosarcoma was reported in seven male dromedaries in the skin of the scrotum, forelimb, chest, and back (257), osteosarcoma in the ventral side of the mandible, lymphosarcoma in the neck region, and fibrosarcoma in the ventrolateral abdomen of dromedaries (186). In general, the myxosarcoma lesions were firm, irregular, pale yellow, ulcerated, hemorrhagic subcutaneous nodules with a lobulated cut surface that contained mucinous liquid (257). On the other hand, the osteosarcoma, lymphosarcoma, and fibrosarcoma appeared as superficial swellings during clinical examination and US (186). The histopathology of myxosarcoma showed numerous fibroblasts with long fibrils within an abundance of myxoid stroma. Few stellate or spindle-shaped pleomorphic poorly differentiated cells with round or oval nuclei, multiple nucleoli, and few mitotic figures were observed. Circumscribed myxosarcoma foci were detected in the dermal layer of the scrotal skin, and extensive hemorrhage was noted. The tumor cells were vimentin positive. The histopathology of skin lymphosarcoma and osteosarcoma were similar to previous descriptions in Sections 7.1 (lymphosarcoma) and 8.2 (osteosarcoma).

### 12.3 Melanoma and melanocytoma

Melanoma, a malignant tumor, is uncommon in the dromedary. Cutaneous melanoma was reported in two adult (114) and one old male (261) dromedary. The tumor showed elevated alopecic, hyperpigmented/gray-black median to large cutaneous firm nodules, which may be ulcerated (114, 261). Histopathology displayed single or small nests of anaplastic pleomorphic malignant melanocytes located within the lower papillary dermis, bridged to the basal epidermis.

Two cases of benign melanocytoma were reported in the dromedary (114). Grossly, the tumor displayed large, brown to gray masses with intact alopecic epidermis. Histopathology revealed rounded melanocytes showing atypia with a substantial amount of intracytoplasmic melanin. Pleomorphic cells with varying nuclei and mitotic figures were infrequent. The cells were arranged either singly or in small nests and were located at the epidermal-dermal junction and the superficial dermis. Another case of benign uveal melanoma (melanocytoma) was reported (262). The tumor was characterized grossly by black discoloration of the eyeball. Light microscopy depicted numerous pleomorphic, intensely pigmented melanocytes infiltrating the internal surfaces of the iris, cornea, and ciliary body but not invading blood vessels.

### 12.4 Fibromas, papillomas, and papillomatosis

#### 12.4.1 Fibroma

Fibromas were reported in ten dromedaries (6). The tumors were ball-like swellings of variable size located on the neck, thigh, and fetlock joint. Moreover, a case of fibroma showing a painless, circumferential hard mass was reported at the right metatarsal area in an 8-year-old stud male dromedary (9). Two more cases of fibromas were reported in adult dromedaries. One of these involved a large mass occupying the whole chest pad, while the other was a large mass on the postero-ventral chest pad. These tumors were painful and prevented the animals from adopting an external posture, interfering with locomotion. El-Shafaey et al. (186) reported eight cases of fibromas from superficial swellings at different body sites of dromedary camels of various ages and sexes, identified using US. Microscopically, the tumors showed bundles of spindle-shaped interlacing fibroblasts separated by eosinophilic collagenous stroma (6, 186). Additionally, four (255) and 32 (256) cases of toenail fibromas were reported in the medial digits of fore and hind limbs. The fibroma masses appeared moist, hard, and painful. Light microscopy revealed that the tumors were characterized by thick skin layers with areas of acanthosis and spiny hyperkeratosis. Abnormal collagenous fibers deposited up to the subcutis were also observed.

#### 12.4.2 Papilloma

About 40 papillomaviruses (HPV types) are implicated in human cancer, including genital warts, cervical, anal, mouth, and throat cancers. Different strains of papillomaviruses are associated with various papilloma lesions in cattle, including BPV-1 and BPV-2, which cause fibropapillomas (i.e., skin warts and cancer of the urinary bladder), BPV-3 and BPV-6, which induce epithelial papillomas, and BPV-4, which is associated with upper alimentary tract papillomatosis and cancer. BPV-1, BPV-5, and BPV-6 are involved in papillomas and fibropapillomas of the teats (263). Interspecies transmission of papillomavirus strains has been reported between bovines and dromedaries (17, 130) and between equines and camelids (132).

Papilloma, a slow-growing, well-demarcated tumor, can occur at various locations, including the skin, lips, oral cavity, conjunctiva, cornea, penis, and vulva. Four cases (114) of squamous papilloma and one case (257) of another type of papilloma (186) were reported at different skin locations. Histopathology showed proliferation of skin layers with hyperkeratosis and parakeratosis at the stratum corneum, as well as hyperplasia and acanthosis at the stratum basale. The prickle cells of the stratum spinosum showed cytoplasmic vacuolation with rare mitotic figures. The tumor cells rested on proliferating dermal connective tissue stroma, often with congested capillaries (114, 257). Additionally, papilloma was reported in a 15-year-old male dromedary with keratoconjunctivitis, presenting as a pedunculated, nodular, firm mass in the left eye (130). It was characterized by third eyelid and conjunctival hyperemia, as well as grayish-white small pedunculated nodules at the ventrolateral part of the cornea. Biopsy specimens revealed marked acanthosis, hyperkeratosis, proliferation of rete ridges, and the presence of koilocytic changes at various epidermal layers. The prickle cells in the stratum spinosum displayed karyopyknosis and cytoplasmic vacuolation with basophilic intranuclear inclusions. The IHC specimens showed positive nuclear and cytoplasmic staining for bovine papillomavirus in the epithelium of the stratum basale and stratum spinosum, as well as for cytokeratin AE1/AE3, along with fibrocytes in the dermal layer.

#### 12.4.3 Papillomatosis

Papillomatosis is a mild disease, with most outbreaks reported in young camels up to 2 years of age (20). Outbreaks of papillomatosis were described in young dromedaries during the rainy season in several countries (18, 19, 21, 128, 129, 354). The tumor lesions were variable in size, primarily appearing as small dark gray raised nodules, sometimes dry and crusty, and either single or multiple, round to flat in shape. However, large nodules and, rarely, cauliflower-like rough horny warts were seen attached by narrow pedicles on the lips, nostrils, and submandibular skin (19, 21). Some camels presented lesions on the legs, inguinal/genital regions, ears, and eyelids (18). The lesions were associated with frequent scratching, inappetence, anemia, leukopenia, and neutropenia, lasting for 4–6 months (129). Histopathology revealed a thickened stratum granulosum and corneum with acanthosis, hyperkeratosis, and parakeratosis, along with deep proliferation of rete pegs into the slightly hyperplastic dermis (18, 19, 128). The stratum granulosum displayed individual cells or clusters of koilocytes with clear cytoplasm and large pleomorphic keratohyalin-like granules (hollow cells) and basophilic intranuclear inclusions. The dermis exhibited fibroblastic hyperplasia and connective tissue proliferation, along with infiltration of mononuclear cells. The papillomavirus (PV) was detected by transmission EM (18, 128) and by IHC antibodies (128, 129). Camelus dromedarius PV type 1 (CdPV1) was isolated from cauliflower-like lesions, and PV type 2 (CdPV2) from the round oval nodule, with their complete genomes characterized and grouped under Delta papillomavirus, DNA viruses. Both were similar to BPV, which is implicated in fibropapilloma in cattle, and were immunogenic.

Papillomatosis, possibly of a different PV type, was reported in 102 adult Iraqi dromedaries, whereas young animals were not involved (22). The papilloma lesions included focal solid painless self-limiting outgrowths with firm consistency and varying sizes, mostly located on the neck and brisket. The histopathology of the lesions was similar to that described in younger animals, along with depletion of hair follicles. Again, the disease was reported in a 15-year-old female dromedary (17). The lesion was very large, with wart-like projections at the right fetlock joint. It was a hard, gray-white tumor mass with scattered yellowish spots. Histopathological findings included elongated irregular papillary projections of hyperkeratotic, variably anaplastic epithelium over a core connective tissue stroma. In a few areas, the tumor cells showed a tendency to transform into nests of squamous cell carcinoma. There was cellular necrosis with infiltration of numerous neutrophils and a few mononuclear cells at the tumor margins.

#### 12.4.4 Fibropapilloma and myxopapilloma

Fibropapilloma was reported in two camels, aged 18 and 24 months, with a history of large growths in the metatarsal region (264). The lesions, enclosed by epidermis, were tough, lobulated, gray-white, and large, with homogeneous smooth connective tissue on the cut surface. Similar lesions of fibropapilloma were reported in the skin of a forelimb in a male (257) and as growth lesions on the udder of a female dromedary (186). However, these tumors were ulcerative and pale yellow. Additionally, four cases of fibropapillomas were reported (114) in dromedaries from a slaughterhouse, characterized by large, rough-surfaced gray nodules. Histopathology revealed thickened epidermal layers, acanthosis, orthokeratosis, and parakeratosis in the stratum corneum, whereas the rete ridges exhibited finger-like hyperplasia and projected deeply into the fibromatous tissue of the dermis (114, 257, 264). Variable-sized Koilocytes were observed, indicating PV infection. The dermal and subcutaneous layers displayed extensive fibroblastic proliferation within irregular collagenous connective tissue. The fibroblasts were neoplastic, spindle-shaped with pleomorphic nuclei, and arranged in long, interlacing, and whorled bundles. No hair follicles, sebaceous glands, or other adnexa were observed in the tumor's dermal layer. Myxopapilloma was diagnosed as superficial swellings in the ventral area of the abdomen in a dromedary camel (186) through clinical examination, US, and histopathology.

### 12.5 Adenomas

Mixed sweat gland adenoma, an eccrine type of pleomorphic tumor, was reported in a 12-year-old male dromedary (265). The tumor mass was hard, lobulated, grayish, and located lateral to the left masseter area, covered by skin. Histopathology revealed infiltration of the dermis and subcutis by disorganized adenomatous tubules bound by a thin connective tissue. Below the epidermis, cuboidal/polygonal tumor cells with indistinct borders and abundant faint vacuolated cytoplasm, or clear cells with small vesicular nuclei, were intermingled with spindle-shaped myoepithelial-like cells. Wide areas showed malignant transformation, with pleomorphic cells showing nuclear atypia and mitotic activity. Extensive comedo necrosis of tumor cells was evident in the adenomatous areas, along with infiltration of pustule-like neoplastic nests and leukocytes. Irregular osteoid trabeculae with multinucleated osteoclasts were scattered among the neoplastic structures. The interstitial stroma was abundant, with pale cells scattered irregularly. The epidermal layer exhibited ulcerations, acanthosis, degeneration, necrosis, vesicular formation, and leukocyte infiltration. Keratinized and unkeratinized undifferentiated epithelium forming hair bulb-like structures, along with blood vessels and lymphatics, were observed. In IHC, the neoplasmic cell cytoplasm was densely reactive for S-100, whereas the epidermal epithelium was positive for cytokeratin, and most tumor cells were positive for periodic acid–Schiff (PAS) stain. In addition, sweat gland adenoma was reported as a firm pendulating mass on the skin of the neck of a male dromedary (257). The lesion oozed hemorrhagic fluid during sectioning. Histopathology displayed a well-demarcated sweat gland adenoma replacing large areas of dermis and subcutaneous tissue. The mass was divided into lobules of variable size and shape, composed of flat neoplastic acinar cells in a dense fibrous stroma, with acinar lumens filled with eosinophilic fluid.

Sebaceous adenoma, formed of nodular alopecic skin masses, was reported in an adult camel (114). The tumor displayed a yellow or white color and a lobular appearance on the cut surface. Histopathology slides showed that the neoplasia was composed of numerous sebocytes with scant cytoplasm and hyperchromatic nuclei located at the periphery of many lobules, along with a few basaloid cells. Again, sebaceous ductal adenoma was reported in an adult camel showing cutaneous skin lesions (114). In histopathology, the tumor consisted of numerous sebaceous ducts of variable size containing keratin and sebum, with few normal sebocytes. Mononuclear cell infiltration was observed in the upper dermis.

### 12.6 Dermoid cysts

Dermoid cysts are slow-growing benign tumors formed as a congenital defect during the embryonal period, with some ectodermal elements remaining in the dermis or subcutis. They are observed at birth and increase over time (266). Generally, dermoid cysts are painless, mostly unilateral, soft, fluctuating, oval, with defined boundaries and variable diameters, sometimes acquiring very large sizes (266–269). Eleven (267) and 10 (266) unilateral cases, along with a few bilateral dermoid cysts, were reported in dromedaries of both sexes and various ages. These cysts were located at the proximal part of the neck, adjacent to the jugular vein, either attached to or pendulated from the skin. Moreover, multiple dermoid cysts were reported around the left carpal and fetlock joints in an adult male dromedary with a distorted knee joint (9). Grossly, the capsule was thick and uneven, dividing the internal surface into cavities filled with hair clumps, debris, greasy glandular secretions, and thin dark red fluid (266, 267, 269). In histopathology, the cysts showed unevenly thickened walls of dense irregular connective tissue collagen, lined by stratified squamous epithelium (266, 267). The cysts contained hair follicles, sebaceous and sweat glands, keratin desquamation, and secretions (266, 267, 269). Furthermore, a corneal dermoid cyst was diagnosed in a 10-year-old male dromedary (270). The mass was a large, peduncle-shaped, unilateral nodule on the cornea of the left eye, causing copious lacrimation, edema, and bleeding.

### 12.7 Lipoma

Lipomas are common in dromedary skin. A case of a lipoma attached to the hock joint was reported in a female dromedary (6), and another was noted in a 7-year-old male dromedary at the ischioanal fossa, causing difficulty in defecation (174). Two cases were reported; one at the scrotum of a male and the other at the ventral abdomen of a female dromedary (257). Additionally, two cases were found in the subcutaneous skin (114), and one bilateral case was observed just above the tail base in a 10-year-old female (271). The tumors were painless, oval, soft, firm, or hard, gray-white-yellow in color, and could be circumscribed, encapsulated, or non-encapsulated masses, sometimes with a rough surface (6, 114, 174, 257). They could also be enormously large and pedunculated (271). The cut surface showed many lobular yellow or whitish masses. In histopathology, the tumor displayed well-differentiated, pleomorphic, polyhedral adipocytes separated into lobular structures by trabecular collagenous stroma extending from the connective tissue capsule (6, 174, 257, 271).

### 12.8 Rare tumors

Benign intramuscular myxoma, a mesothelial tumor located deep within striated muscles, was reported at the anterior aspect of the hock joint of an 8-year-old female dromedary (206). The tumor was a large solid white mass with muscular infiltration and was covered by dark skin. Histology revealed a tumor of low cellularity, primarily composed of stellate to spindle-shaped fibroblasts with scant cytoplasm, small hyperchromatic nuclei, and very low mitotic activity. The cells were loosely organized within a copious myxoid matrix, with focal hypercellular areas, thickened blood vessels, and eosinophilic infiltration. Another rare tumor, spiny keratoderma, was reported in multiple toenails of dromedaries (255, 256). The tumor was hard, painless, and non-aggressive. The histopathology displayed thick compact columns of parakeratotic spiny horns continuous with the stratum granulosum and with an abrupt transition to the orthokeratotic stratum corneum layer. In addition, a pyogranuloma located in the retropharyngeal region was reported as superficial swellings in a dromedary (186). The tumor was diagnosed through US and histopathology. Moreover, another rare case of a unilateral intraocular tumor mass was reported in the left eye based on clinical examination and US (272). The mass was hyperechoic with well-defined borders, but the tumor type was not specified.

### 12.9 Skin tumors reported but without full investigation

Some skin tumors were surgically excised without further investigation. These included a large pedunculated growth on the ventral aspect of the tail in a stud male dromedary (9). Similarly, a large firm tumor mass on the anterioventral side of the chest pad, showing progressive growth, was reported in an 8-year-old male dromedary (273). Additionally, mastocytoma and fibroma were reported in the dromedary camel without further details (191).

### 12.10 Summary of tumors' relative frequencies in skin and integument

The relative frequency of skin and integumentary tumors (54.84%) was the highest (Supplementary Figure S1) among all tumors reported in camels, mainly attributed to skin exposure to tumor risk factors. The relative frequency of carcinomas was highest (24.93%) in the skin, with the majority of cases attributed to toenail carcinomas of the fore and hind quarters. This may be due to chronic irritation caused by hot sand and UV light on the fleshy toes/nails during prolonged sun exposure while browsing. Toenail tumors are uncommon in other domestic animals (8). However, basal cell and sebaceous carcinomas associated with exposed skin or light hair coats to UV light were rare in camels (149). The relative frequencies of benign tumors such as fibromas (8.65%), papillomas (6.16%), papillomatosis (4.70%), and teratomas (3.52%) were high and similar to previous reports (10, 267, 273). On the other hand, the relative frequencies of keratoderma (2.20%), sarcoma (1.61%), lipoma (1.03%), adenomas (0.59%), lymphoma/lymphosarcoma (0.29%), myxoma (0.15%), and pyogranuloma (0.15%) were comparatively low. Unlike equines, ruminants, and small animals (273), the relative frequencies of melanocytoma (0.44%), melanoma (0.29%), and mast cell tumors (0.15%) were low in camels.

## 13 Neoplasia of endocrine glands in the dromedary

### 13.1 Adenomas and carcinoma of thyroid gland

These tumors are generally rare in dromedaries, apart from those reported in the ovaries (11, 207, 208). Papillary thyroid carcinoma and follicular adenoma were reported in the thyroids of two apparently healthy adult male dromedaries (274). In addition, follicular adenoma and papillary adenoma of the thyroid were described in a 1-year-old male dromedary with an apparently enlarged thyroid (275). Grossly, the thyroid adenoma showed whitish solid well-demarcated small nodules in one case (274), whereas in the other case, the thyroid lobes showed follicular cysts (275). US of the thyroid carcinoma and adenoma depicted hypoechogenicity and sometimes heterogeneity of the thyroid parenchyma (274). Microscopically, the follicular adenoma consisted of micro-follicles surrounded by a thin capsule of fibrous tissue. The papillary adenoma exhibited a papillary structure projecting into a colloid cyst. Follicular and papillary adenomas were negative for CK19, GAL3, calcitonin, CD56, and HBME1 tumor markers (275).

### 13.2 Summary of tumors' relative frequencies in the endocrine system of dromedary camels

The relative frequency of endocrine system tumors (0.59%) was low (Supplementary Figure S1). However, tumors in the male and female reproductive systems were not included, as these were previously covered in Section 9 (Reproductive System). The relative frequencies of thyroid tumors included 0.15% for papillary thyroid carcinoma and 0.44% for thyroid follicular/papillary adenoma. Unlike other species (8, 252), tumors of the pituitary, parathyroid, adrenal, and pineal glands were not reported in the dromedary camel (4, 9, 10). Thyroid cancer is common among humans, where its occurrence ranks about 6th in women and 13th in men (188, 276); however, other animals develop thyroid tumors with variable frequency (252).

## 14 Clinical approach, diagnosis, and treatment of tumors

### 14.1 Clinical approach to tumor diagnosis in the dromedary

During routine clinical examination of suspected tumor cases, the age, sex, breed, coat color, and body condition score are recorded (6). Vital signs, including body temperature, heart rate, and respiratory rate, are taken. Mucosa and conjunctiva are checked for jaundice or anemia. Diarrhea, constipation, urine retention, abnormal secretions and excretions from body orifices, and unusual body odors are recorded (82). Abnormal posture, breathing, gait, neurological signs, cachexia, edema, and anasarca are documented. Large and small rounded, flat, or cauliflower nodules and wounds around the mouth, nares, jaws, chest pad, eyes, toenails, tail, and outer body surfaces are thoroughly examined (4, 9, 12). Blood samples are collected by jugular venipuncture from suspected tumor cases in plain, heparinized, and anticoagulant (K3-EDTA) vacutainers for hematology (anemia, leukosis), flow cytometry (leukemia/lymphoma), and biochemistry, including serum tumor markers (180). Fine needle aspirate smears are used for tumor cytology screening. Rectal palpation, radiography, and nuclear scintigraphy (4, 179), as well as ultrasonography (23, 272) and ophthalmoscopy (143), are performed when required, depending on their availability. Animals with painful, hopeless, and incurable cancer conditions are euthanized. Post-mortem (PM) examinations are performed on all deceased animals suspected of cancer if suitable for examination (9, 12, 191). Gross descriptions of suspected tumor lesions, i.e., body location, color, size, shape, consistency, distribution, and cut surface, are recorded after thorough examination of all body systems, lymph nodes, body cavities, and body fluids (4, 9). Tissue specimens are taken from biopsy, surgery, and necropsy after gross examination (4, 9, 266) and are immediately fixed in 10% buffered formal saline, processed, embedded in paraffin wax, sectioned to 4–5 μm thick, and stained for routine Hematoxylin & Eosin (H&E), special stains, and IHC. Specimens for genetic analysis are immersed immediately in liquid nitrogen and kept at −80 °C pending analysis. Tissue specimens from abattoirs for tumor surveys are obtained in collaboration with the veterinarians in charge. Tumor diagnosis can be attained by collating all the above information. Future research and treatment of tumors in humans and animals will largely depend on AI-based techniques.

### 14.2 Hematoxylin and eosin routine stain

Hematoxylin & Eosin (H&E) is the first routine stain for histology/histopathology (277). The H&E stain detects almost all types of tumors and provides information about tumor cell types and atypia; cytoplasm color intensity; nuclear size, number, and shape; mitotic index; and nuclear atypia (278). Additionally, H&E displays the arrangement of tumor cells, whether in solid sheets, adenomatous structures, peripheral palisading, or rosette shapes, and characterizes the tumor stroma as thin loose connective tissue, myxoid, or desmoplastic. It also reveals the presence of keratin pearls, mucin, and other proteinaceous materials, as well as the presence and type of inflammatory cells and other pathological changes (279).

### 14.3 Special stains

Special stains can also aid in tumor diagnosis in the dromedary. For example, oil red is used in lipoma, liposarcoma, and tumefactive demyelination (113). Masson's Trichrome assists in differentiating between fibroblastic and myofibroblastic mesenchymal stem cell tumors, where in leiomyoma, the muscle cells stain red, while in fibroblastic tumors, the collagen fibers stain green or blue (113, 182). Other stains include periodic acid–Schiff (PAS) for pleomorphic adenoma (265), Congo red for tumors with amyloid accumulation, such as some renal, lung, skin cancers, and basal cell carcinomas (257). Moreover, Alcian blue followed by PAS is used to detect neutral mucins in adenocarcinoma; van Gieson's stain is used for collagen staining in hemangiosarcoma (151); and Mallory's phosphotungstic acid hematoxylin is used for muscle cross-striations in rhabdomyosarcoma (182).

### 14.4 Immunohistochemistry

Immunohistochemistry (IHC) on tissue slides is used in tumor differential diagnosis by targeting the reactivity of tumor antigens to specific antibodies (280). A wide range of IHC markers has been used in diagnosing epithelial (281), endothelial (282), and mesothelioma tumors (283), lung cancer (284), and lymphomas (285). Negative immunostaining may result from a lack of cross-reactivity of antibodies used in the dromedary or from prolonged impregnation in formalin (116). False positive reactions in IHC may occur with certain antibodies such as CEA (286). Usually, IHC tumor markers are used as panels to confirm diagnoses. Some of these are detailed below:

#### 14.4.1 Epithelial tumors

Cytokeratin, found in all epithelial cells, is essential for the diagnosis and classification of epithelial tumors (281). There are more than 20 isotypes of cytokeratin, categorized into either basic (1–8) or acidic (9–19, 161) groups, and they are extensively used in tumor diagnosis in camels (287, 288). These include Pancytokeratin (248), Cytokeratin AE1/AE3 (130), cytokeratin 5/6 (10, 210), cytokeratin-7 (251), and cytokeratin 20 (257). Other markers include epithelial membrane antigen (EMA; MUC1, PEM), a glycoprotein produced by various epithelial cells and widely used as a tumor marker for adenocarcinoma (289). However, some tumors, such as hepatocellular carcinoma, adrenal carcinoma, and embryonal carcinomas, are consistently EMA negative. Carcinoembryonic antigen (CEA), a glycoprotein that is very low in normal adult tissues, identifies cells expressing glycoproteins in some benign and malignant tumors, especially adenocarcinomas (286). Both EMA and CEA are diffusely expressed in the cytoplasm of adenocarcinomas in the dromedary (10, 161).

#### 14.4.2 Mesenchymal tumor markers

S100 proteins are expressed in normal tissues and in tumors arising from mesenchymal cells, including lipocytes, chondrocytes, melanocytes, neurons, glial cells, Schwann cells, dendritic cells, and salivary glands (290). S-100 proteins showed a positive reaction for malignant schwannomas and for pleomorphic adenomas of sweat glands in the dromedary (167, 265). Vimentin is another major protein expressed in cells of mesenchymal origin (291). Though relatively non-specific, vimentin helps in identifying the mesenchymal origin of some tumors and is usually negative for carcinomas (292). In the dromedary, vimentin was positively reactive for chondrosarcoma (180), primitive neuroectodermal tumor (166), sex cord-stromal tumors (11), myxosarcoma (257), and the stroma of tumors (205). The α-smooth muscle actin (α-SMA) is expressed in myofibroblasts and in cancers associated with stromal fibroblasts, such as breast cancer (293). In the one-humped camel, the marker was positive for leiomyoma and leiomyosarcoma (257). Non-specific esterase stain (NSE) is a non-specific neuroendocrine carcinoma marker and shows expression in other tumors, such as non-small-cell lung carcinomas and clear cell renal cell carcinomas (294, 295). Glial fibrillary acidic protein (GFAP) is a diagnostic marker for astrocytoma (296). Both NSE and GFAP markers were variably positive for primitive neuroectodermal tumor (166), but GFAP was negative in choroid plexus papilloma (251) in the dromedary.

#### 14.4.3 Mesothelial tumor markers

Mesothelioma originates from cells lining the pleura, pericardium, and peritoneum (297), with epithelioid, sarcomatoid, and biphasic mesothelioma subtypes. Pericardial mesothelioma was reported in an Arabian camel (298). Calretinin, CK5/6, WT1, D2–40, mesothelin, and EMA are the major IHC markers for the diagnosis of mesothelioma and differentiation of its subtypes (299, 300). GATA3 is a multispecific marker for mammary, urothelial, renal, germ cell tumors, epithelioid mesotheliomas, and paragangliomas (301). Other mesothelial markers have been identified (283).

#### 14.4.4 Round cell tumor markers

Round cell tumors are characterized by round cells with an amplified nuclear/cytoplasmic ratio and include peripheral neuroectodermal tumor, rhabdomyosarcoma, synovial sarcoma, chondrosarcoma, non-Hodgkin's lymphoma, neuroblastoma, hepatoblastoma, Wilms' tumor, desmoplastic small round cell tumor (302), and mastocytoma (191), all of which have been reported in the dromedary. The IHC markers for round cell tumors include S-100, desmin, and cytokeratin (303). Desmin stained positive for rhabdomyosarcoma (182), and S-100 was positive for multicentric schwannoma (167) and chondrosarcoma (180) in the dromedary.

#### 14.4.5 Germ cell tumor markers

OCT3/4 and D2-40 are IHC markers for classical seminoma, while CD30 is associated with embryonal carcinoma, with negative reactions in the rest of the germ cell tumors (304). The germ cell tumors reported in the dromedary included seminoma in the testicles (154, 211, 212) and dysgerminoma in the ovary (216) of dromedary camels, along with dermoid cysts; however, IHC markers were not utilized for the diagnosis of the above cases.

#### 14.4.6 Sex cord-stromal tumors

Inhibin and calretinin are the best IHC panel markers for sex cord-stromal tumors, though other markers could be useful but are of less sensitivity and specificity (305, 306). Inhibin alpha, an ovarian and testicular hormone, is a tumor marker for adrenocortical and sex cord-stromal tumors of the testes and ovaries (307, 308). In the dromedary, Inhibin alpha was positive in Sertoli and Leydig cells, as well as in the ovarian Sertoli–Leydig cell tumor (7), and was focally positive in granulosa-theca cells (11) but negative in steroid cell tumor nos (11). Melan-A is expressed in all melanomas, including ovarian steroid cell tumors and sex cord-stromal tumors (305). Melan-A was positively expressed in steroid cell tumor nos of the ovaries in the dromedary (11). Vimentin (V9) was positive for sex cord-stromal tumors (11).

#### 14.4.7 Lymphomas, leukemia and hemangiosarcoma

CD3 is commonly used in the diagnosis of T-lymphomas (137). Other T-cell markers include CD2, CD5, and CD7 (309). On the other hand, PAX-5, BLA36, CD15, CD20, CD30, and CD79α are used in the diagnosis of B-cell lymphoma (165, 310, 311). Only T-lymphomas were diagnosed in the dromedary (137). Hemangiosarcoma in the dromedary showed a positive reaction to the monoclonal CD31 antibody (151). Acute leukemia was reported in the dromedary (137, 138, 198, 199). However, IHC alone may have limited use for the diagnosis of leukemia; flow cytometry and genetic studies are required (312).

### 14.5 Genetic tools for cancer diagnosis

Inherited or acquired changes in proto-oncogenes, tumor suppressor genes (TP5), and/or DNA repair genes (BRCA1 and BRCA2) are implicated in cancer development (313). Cancer diagnosis through genetic tools is primarily used in humans and is relatively new in the veterinary field. Whole genome or targeted gene sequencing panels to detect mutations, gene fusions, and variations in copy number can be quickly performed using next-generation sequencing (NGS) (314). Specific gene mutations, such as those in BRCA1/2 and EGFR, can be detected using PCR-based methods for mutation detection (315). Chromosomal abnormalities in human and animal cancer cells can be identified through fluorescence *in situ* hybridization (FISH) or comparative genomic hybridization (314). Cancer classification can be achieved through microarray analysis, where the expression of a large number of genes is assessed. These techniques will be very helpful for diagnosing genetic diseases in camel breeding centers and for breeder decisions regarding selection or elimination.

### 14.6 Molecular biology tools for cancer diagnosis

Molecular biology tools for cancer diagnosis and research include IHC, flow cytometry, metabolomics, and proteomics studies.

#### 14.6.1 Flow cytometry

Flow cytometry has been applied in veterinary oncology using panels of fluorescently labeled antibodies to measure proteins expressed by cells. It checks for the presence of cancer antigens, especially in leukemia and lymphoma, in blood or tissues through the immunophenotyping technique. Flow cytometry also determines the DNA content of cells (316).

#### 14.6.2 Metabolomics and proteomics studies

Metabolomics utilizes machine learning *(ML)*, a branch of AI, focusing on the study of small molecules (metabolites) in biological systems. The main metabolomics techniques include nuclear magnetic resonance spectroscopy (NMR), which provides detailed metabolite structures (317), and mass spectrometry (MS), which identifies metabolites (318). MS is typically coupled with separation techniques such as gas chromatography–mass spectrometry (GC–MS), liquid chromatography–mass spectrometry (LC–MS), and capillary electrophoresis–mass spectrometry (CE–MS). These techniques identify and quantify metabolites in biological systems. Metabolomics investigates cancer metabolism (319), analyzes metabolic pathways, and helps in cancer biomarker discovery (320). On the other hand, proteomics utilizes mass spectrometry and gel electrophoresis to study proteins, including their identification, interactions, and post-translational modifications in biological systems (321). In proteomics, techniques such as tandem mass tags allow for the simultaneous analysis of a large number of proteins from biological fluids, cells, or tissues, fostering a comprehensive understanding of cancer mechanisms, including metastasis, angiogenesis, proliferation, and resistance. Proteomic studies are also utilized for biomarker discovery (321, 322). These techniques can revolutionize tumor diagnosis in camels.

### 14.7 Virological techniques for diagnosis of oncogenic viruses

Oncogenic viruses include, but are not limited to, human papillomavirus (HPV) types, hepatitis C virus (HCV), hepatitis B virus (HBV), human T-cell lymphotropic virus type 1 (HTLV-1), Epstein–Barr virus (EBV), Kaposi sarcoma-associated herpesvirus (KSHV), and HIV-1 (323). The oncogenic viruses affecting pet animals include feline leukemia virus, feline immunodeficiency virus, papillomavirus, gamma herpesvirus, hepadnavirus, and mouse mammary tumor virus (324). Ruminants are also infected by oncogenic viruses such as bovine papilloma viruses (BPVs) (17–21, 131–134), bovine leukemia virus (BLV) (325), and Jaagsiekte sheep retrovirus (326). Interspecies jumping and recombination of viruses have been reported (142, 327). Numerous current virological techniques are available to diagnose oncogenic viruses in humans and animals, including virus detection, gene expression analysis, or integration into the host genome. These techniques include:

#### 14.7.1 Molecular techniques

(a) Polymerase chain reaction (PCR) and quantitative PCR (qPCR) are used to detect and quantify viral DNA/RNA in viruses such as HPV and HBV. (b) Reverse transcription PCR (RT-PCR) detects RNA in RNA viruses such as HCV. (c) Digital droplet PCR (ddPCR) quantifies nucleic acids in latent EBV or HTLV-1 with low viral loads (328).

#### 14.7.2 *In situ* hybridization (ISH)

Chromogenic ISH (CISH) and fluorescent ISH (FISH) help determine the presence of a virus in tumor cells. These techniques detect viral DNA/RNA, such as HPV E6/E7 mRNA in cervical biopsy sections and EBV-encoded small RNAs in nasopharyngeal carcinoma (329).

#### 14.7.3 Immunohistochemistry (IHC)

This technique detects viral antigens such as HPV E6/E7 proteins, EBV latent membrane protein (LMP1), and HBV (HBx) protein in tissue samples. The IHC technique was successfully applied in the investigation and diagnosis of papillomaviruses in dromedary camels using papillomavirus antibody or bovine papilloma 1 antibodies (128, 130).

#### 14.7.4 Next-generation sequencing (NGS)

This technique is primarily used to discover novel oncogenic viruses by performing comprehensive analyses of viral genomes, viral integration site analysis, and host–viral interactions (314).

#### 14.7.5 Serological tests

These are screening tests performed to detect antigen/antibody reactions. They are used to screen for oncogenic viruses such as HBV, HCV, and EBV (330) alongside other tests.

#### 14.7.6 Viral load testing

This test is based on qPCR or ddPCR techniques targeting RNA/DNA for monitoring HCV, HBV, EBV infections, and their association with cancer (324).

#### 14.7.7 Southern blot/Hybrid capture

This is an older technique used to detect HPV DNA and viral integration. A simplified hybrid capture approach has recently been applied (331).

### 14.8 Serum tumor markers

Serum tumor marker panels are useful as screening tests for tumor diagnosis, follow-up, and prognosis in human subjects but are not commonly used in the veterinary field. However, follow-up of valuable dromedary breeds after tumor surgery may be worth a trial. High serum levels of tumor markers need confirmation by histopathology. The serum tumor markers may include, but are not limited to: NSE for small-cell carcinoma of the bronchus, neuroblastoma, squamous cell carcinoma of the cervix and lungs; alpha-fetoprotein (AFP) for primary liver carcinoma and germ cell tumors; lactate dehydrogenase for germ cell tumors (332); CA 125 for ovarian carcinoma (333); CA72-4 for stomach carcinoma (334); CEA for breast, lung, pancreas, stomach, colon, and rectal carcinoma (286). EMA and its epitopes CA 27.29 and CA 15-3 could be used for breast cancer (335). Serum S100 is used for metastasis in uveal melanoma (336). Inhibin A is for granulosa cell tumors, and hormonal tumor markers such as anti-Müllerian hormone are for granulosa cell tumors (337). Calcitonin is for parafollicular cells and medullary thyroid carcinoma (338), while thyroglobulin is for papillary thyroid carcinoma (339). B-HCG is used to rule out pregnancy and adnexal masses (cervix, ovaries, uterus, and fallopian tubes). HCG is used to differentiate seminomas from non-seminomas in testicles, teratomas of non-testicular origin, and germ cell tumors in the ovary (340).

### 14.9 Future directions in cancer diagnosis and research in the dromedary camel

Future directions for tumor diagnosis and treatment in the dromedary camel include the introduction and utilization of new and advanced techniques for human cancer diagnosis, such as AI-integrated molecular profiling, nanopore sequencing, and digital pathology aided by AI, among other techniques. AI-integrated molecular profiling integrates AI, machine learning, deep learning, genomics, proteomics, and imaging to disclose alterations and mutations of oncogenes in cancers of unknown primary origin and to predict diagnosis and treatment (341). The camel breeding centers will benefit from this technique to enhance genomic selection to optimize traits and to avoid those with inherited cancer genes. Additionally, those working in research and tumor diagnosis in camels will benefit from Nanopore sequencing technology, as it detects the 14 hallmarks acquired by cancer during its progression to evade, alter, and bypass cell regulatory mechanisms. This tool is used to sequence tumor DNA and RNA for the detection of genome, transcriptome, and epigenetic alterations (342). Digital Pathology aided by AI could be used to analyze molecular pattern information generated from digital camel biopsy slides (343). Other future technologies that could benefit camel oncology researchers and diagnosticians include Liquid Biopsy, which screens blood for early detection of circulating tumor DNA (ctDNA) and genetic alterations to aid in diagnosing non-invasive cancer (344). Moreover, single-cell genomic analysis detects genetic alterations within individual cells and could find future applications in camel neoplastic diagnosis and research. Furthermore, AI-driven genomic interpretation can forecast cancer risk in highly valuable camel breeds, allowing for the choice of appropriate future therapies from a large human genetic database (345). CRISPR-based diagnostic tools that utilize CRISPR RNAs (crRNAs) and Cas enzymes within the CRISPR-Cas system (346) could also be utilized in camel cancers.

### 14.10 Current and future cancer treatment in camels

The current cancer therapy in the dromedary camel is based on surgical excision, mainly for skin tumors, cryosurgery, hyperthermia, or combinations of these, depending on tumor type, with variable success. However, chemotherapy, radiotherapy, and immunotherapy used for horses and pet animals (347, 348) could find application in individual camels in the future. There is comprehensive research on nanoparticle synthesis from medicinal plants and their utilization in cancer research and drug discovery (349, 350). The camel will definitely benefit from these future advances in cancer treatment. Additionally, the camel will benefit from future innovative cancer treatments utilizing molecular medicine, where targeted therapy is used to block cancer cell growth by direct interference with specific targeted molecules required for carcinogenesis (351). Furthermore, therapies such as precision medicine will be extensively used in individual cancer treatments in camels, utilizing genomic information for therapeutic guidance (352). The treatment of camel cancers will undoubtedly benefit from future advancements in cancer therapy.

## 15 Conclusion

Different tumor types have been reported in the dromedary camel. These include, but are not limited to, carcinomas, fibromas, teratomas, lymphomas, leukemias, papillomas, and sarcomas. Large skin and toenail tumors were comparatively high, affecting locomotion, racing, and market value. Reproductive neoplasia was not high, except for ovarian tumors. The major tumor risk factors in the camel environment include contamination of feed by various fungal toxins and contamination of grazing areas, water, and soil with pesticides, herbicides, petroleum oil, and heavy metals. Tumor cases are expected to increase with the recent advances in camel farming, surveillance, and reporting systems. Our findings revealed significant gaps, as most tumors diagnosed in dromedaries originated from biased abattoir surveys, primarily involving barren females or young males. The review emphasized the need for further genetic and molecular biology research, alongside clinical diagnosis, reporting, surveillance, risk factors, epidemiology, and treatment of tumors in the dromedary camel. Moreover, research on oncogenic viruses, such as those involved in papillomas, leukemias, and viruses related to zoonotic diseases, is required using current and future molecular techniques such as ISH, IHC, new screening serological tests, viral load testing, and newly modified hybrid capture. AI-based future applications will revolutionize tumor diagnosis and treatment, as well as animal selection for breeding based on genetic analysis and oncogenic gene amendment or elimination.
