# Supplementary material for: Neoplasia in the dromedary camel: a review (Camelus dromedarius)
Source: Front Vet Sci. 2025 Dec 10;12:1664874. doi: 10.3389/fvets.2025.1664874 (PMC12731250; doi:10.3389/fvets.2025.1664874)
Supplement: Supplementary file 1 [file Data_Sheet_1.docx]

Table S1: Summary of tumor risk factors in the dromedary camel: Risk factor, causative agent, public health concerns/exposure route, mechanism of carcinogenicity, tumor typed induced by risk factor, references

| **Risk factor** | **Causative agent of cancer** | **Exposure route & Public concern:** | **Carcinogenesis** | **Tumor type(s)** | **References** |
| --- | --- | --- | --- | --- | --- |
| Mycotoxins in camel's feed | -Aflatoxin B 1, M1  -Ochratoxin A,  -Zearalenone,  -others. | Food chain (Milk, meat, livers, kidney, brain). Zearalenone increases estrogenic activity. | permanent cell/DNA damage or mutations, inhibit protein synthesis, | Carcinomas of Liver, lungs, renal cells, gall bladder, reproductive organs etc. | **26** Stewart (2019);  **32** Almoammar et al (2014);  **39**Yosef et al., (2014) |
| Agricultural pesticides herbicides & fungicides | -Organochlorines (OCs)  -Organophosphate (OP)  -Carbamates (CMs), | Carcinogenic pesticides/herbicides residues in food chain, air, water, mother’s milk. Hormonal related cancers by some OP | -OCs: Epigenetic modulation with decreased intercellular communication.  - CMs: interact with BRCA1 gene and interfere with the key pathways.  -OP: epigenetic modulation, DNA damage.  - OCs, OP, CMs: induce brain cell oxidative stress via transcription factor JAK-STAT and Keap1/Nrf2/ARE, damage of nucleic acids, lipids and proteins. | Liver, Lung, breast, cervical, testicular & pancreatic carcinomas, other hormonal related cancers, soft-tissue sarcoma, non-Hodgkin lymphoma, leukemia, brain gliomas & meningioma | **26** Stewart (2019)  **43** Sallam et al (2008);  **44** Alavanja and Bonner (2012) |
| Pharmaceutical drugs (many and only few examples are included ) | -Chloramphenicol (CPL),  -Hexachloroethane (HCE),  -Phenylbutazone (Bute)  -Barbiturates (Bars)  -Cyclosporine (CsA),  -Carbaryl (INN)  -Synthetic Pyrethroids (SPs) | -Chemical residues in food chain  -Drug resistance | -CPL: inhibits protein synthesis in cells, induces abnormal differentiation (very large  Cell size cells with multiploid nuclei).  -HCE: sister chromatid exchange without chromosomal damage  -Bute: Lymphoproliferative malignancies  -Bars: stimulates cell, proliferation, increases DNA synthesis, interferes with DNA repair, induces higher nuclear antigen activity.  -CsA: suppresses immunity, inhibits DNA repair, anti-apoptotic effect  INN: Induces cytochrome P450 1A gene expression  --SPs: Genomic rearrangements, DNA modification, alteration in genes expression | -CPL: Leukemia,  -Bars: liver and lung Carcinomas, Sarcomas, brain tumors  - Bute: Acute Leukemia  -CsA: Squamous & Basal cell Carcinomas, Kaposi Sarcoma, non-Hodgkin’s Lymphoma  - INN: Hemangioma and Hemangiosarcoma, urinary bladder Transitional-cell Carcinoma and Papilloma, renal tubular cell-Adenoma, Thyroid follicular-cell Adenoma, hepatocellular Carcinoma, vascular tumors | **26** Stewart (2019)  **52** Belitskiy et al (2020);  **53** Woodward (1992) |
| Hormones: | -estrogens (E)  -progesterone (P)  -androgenic steroids (AAS),  -glucocorticoid (GC) | E & P: concern of cancer via IVF.  AAS: Concerns about hormonal imbalance and sexual behavioral changes.  GCC: concern about suppressed immunity | -E binds ER on cancers cells to stimulate their growth and division. E alters DNA repair.  -P conceals tumor’s signals from immune system.  - AAS: Induces cell proliferation, enhances DNA transcription via androgen and/or estrogen receptors or activation of transcription factor pathways in Leydig cell tumor.  -GC: immunosuppression | E & P: breast, endometrial, ovarian, cervical, prostate, liver, lungs carcinomas  AAS: breast & prostate cancer  GC (Long-term use): Lung & liver cancer | **26** Stewart (2019)  **70** Farhud et al 2019;  **74** Huo et al 2017;  **79** Oh & Song 2020 |
| Carcinogenic Plants (many but exemplified by few) | Aristolochic acid, Aristolochine, Phorbol esters, Anthaquinone, ricin Pyrolizidine alkaloids, tannins, Coumarin, Cycasin, Phytoestrogen, Ptaquiloside, macrozamin. | Carcinogenicity, teratogenicity, mutagenicity, endocrine disrupters, reproductive effects) | DNA damage, cell mutation, activation of oncogenes | Carcinomas: Liver, lungs, reproductive system, oral cavity, stomach | **26** Stewart (2019)  **83** Bode & Dong (2015),  **88**  Manzo et al, (2019);  **94** Abderrahman et al (2018);  **96** Tan et al., (2020) |
| Gold mining | Heavy metals (As, Cd, Ni, Cr) sulphate ions. toxic industrial gases | Heavy metal Contamination to ground/surface water rivers, soil, crops and pasture. Acidic waste contamination. Emission of CO_2_ and industrial dust (ozone destruction & global warming) | Oxidative stress of endoplasmic reticulum and mitochondria. DNA damage (Cr, As, Cr). DNA repair inhibition (Cd, Nr, As). Genomic instability (Cr, As, Cd, Cr). Gene amplification, epigenetic mutation (As). Aneuploidy (Cr, As). Chromosome aberrations (Nr). Cell transformation (Cr). Disturbance in tumor suppressor protein (Cd). | Wide range of cancers mostly carcinoma of lung. prostate urethra, bladder, skin, kidney, liver  . | **26** Stewart (2019)  **48** Soleimani et al 2021,  **107** Csavina et al 2012;  **109** Kim et al 2015;  **112** Asli et al 2019 |
| Petroleum and byproducts | Benzene, acetaldehyde, benzo(a) pyrene, 1,3- butadene.  Plastic waste | - Air pollution.  -Land, water, soil, agriculture & pasture pollution  -Waste Polyethylene bags hazards | The carcinogenic petrochemical enhance chromosome mutation, cause alteration in cell structure, function and metabolism | Lung Mesothelioma, Leukemia, Lymphomas, skin Melanoma, Multiple Myeloma, urinary bladder Carcinoma | **26** Stewart (2019)  **24** Jadallah et al., 2021,  **123** Zylstra 2012;  **124** Wernery et al, 2021 |
| Viral infections | -papilloma virus strains (CdPV1, CdPV2),  -others but not proven yet (EBL, JSRV, HEV) | -Contamination of food chain by HEV from camel meat.  -Trans-species jump of viruses | Instability/mis-segregation of chromosomes during mitosis causing aneuploidy (imbalance in chromosome number) and genetic alterations | Papillomatosis, Fibropapilloma, corneal Papilloma, epithelial Papilloma, camel Lymphoblastic Leukemia (viral origin not proved) | **26** Stewart (2019)  **21** Ure et al; 2011;  **130** Kiliç et al, 2010; **140**  El-Kafrawy et al., 2020 |
| Long exposure to Sunlight | Ultraviolet radiation (UV) | White coated, skin devoid of hair.  Ozone depletion | damage DNA in cutaneous epithelium | Squamous & Basal cell Carcinoma, Hemangiosarcoma, dermal Hemangioma other cutaneous neoplasia | **26** Stewart (2019)  **6** Al-Sobayil & El-Amer (2013);  **146** Saucedo et al., (2023)  **149** Hiatt & Beyeler, (2020) |
| Inherited cancer genes | Inherited gene mutations i.e., BRCA1, BRCA2, PTEN WT1, WTX, CTNNB1, TP53, APC, DICER1 | Inheritance of genetic diseases and transmission to off-spring | Inherited defective genes e.g PTEN, WT1 WTX,TP53, CTNNB1, DICER1 etc., | Hamartoma,  Nephroblastoma, , Arrhenoblastoma, Primitive Neuroectodermal tumor, Teratomas | **5** Gao et al, 2020;  **7** Ali et al, 2019  **26** Stewart (2019) |
| Old age | Built-up of genetic changes in cell DNA by time | Old age along with inherited cancer genes possibly have an additive effect | Increased proteins synthesis sustaining cell growth, lower proteins synthesis that check cell growth or stop proteins synthesis involved in cell apoptosis | Different types including skin, liver lungs Carcinomas | **9** Siddiqui, & Telfah (2010);  **26** Stewart (2019)  **130** Kiliç et al 2010 |

Table S2: Summary of tumors reported in the digestive system of the dromedary camel: tumor classification, tumor benign or malignant and differentiation, No. of cases diagnosed, sex and age, organs involved, lesion size and distribution, references and reference number. Asterisks (***** - ********) explained below

| **Tumor Classification** | ***Tumor**  **-**Type: B; M  -Differentiation *wd, pd, an* | **No of**  **cases** | ****** **Sex:** *m, f*  **Age:** *y* | *****Organs**  **involved** (Prim, Invas, Metast) | ******Lesion**  **-Size:** *L, Med, S, U*  **-Distribution:**  *1, 2, 3, 4, 5* | **References** |
| --- | --- | --- | --- | --- | --- | --- |
| Fibro-adenocarcino -sarcoma | 1: M, *md*  2: M, *an* | 1 | *f*, 8 y | 1: Left lower gum (*Inv*)  2:mandibule, molar tooth (*Inv*) | *L, 4*  *L, 4* | **162** Ramadan et al (2001) |
| Fibromyxosarcomas | M, *wd* | 2 | *f* | lobulated gum, tongue, cheek, lips | *S-Med, 4* | **6** Al-Sobayil and El-Amir (2013) |
| Hepatocellular carcinoma | M, *wd* | 1 | **-** | Liver | *U, 3* | **155** El Miniawy et al (2013) |
| squamous cell carcinoma | M, *wd* | 1 | *f, 12y* | Swelling in right cheek wall mucosa | *L, 1* | **158** Jena et al., (2015) |
| Squamous cell carcinoma | M, *wd* | 1  1 | *f, adult*  *m, adult* | gum  dulaa | *L, 1*  *L, 1* | **6** Al-Sobayil and El-Amir (2013) |
| Cholangiocarcinoma  Cholangiocarcinoma  Cholangiocarcinoma | M, *wd*  M, *wd*  M, *wd* | 2  1  2 | *M, f, adult*  *f, adult*  *m, f, adult* | Hepato-biliary (Invas)  Hepatobiliary epith,  Hepatobiliary | *S- Med- L, 3*  *U, 5* | **155** El Miniawy et al (2013)  156 Al- Hizab (2009)  **157** El-Mahdy et al (2013) |
| Seminoma and  cholangiocarcinoma | M  M | 1 | *m*, 18 y | 1: right testicle *(Seminoma)*;  2: liver cholangio-carcinoma (invation)  3: 2: capillaries (inva) | *L, 1*  *Med, 3* | **154** Birincioglu, et al., (2008) |
| Lymphosarcoma | M, *wd* | 1 | *adult* | Liver, | *U, 3* | **157** El-Mahdy et al (2013) |
| Metastatic vaginal adenocarcinoma | M, *wd* | 1 | *f*, adult | Vagina (primary), liver, mesentery iliac lymph node, | *S, 4* | **161** Ali et al., (2018) |
| Invasive adenocarcinoma | M, *wd,* | 1 | *f*, 15 y | Omasum/  Abomasum; *invas* | *L, 2* | **159** Tharwat et al., 2018 |
| Cholangiocarcinoma-Leiomyosarcoma | M, *wd, pd*  M | 1 | *m*, 15 y | liver | *S-Med , 3* | **151** Elmaghraby et al (2023 |
| Hemangiosarcoma-Cholangiocarcinoma-Leiomyoma | M  M, *pd*  B | 1 | *m*, 9 y | Liver, *inv* | *S, 3* | **151** Elmaghraby et al (2023 |
| Primitive Neuroectodermal tumor | M, an | **1** | *m*, 9 y | -3^rd^ lumber vertebra,  -liver | *U*, 4 | **166** Weiss, Walz, (2009) |
| Multicentric T-cell Lymphoma | M | 1 | *f*, 7 y | Liver, small intestine, kidneys, mesenteric and other lymph nodes, | *L- Med, S, 4* | **163** Simmons et al (2005) |
| T-cell lymphoma | M, | 1 | *m*, 10 y | multiple nodules in liver & spleen enlarged lymph nodes in abdomen & thorax | *S-Med, 4* | **164** Raval et al., 2016, |
| Disseminated gamma‐delta T‐cell lymphoma | M | 1 | *f*, 12 y | Right eye, liver, mesenteric lymph nodes | *L,4* | **165** Ibrahim et al., (2023) |
| Lymphoma | M | 1 | *m,* 6 y | Liver, prim, *inv* | *S, 3* | **40** Elmaghraby et al (2023 |
| Myelolipoma-osseous metaplasia | B | 1 | *m*, 10 y | liver | *S, 1* | **151** Elmaghraby et al (2023 |
| Multicentric fibro-myxoid peripheral nerve sheeth tumor (Multicentric schwannoma*)* | B | 1 | *m*, 4 y | Serosa of:  1: for-stomach  2: L intestine  3: Mesentery  4: Liver  5: spleen  (1-5= Inva) | *L, Med, S, 4* | **167** Khodakaram-Tafti & Khardadmehr, (2010) |
| Fibrous Epulis | B | 1 | *m*, 2 y | Labial upper gum | *S, 1* | **173** Ramadan & El Hassan (1980) |
| Fibroma | B | 1 | *f*, 5 y | Rectum, | *L, 1* | **172** Jhirwal et al ( 2004) |
| Fibrma | B | 1 | *m*, adult | Soft palate | *L, 1* | **171** Barvalia et al, 1998 |
| *Cavernous hemangioma*  *Cavernous hemangioma Cavernous hemangioma* | B  B  B | 1  1  2 | *f, m*, adult  *f, m*, adult  *f, m*, adult | *Liver,*  *Liver,*  *Liver,* | *S, 1*  *S, 1*  *U, 1* | **175** El-Mahdy et al (1997)  **157** El-Mahdy et al (2013)  **41** El Miniawy et al (2013) |
| *Cavernous hemangioma* | B | 1 | Not specified | liver | *L , 1* | **113** Rezaie et al (2015) |
| *Leiomyoma* | B | 1 | adult | liver | *U, 1* | **155** El Miniawy et al (2013) |
| *leiomyoma* | B | 1 | Not specified | Liver | *S, depressed, 1* | **113** Rezaie et al (2015) |
| *Leiomyoma* | B | 3 | *m*, 9-11y | Liver, | *U, 3* | **155** Elmaghraby et al (2023) |
| Abdominal leiomyoma | B | 1 | *m*, 2 *y* | left kidney, spleen left abdominal wall, blood vessels | *Very L, 4* | **169** Sadan et al (2024) |
| *lipoma* | B | 3 | *f, m*, adult | Liver | *S,Med, 3* | **113** Rezaie et al (2015) |
| *Lipoma* | B | 1 | *m*, 7y | left ischiorectal fossa | *L, 1* | **174** Kaswan et al., (2013) |

***Tumor:** B: Benign; M: Malignant.

**Differentiation:** *wd=* well differentiated/moderately differentiated; *pd* = poorly differentiated; *an*= anaplastic**.**

****** **Sex:** *m=male, f=female;* **Age:** y= years

***** Organs involved** (name of organ): Prim = Primary lesion; Inv = Invasive lesion; Meta= metastatic lesion

******Lesion** **size**: *L* = Large; *med* =Median; *S* =Small, *U*= Undefined

**Lesion Distribution**: *1*= single lesion in single organ; *2* = single lesion in multiple organs; *3* = multiple lesions in single organ; *4* = multiple lesions in multiple organs, *5*= infiltrative tumor cells

***** Organs involved** (name of organ): Prim = Primary lesion; Inv = Invasive lesion; Meta= metastatic lesion

Table S3: Summary of tumors reported in the respiratory system of the dromedary camel: tumor classification, tumor benign or malignant and differentiation, No. of cases diagnosed, sex and age, organs involved, lesion size and distribution, references and reference number. Asterisks (***** - ********) explained below

| **Tumor Classification** | ***Tumor**  **-**Type**: B; M**  -Differentiation *wd, pd, an* | **No of**  **cases** | ****Sex:** *m, f*  **Age:** *y* | ******* **Organs**  **involved** (Prim, Invas, Metast) | ******Lesion**  **-Size:** *L, Med, S, U*  **-Distribution:** *1, 2, 3, 4, 5* | **References** |
| --- | --- | --- | --- | --- | --- | --- |
| Primary Brochiolo-alveolar adenocarcinoma | M, *an* | 1 | *f*, 12 y | Both Lungs, lymph node, lymh vessels; | *L- Med- S; 4* | **176** Gameel et al (1998) |
| Brochiolo-alveolar adenocarcinoma | M | 1 | Not specified | Lungs | *U* | **177** Taha et al (2007) |
| pulmonary papillary carcinoma | M*, wd* | 4 | *f*,15 y | Masses in both lungs | *L-, Med- S, 3* | **115** Muna et al., (2018) |
| Mammary carcinoma (metastasis to lungs) | M, *an* | 1 | *f*, 15 y | Mammary (Prim). Lungs, its micro vasculature pulmonary Lymph nodes, *metast*. | *L, 4* | **178** Bryant, et al (2007) |
| Osteosarcoma (metastasis to lungs) | M, *wd* | 1 | *f*, 7 y | Bone (right tarsal joint, tarsal and metatarsal bones); both lungs | *L, Med, S, 4* | **179** Tuttle et al (2007) |
| Chondrosarcoma  (metastasis to lungs) | M, *wd* | 1 | *f*,≈5y | carpal joint, carpal and metcarpal bones; Metast to Both lungs. | *L- Med- S,*  *4* | **180**  Janardhan, et al (2011) |
| Disseminated sarcoma | M | 1 | *f*, adult | Both lungs, wound in muscles above tarsus. Metast. | *L, Med, S,*  *4* | **181**  Bjorklund (2014) |
| Rhabdomyosarcoma | M*, pd* | 1 | *f, 9 y* | pedunculated cauliflower like growth at nasal orifice obstructing right nostril | *L, 1* | **182** Zakia-Mohammed, et al., (2007) |
| Pulmonary lymphosarcoma | M | 1 | *f*,16 y | Both lungs. | *L, 2* | **183**  Wernery, Kumar (1996) |
| T-cell Lymphoma | M | 10 | *f*, > 8 y | Blood, lungs, spleen and lymph nodes. | *U, 4* | **137** Wernery & Kaaden, (1995) |
| Multiple pulmonary leiomyoma/multiple Leiomyomatous hamartomas | B | 1 | *f* **, -** | Lungs, Metast/uterus | *U, 2* | **184** Burkhardt, et al (1981) |
| Pulmonary Leiomyoma | B | 2 | *m*, 8 y  *m*, 10 y | Lung pleura mass  Lung tissue mass | *U, 1*  *U, 1* | **185** Wareth and Moustafa (2013) |
| Pyogranuloma/ lobular capillary hemangioma | B | 1 | *adult* | overgrowth in Retropharyngeal region | *U, 1* | **186** El-Shafaey et al (2020) |

***Tumor: B**: Benign; **M**: Malignant

**Differentiation:** *wd=* well differentiated/moderately differentiated; *pd* = poorly differentiated; *an*= anaplastic**.**

****** **Sex:** *m=male, f=female;* **Age:** y= years

***** Organs involved** (name of organ): Prim = Primary lesion; Inva = Invasive lesion; Meta= metastatic lesion

******Lesion** **size**: *L* = Large; *Med* =Median; *S* =Small*. U*= Undefined

**Lesion Distribution**: *1*= single lesion in single organ; *2* = single lesion in multiple organs; *3* = multiple lesions in single organ; *4* = multiple lesions in multiple organs, *5*= infiltrative tumor cells

Table S4: Summary of lymphomas and leukemia reported in the dromedary camel: Tumor classification, tumor benign or malignant and differentiation, No of cases diagnosed, sex and age, organs involved, lesion size and distribution, references and reference number. Asterisks (***** - ********) explained below

| **Tumor Classification** | ***Tumor**  **-**Type: B; M  -Differentiation *wd, pd, an* | **No of**  **cases** | ****-Sex:**  *m, f*  **-Age:** *y* | *****Organs involved** (Prim, Invas, Metast) | ******Lesion**  **-Size:** *L, Med, S, U*  **-Distribution**  *1, 2, 3, 4, 5* | **References** |
| --- | --- | --- | --- | --- | --- | --- |
| *Lymphosarcoma* | M | 1 |  |  | *U* | **190**Youssef et al (1987)  **183** |
| *Pulmonary lymphosarcoma* | M | 1 | *f*, 16 *y* | Both lungs. | *L, 2, diffuse* | Wernery & Kumar (1996) |
| *Disseminated gamma‐delta T‐cell lymphoma* | M | 1 | *f*, 12 y | Right eye, liver, mesenteric lymph nodes | *L,4* | **165** Ibrahim et al., (2023) |
| *lymphosarcoma* | M | 1 | *Not specified* | neck region | *L, 1* | **186** El-Shafaey et al., (2020) |
| *Lymphosarcoma* | M, *wd* | 1 | - | Liver, | *U, 3* | **157** El-Mahdy et al., (2013) |
| *T-cell lymphoma* | M | 10 | *f*, > 8 y | Blood, lungs, spleen and lymph nodes. | *U, 4 highly infiltrative to organs* | **137** Wernery & Kaaden, (1995) |
| *Multicentric*  *T-cell lymphoma* | M | 1 | *f*, 7 *y* | mesenteric and lymph nodes, liver, both kidneys, small intestine | *L- Med- S, 4* | **163** Simmons et al (2005) |
| *Lymphatic lymphoma* | M | 1 | *Not specified* | *Not specified* | *U* | **191** Abubakr et al., (1998) |
| *Lymphatic leukemia* | M | 1 | *f*, 10 y | Peripheral blood *lymphoblasts* | *-* | **198** Tageldin et al (1994) |
| *Acute lymphoblastic leukemia* | M | 2 | Not specified | Peripheral blood *Lymphoblasts* | *-* | **138** Afzal and Hussain (l995) |
| *Lymphocytic leukemia* | M | 10 | *f,* *m*, >8y | Peripheral blood *Lymphoblasts* | *-* | **137** Wernery & Kaaden (1995) |
| *Lymphoid leukemia* | M | 10 | *Not specified* | Peripheral blood *lymphoblasts* | *-* | **12** Choudhry (2017) |
| *Mast cell tumor (mastocytoma)* | M | 1 | *Not specified* | Skin | *U* | **191** Abubakr et al., (1998) |
| *Cavernous hemangioma*    *Cavernous hemangioma*  *Cavernous hemangioma* | B  B  B | 1  1  2 |  | *Liver,*  *Liver,*  *Liver,* | *S, 1*  *S, 1*  *U, 1* | **175** El-Mahdy et al (1997)  **157** El-Mahdy et al (2013)  **155** El Miniawy et al (2013) |
| *Hemangioma* | B | 1 | *f*, 10 y | Cervical lymph node | *L, 1* | **201**Aljameel & Halima., (2015) |
| *Pyogranuloma/ lobular capillary hemangioma* | B | 1 | *adult* | Retropharyngeal  region | *L, 1* | **186** El-Shafaey et al (2020) |

***Tumor:**B: Benign; M: Malignant

**Differentiation:** *wd=* well differentiated/moderately differentiated; *pd* = poorly differentiated; *an*= anaplastic**.**

****** **Sex:** *m=male, f=female;* **Age:** y= years

***** Organs involved** (name of organ): Prim = Primary lesion; Inva = Invasive lesion; Meta= metastatic lesion

******Lesion** **size**: *L* = Large; *med* =Median; *S* =Small; *U*= Undefined

**Lesion Distribution**: *1*= single lesion in single organ; *2* = single lesion in multiple organs; *3* = multiple lesions in single organ; *4* = multiple lesions in multiple organs, *5*= infiltrative tumor cells

Note: B cell lymphoma not reported

Table S5: Summary of tumors reported in the musculoskeletal system of the dromedary camel: Tumor classification, tumor benign or malignant and differentiation, No of cases diagnosed, sex and age, organs involved, lesion size and distribution, references and reference number. Asterisks (***** - ********) explained below

| **Tumor Classification** | ***Tumor**  **-**Type: **B; M**  -Differentiation *wd, pd, an* | **No of**  **cases** | ****Sex:** *m, f*  **Age:** *y* | ******* **Organs**  **involved** (Prim, Invas, Metast) | ******Lesion**  **-Size:** *L, Med, S, U*  **-Distribution:** *1, 2, 3, 4, 5* | **References** |
| --- | --- | --- | --- | --- | --- | --- |
| *Intraosseous squamous cell carcinoma* | M*, wd* | 2 | *f, 6.5 y;*  *f, 9.5 y* | Right maxilla  Left maxilla | *L, 4*  *L, 4* | **165** Ibrahim et al., (2023) |
| *Squamous cell carcinomas* | M*, wd* | 1 | *adult* | Maxilla | *L, 1* | **186** El-Shafaey et al., (2020) |
| *Squamous cell carcinoma of skin* | M, *wd* | 1 | *m*, 7 y | Skin of right hock joint (prim); tarsal & metatarsal bones (inv) | *L, 2* | **202** Morales-Briceño., et al., (2017) |
| *Fibro-adenocarcino -sarcoma* | 1: M, *wd*  2: M, *an* | 1 | *f*, 8 y | 1: Left lower gum, salivary gland (*prim*)  2:mandibule, tooth: premolar (*Inv*) | *L, 4*  *U, 4* | **162**Ramadan et al., (2001) |
| *Chondrosarcoma* | M*, wd* | 1 | *f*, 4 *y and 10 months* | Carpal joint (prim), Carpal bone & proximal metcarpal bone (inves)  Lungs (Metas) | *L- S, 4* | **180** Janardhan et al., (2011) |
| *Chondrosarcoma* | M | 1 | *f*, 14 *y* | Solar aspect of left hind foot | *Med, 1* | **204** [Purohit](https://www.researchgate.net/scientific-contributions/NR-Purohit-2211064811) et al., (1994) |
| *Osteosarcoma* | M | 1 | *f*, 7 y, - | Left ulnar (prim), Lungs (metast) | *L ulnar, S lungs,*  *4* | **179** Tuttle et al., (2007) |
| *Osteosarcoma* | M | 1 | *Not Specified* | Ventral to mandible, | *Med-L, 1* | **186** El-Shafaey et al., (2020) |
| *Rhabdomyosarcoma* | M, *pd* | 1 | *f, 9 y* | Pedunculated cauliflower like growth at nasal orifice obstructing right nostril | *L, 1* | **182** Zakia-Mohammed, et al., (2007) |
| *Primitive Neuroectodermal tumor* | M, *an* | **1** | *m*, 9 y | -3^rd^lumber vertebra,  -liver | *U, 4* | **166** Weiss, Walz., (2009) |
| *Vertebral osteoma* | B | 1 | *f*, 4 y | 10^th^ thoracic vertebral body | *L, 1* | **203** Carbonell et al ., (2006) |
| *Maxillary osteoma* | B | 1 | *f*, old | right maxilla, eye | *L, 2* | **9** Siddiqui and Telfah., (2010) |
| *Conventional ameloblastoma* | B | 1 | *f, 5 y* | Right maxilla, sinuses and third molar | *L, 2* | **213** Ibrahim et al., (2023) |
| *Central odontogenic fibroma with ossification* | B | 1 | *f, 8 y* | Right maxillara, | *L, 3* | **205** Ibrahim et al ., (2023) |
| *Intramuscular myxoma* | .B | 1 | *f*, 8 y | mass anterior to hock joint (prim); adjacent and infiltrated to muscles | *L, 2* | **206** Narnaware et al., (2021) |

***Tumor:** B: Benign; M: Malignant

**Differentiation:** *wd=* well differentiated/moderately differentiated; *pd* = poorly differentiated; *an*= anaplastic**.**

****** **Sex:** *m=male, f=female;* **Age:** y= years

***** Organs involved** (name of organ): Prim = Primary lesion; Inva = Invasive lesion; Meta= metastatic lesion

******Lesion** **size**: *L* = Large; *med* =Median; *Sm* =Small, *U*= Undefined.

**Lesion Distribution**: *1*= single lesion in single organ; *2* = single lesion in multiple organs; *3* = multiple lesions in single organ; *4* = multiple lesions in multiple organs, *5*= infiltrative tumor cells

Table S6a: Summary of tumors reported in the female genital system: Tumor classification, tumor benign or malignant and differentiation, No of cases diagnosed, sex and age, organs involved, lesion size and distribution, references and reference number. Asterisks (***** - ********) explained below

| **Tumor Classification** | ***Tumor**  **-** Type**:** B; M  -Differentiation: *wd, pd, an* | **No of**  **cases** | ****Sex:**  *f, m*  **-Age** *y,* | ***** Organs involved** (Prim, Invas, Metast) | ******Lesion**  **-Size:**  *L, Med, S, U*  **-Distribution:** *1, 2, 3, 4, 5* | **References** |
| --- | --- | --- | --- | --- | --- | --- |
| ***Ovaries*** |  |  |  |  |  |  |
| *Granulosa cell tumor* | M, *wd* | 1 | *f,* 14 y | Left ovary | *L, 3* | **232** Ali, et al., (2013) |
| *Adult granulosa cell tumor* | M, *wd* | 1 | *f,*7 y | left ovary with one nodule | *S,1* | **11** Elmaghraby et al., (2023). |
| *Dysgerminoma* | B | 1 | *f,*>16 y | Left ovary | *S, 1* | **207** El-Khouly, et al., (1991) |
| *Arrhenoblastoma (Sertoli-ledig cell tumor group)* | B | 2 | *f,*>16 y | (1) in Left and (1) in right ovary | *S, 1* | **207** El-Khouly, et al., (1991) |
| *Arrhenoblastoma (Sertoli-ledig cell tumor group)* | B | 1 | *f,* adult | Nodules attached to ovary | *S, 3* | **150** Shawky, et al., (2004) |
| *Interstitial cell tumor leydig* | B | 1 | *f,* 12 y | left ovary with one nodule | *S, 1* | **11** Elmaghraby et al., (2023). |
| *Sertoli cell tumor* | B | 1 | *f,*>16 y | Right ovary | *L, 1* | **207** El-Khouly, et al., (1991) |
| *Sertoli-Leydig cell tumor* | B | 1 | *f,8 y* | Right ovary depicted Sertoli-Leydig cell | *L, 1* | **7** Ali, et al., (2019) |
| *Steroid cell tumor-NOS* | B | 1 | *f,*8 y | left ovary nodules embedded in ovarian tissue | *S, 3* | **11** Elmaghraby et al., (2023) |
| *Papillary adenoma* | B | 8 | *f,*>16 y | (4) tumors in Left and (4) in right ovary | *S-Med, 1* | **207** El-Khouly, et al., (1991) |
| *Ovarian adenoma* | B | 1 | *f,* adult | ovary | *S-Med, 3* | **223** Hamouda, Al-Hizab and Hasseeb (2011) |
| *Cystadenoma* | B | 1 | *f,* adult | ovary | *U, 1* | **237** Wajid (2015) |
| *Papilliferous cystadenoma* | B | 1 | *f,* adult | ovary | *U, 1* | **237** Wajid (2015) |
| *Thecoma or fibrothecoma* | B | 2 | *f*,5 y  *f,*,11 y | left ovary with multiple nodule | *S, 3* | **11** Elmaghraby et al., (2023). |
| *Granulosa-theca cell tumor/mixed sex cord stromal tumors* | B | 10 | *f*, 5-11 y | Ovaries with nodules and cysts | *S, 3* | **11** Elmaghraby et al., (2023). |
| *Teratomas* | B | 10 | *f,*>16 y | Ovary (unilateral/ bilateral cysts) | *L- Med-S, 3* | **216** El-Khouly, et al., (1991) |
| *Teratoma* | B | 1 | *f, adult* | ovary | *U* | **224** Abd El-Wahab., (1991). |
| *Teratoma* | B | 2 | *f,* adult | Ovary | *L, 1* | 150 Shawky, et al., (2004) |
| *Teratoma* | B | Not specified | *f, adult* | Ovary | *U* | **226** El-Wishy (1990) |
| *Teratomas* | B | specified | *f, adult* | Ovary | *U* | **221** El Wishy (1992) |
| *Cystic teratoma (dermoid cyst)* | B | 1 | *f,* adult | right ovarian cortex | *L, 1* | 225 Tafti and Nourani (2004) |
| *Teratomas* | B | ? | *f,* adult | ovary | *U, 1* | **237** Mesbah et al., (2002) |
| *Teratomas* | B | 4 | *f, adult* | ovary | *S, 1* | **222** El Wishy (2007) |
| *Teratoma* | B | 1 | *f,* adult | ovary | *Med, 1* | **223** Hamouda, Al-Hizab and Hasseeb (2011) |
| *Teratoma* | B | 6 | *f*, adult | ovarian surface | *S, Med, 3* | **208** Al Afaleq, et al., (2012) |
| *Teratoma* | B | 3 | *f*, adult | ovarian surface | *S, Med, 3* | **228** Al Afaleq, et al., (2022) |
| *Teratomas* | B | 1 | *f*, adult | ovary | *U, 1* | **220** Gherissi et al., (2019) |
| *Ovarian fibroma* | B | 4 | *f*, adult | ovary surface | *S, Med, 3* | **208** Al Afaleq, et al., (2012) |
| *Ovarian fibroma* | B | 2 | *f*, adult | ovary surface | *S, Med, 3* | **228** Al Afaleq, et al., (2022) |
| *Ovarian hemangioma* | B | 1 | *f*, adult | ovary surface | *S, Med, 1* | **208** Al Afaleq, et al., (2012) |
| *Ovarian hemangioma* | B | 1 | *f*, adult | ovary surface | *S, Med, 1* | **228** Al Afaleq, et al., (2022) |
| *Ovarian hemangioma* | B | 2 | *f*, adult | Left ovarian surface | *S, Med, 1* | **238** Melaku et al., (2015) |
| ***Uterus*** |  |  |  |  |  |  |
| *Cystic endometrial adenocarcinoma* | M, *pd* | 2 | *f*, adult | Uterine horns | *L, 3* | **150** Shawky, et al., (2004) |
| *Endometrial adenocarcinoma* | M, *pd* | 1 | *f,* >15 y | Primary: Uterus metastasis: salpinx, urinary bladder, peritoneum, blood and lymphatic vessels, | *S- Med, , 4* | **208** Al Afaleq, et al., (2012) |
| *Adenocarcinoma* | M, *wd* | 1 | *f,* adult | Endometrium with myometrium invasion | *S-Med, 1* | **161** Ali, et al., (2018) |
| *Leiomyoma* | B | 2 | *f,* adult | Mass attached to uterine wall | *L, 1* | **150** Shawky, et al., (2004) |
| *Lipoma* | B | 1 | *f,* adult | mass attached to uterine serosa | *L, 1* | **150** Shawky, et al., (2004) |
| *Uterine cavernous hemangioma* | B | 8 | *f,* adult | endometrium Blood vessels | *U, 3* | **208** Al Afaleq, et al., (2012) |
| ***Vagina & cervix*** |  |  |  |  |  |  |
| *Adenocarcinoma* | M, *wd* | 1 | *f,* adult | Vaginal wall | *S-Med-L; 3* | **24** Ali, et al (2010) |
| *Adenocarcinoma* | M, *wd* | 9 | *f,* 9-13 y | Vagina, one case metastasized to live, mesentery and lymph node, | *S-Med; 1-4* | **13** Ali, et al., (2018) |
| *Adenocarcinoma* | M, *wd* | 2 | *f,* 9-13 y | cervix | *S-Med; 3* | **13** Ali, et al., (2018) |
| *Lipoma* | B | 1 | *f,* 4-15 y | Vaginal wall | *L, 1* | **23** Ali, et al., (2010) |
| *Lipoma* | B | 1 | *f, adult* | ventral commissars of the vulva | *L, 1* | **9** Siddiqui and Telfah (2010) |
| ***Mammary gland*** |  |  |  |  |  |  |
| *Simple tubular mammary adenocarcinoma* | M, *wd* | 1 | *f, adult* | Mammary gland | *mass, 1* | **209** Hegazy et al., (2004) |
| *Mammary Adenocarcinoma* | M | 20 | *f*, 5-15 y | Mammary gland | *U* | **116** Abeer et al., (2016) |
| *Intra-cystic papillary carcinoma* | M, *pd* | 1 | *f*, 13 y | Mammary gland, *inv* | *L, 1* | **210** Abeer et al., 2019 |
| *Mammary carcinoma* | M, *pd* | 1 | *f,* 15 y | mammary gland (prim). lungs and pulmonary lymph nodes (metast) | *L, 2* | 178 Bryant, Portas and Montali (2007) |
| *Papillary carcinoma and fibroadenoma* | M  B | 1 | *f*, 9 y | Mammary gland | *L-Med, 1* | **116** Abeer et al., (2016) |
| *Intraductal fibroma* | B | 3 | *f,* adult | Mammary glands | *U, 1* | **209** Hegazy et al., (2004) |
| *Fibroadenoma* | B | 8 | *f,*5-15 y | Mammary gland | *L, 1* | **116** Abeer et al., (2016) |
| *Cystic adenomatous hyperplasia* | B | 15 | *f*,5-15 y | Mammary gland | *U, 1* | **116** Abeer et al., (2016) |
|  |  |  |  |  |  |  |

***Tumor:** M: Malignant; B benign

**Differentiation:** *wd=* well differentiated/moderately differentiated; *pd* = poorly differentiated; *an*= anaplastic**.**

****Sex:** female **♀**, male *Y,* not specified *NS*

***** Organs involved** (name of organ): Prim = Primary lesion; Inva = Invasive lesion; Meta= metastatic lesion

******Lesion** **size**: *L* = Large; *med* =Median; *S* =Small. *U*= Undefined

**Lesion Distribution**: *1*= single lesion in single organ; *2* = single lesion in multiple organs; *3* = multiple lesions in single organ; *4* = multiple lesions in multiple organs; *5*= infiltrative tumor cells

Table S6b: Summary of tumors reported in the male genital system of the dromedary camel Tumor classification, tumor benign or malignant and differentiation, No of cases diagnosed, sex and age, organs involved, lesion size and distribution, references and reference number. Asterisks (***** to ********) explained below

| **Tumor Classification** | ***Tumor**  **-**Type: B; M  -Differentiation: *wd, pd, an* | **No of**  **cases** | ****Sex:** *m, f*  *-* **Age:** *y* | ***** Organs involved** (Prim, Invas, Metast) | ******Lesion**  **-Size:** *L, Med, S, U*  **-Distribution** *1, 2, 3, 4* | **References** |
| --- | --- | --- | --- | --- | --- | --- |
| ***Testicles*** |  |  |  |  |  |  |
| *Sertoli cell tumors* | B | 1 | *m***,** | testicles | *U, 1* | **211** Hemeida et al. (1985) |
| *Leydig (interstitial) cell tumor* | B | 1 | *m,* | Testicle, unilateral cryptorchidism | *U, 1* | **234** El-Hariri & Deed (1979). |
| *Seminomas* | M | 1 | *m***,** a*dult* | testicle | *U, 1* | **211** Hemeida et al., (1985) |
| *Seminoma*  *(diffuse), and with cholangiocarcinoma* | M  M | 1 | *m***,** *18 y* | right testicle  Liver | *Med- L, 2* | **154** Birincioglu, et al., (2008), |
| *Seminoma*  *(diffuse)* | M, *wd* | 1 | *m***,** *10 y* | right testis | *L, 1* | **212** Ali, et al., (2013) |
| *Different types of male tumors* | M, B | - | *m***,** undefined | Prepuce | *S- L,* | **9** Siddiqui and Telfah (2010) |

***Tumor:** M: Malignant, B: benign

**Differentiation:** *wd=* well differentiated/moderately differentiated; *pd* = poorly differentiated; *an*= anaplastic**.**

****** **Sex:** *m=male, f=female;* **Age:** y= years

***** Organs involved** (name of organ): Prim = Primary lesion; Inva = Invasive lesion; Meta= metastatic lesion

******Lesion** **size**: *L* = Large; *med* =Median; *S* =Small. *U*= Undefined

**Lesion Distribution**: *1*= single lesion in single organ; *2* = single lesion in multiple organs; *3* = multiple lesions in single organ; *4* = multiple lesions in multiple organs; *5*= infiltrative tumor cells

Table S7: Summary of tumors reported in the urinary system of the dromedary camel: Tumor classification, tumor benign or malignant and differentiation, No of cases diagnosed, sex and age, organs involved, lesion size and distribution, references and reference number. Asterisks (***** -********) explained below

| **Tumor Classification** | ***Tumor**  **-**Type: B; M  -Differentiation *wd, pd, an* | **No of**  **cases** | ****Sex:**  *m, f*  **Age:** y | ****** ***Organs**  **involved** (Prim, Invas, Metast) | ******Lesion**  **-Size***: L, Med, S, U*  **-Distribution:** *1, 2, 3, 4* | **References** |
| --- | --- | --- | --- | --- | --- | --- |
| *Renal cell carcinoma* | M | 1 | -, - | Right kidney (*prim)* | *L*, *1* | **243** Vitovec (1982) |
| *Renal cell carcinoma* | M, *wd* | 1 | *f,* | Kidney *(prim)* | *L*, *1* | **10** Alsobayil et al., (2018) |
| *renal cell carcinoma* | M, *an* | 1 | *f,* 13 *Y* | right kidney | Very L, *1* | **244** Tharwat et al., (2017) |
| *Kidneys: Multicentric T-cell lymphoma* | M, *wd* | 1 | *f*, 7 *Y* | Both kidneys (*meta)* | *L*, *2* | **163** Simmons et al., (2005) |
| *Nephroblastoma* | M, *an* | 1 | *m*, 1/2 *Y* | Left kidney | *L*, *1* | **245** Hazarika, et al., (2011) |
| *Abdominal leiomyoma* | B | 1 | *m*, 2 *Y* | left kidney, spleen left abdominal wall, blood vessels | *Very L, 4* | **169** Sadan et al., (2024) |
| *Renal cell adenoma* | B | 1 | *Not specified* | Kidney | *S*, *1* | **248** Rezaie, et al (2014) |
| *Urinary Bladder: Adenocarcinoma* | M, *pd* | 1 | *f*, >15 *Y* | *met* from endometrium to: Urinary bladder, salpinx, peritoneum, blood vessels, lymphatics, | *S*, *4* | **208** Al Afaleq, et al., (2012) |
| *Fibroma* | B | 1 | Not specified | Urinary bladder neck | *U* | **250** Choudhry et al., (1995) |
| *Urethra:* Fibroma | B | 1 | *m* | urethra | *U* | **170** Gahlot et al ., (1995) |

***Tumor:** B: Benign; M: Malignant

**Differentiation:** *wd=* well differentiated/moderately differentiated; *pd* = poorly differentiated; *an*= anaplastic**.**

****** **Sex:** *m=male, f=female;* **Age:** y= years

***** Organs involved** (name of organ): Prim = Primary lesion; Inva = Invasive lesion; Meta= metastatic lesion

******Lesion** **size**: *L* = Large; *med* =Median; *S* =Small, *U*= Undefined.

**Lesion Distribution**: *1*= single lesion in single organ; *2* = single lesion in multiple organs; *3* = multiple lesions in single organ; *4* = multiple lesions in multiple organs; *5*= infiltrative tumor cells

Table S8: Summary of tumors reported in Nervous system of the dromedary camel: Tumor classification, tumor benign or malignant and differentiation, No of cases diagnosed, sex and age, organs involved, lesion size and distribution, references and reference number. Asterisks (***** -********) explained below

| **Tumor Classification** | ***Tumor**  **-**Type: B; M  -Differentiation: *Wd, pd, an* | **No of cases** | ****Sex:**  *m, f*  **Age:** *y* | ******* **Organs**  **involved** (Prim, Invas, Metast) | ******Lesion**  **-Size***: L, Med, S, U*  **-Distribution:** *1, 2, 3, 4* | **References** |
| --- | --- | --- | --- | --- | --- | --- |
| *Primitive Neuroectodermal tumor* | M, *an* | 1 | *m*, 9 y | -3^rd^lumber vertebra,  -liver | *U, 4* | **166** Weiss, Walz., (2009) |
| *Choroid plexus papilloma* | B | 1 | *m*, 6 y | 4th ventricle, hydrocephalus, cavities in brain stem and cerebral hemispheres | *S, 1* | **251** Ahmed et al., (2016) |
| *Multicentric Schwannoma* | B | 1 | 4 y | 1: peripheral nerve sheath (prim)  2: metastasis: see digestive system | *S, 4* | **167** Khodakaramp-Tafti & Khordadmehr., (2010) |
| *Schwannoma* | B | 1 | Not specified | peripheral nerve sheath | *U* | **10** Alsobayil et al., (2018) |

***Tumor:** B: Benign; M: Malignant

**Differentiation:** *wd=* well differentiated/moderately differentiated; *pd* = poorly differentiated; *an*= anaplastic**.**

****** **Sex:** *m=male, f=female;* **Age:** y= years

***** Organs involved** (name of organ): Prim = Primary lesion; Inva = Invasive lesion; Meta= metastatic lesion

******Lesion** **size**: *L* = Large; *med* =Median; *S* =Small, *U*= Undefined

**Lesion Distribution**: *1*= single lesion in single organ; *2* = single lesion in multiple organs; *3* = multiple lesions in single organ; *4* = multiple lesions in multiple organs; *5*= infiltrative tumor cells

Table S9a: Summary of tumors reported in the skin and appendages: Tumor classification, tumor benign or malignant and differentiation, No. of cases diagnosed, sex and age, organs involved, lesion size and distribution, references and reference number. Asterisks (***** to ********) explained below

| **Tumor Classification** | ***Tumor**  **-** Type**:** B; M  - Differentiation: *wd, pd, an* | **No of**  **cases** | ****Sex:**  *m,* *f*  **Age:** *y* | ***** Organs involved** (Prim, Invas, Metas) | ******Lesion**  **-Size:**  *L, Med, S, U*  **-Distribution :** *1, 2, 3, 4* | **References** |
| --- | --- | --- | --- | --- | --- | --- |
| ***Skin tumors:*** *undefined location* |  |  |  |  |  |  |
| *Melanoma* | M, *an* | 2 | *Sex not specified, adult* | Skin (epidermal Hyperpigmented nodules) | *L-med, 1* | **114** Khordadmehr et al ., (2016) |
| *Mast cell tumor mastocytoma* | M | 1 | *Not specified* | Skin | *U* | **191** Abubakr et al., (1998) |
| *Squamous papilloma* | B | 4 | *Sex not specified, adult* | skin (Keratinized ulcerated epidermis) | *U, 1* | **114**Khordadmehr et al., (2016) |
| *Fibropapilloma* | B | 4 | *Sex not specified, adult* | Skin nodules | *L-med,1* | **114**Khordadmehr et al., (2016) |
| *Lipoma* | B | 2 | *Sex not specified, adult* | Skin (dermal masses) | *U,1* | **114**Khordadmehr et al., (2016) |
| *Melanocytoma* | B | 2 | *Sex not specified, adult* | Skin | *L-med,1* | **114**Khordadmehr et al., (2016) |
| *Sebaceous gland adenoma* | B | 1 | *Sex not specified, adult* | Skin raised masses (Sebaceous glands) | *U, 1* | **114**Khordadmehr et al., (2016) |
| *Sebaceous ductal adenoma* | B | 1 | *Sex not specified, adult* | Skin ( sebaceous glands ducts) | *U, 1* | **114**Khordadmehr et al., (2016) |
| *Fibroma* | B | 2 | *Sex not specified,* | Skin (subcutaneous tissue) | *U* | **191**Abubakr et al., (1998) |
| ***Skin tumors:***  ***Head, neck and chest*** |  |  |  |  |  |  |
| *Basal cell carcinoma* | M, *wd* | 1 | *f*, 10 y | proximal part of neck from facial crest to medial canthus, attached to skin (dermis, subcutis), involved maxilla sinuses, lymph nodes, | *L, 2* | **259** Al Hizab, et al., (2007) |
| *Myxosarcoma* | M, *pd* | 1 | *m,adult* | Skin of chest | *L, 1* | **257** Elmaghraby et al., (2023) |
| *Osteosarcoma* | M | 1 | *not specified* | Skin swelling at ventral to mandible, | *U,1* | **186** El-Shafaey et al., (2020) |
| *Squamous cell carcinomas* | M | 2 | *not specified* | Skin swelling at maxilla and neck regions, | *U,1* | **186** El-Shafaey et al., (2020) |
| *Lymphosarcoma* | M | 1 | *not specified* | Skin swelling at neck region | *U,1* | **186** El-Shafaey et al., (2020) |
| *Melanoma* | M, *an* | 1 | *m, 21 y* | Skin of neck | *Med, 1* | **261** Al-Ani, et al., (1998) |
| *Fibromas* | B | 2 | *Sex not specified*, *adult* | chest pad | *L, 1* | **9** Siddiqui and Telfah (2010) |
| *Fibroma* | B | 3 | *f, adult* | neck | *L, 1* | **6** Al-Sobayil and El-Amir (2013) |
| *Papillomatosis* | B | 3 | *Sex not specified,*  1-3 y | Dry crusty surface pedunculated lesions in and around mouth | *S, 1* | **128** Kinne and Wernery (1998) |
| *Papillomatosis* | B | 8 | *m, f* ; young < 2y | Skin of lips and jaw | *S, 1* | **19**Khalafalla et al., (1998) |
| *Papillomatosis* | B | 20 | *m, f* ; 3 to 9 months | cauliflower-like nodules; face, upper and lower lips | *S, 3* | **129** Barakat, et al., (2013) |
| *Fibropapillomas* | B | 25 | *m, f* ; 3-7 months | lips and lower Jaw. Raised nodule & cauliflower-like warts. | *S, 4* | **21** Ure, et al., (2011) |
| *Pleomorphic adenoma eccrine gland type* | B | 1 | *m,*12 y | sweat gland lateral to left masseter region | *Med-L, 1* | **265** Ramadan et al., (2016) |
| *Sweat gland adenoma* | B | 1 | *m, adult* | Skin of neck | *L, 1* | **257** Elmaghraby et al., (2023) |
| *Pyogranuloma* | B | 1 | *not specified* | Skin swelling at retropharyngeal region, | *U,1* | **186** El-Shafaey et al., (2020) |
| *Fibromas* | B | 2 | *not specified* | Skin swelling at chest pad, and neck) | *U,1* | **186** El-Shafaey et al., (2020) |
| *Teratoma* | B | 11 | *m, f adult* | 9 single and two bilateral at proximal neck | *L, 3* | **267** Purohit et al., (1989) |
| *Teratoma* | B | 1 | *f, 2 y* | proximal part of neck | *L, 1* | **264** Oryan et al., (2012) |
| *Teratoma* | B | 10 | *m, f* | proximal part of neck | *L, 3* | **266** Singh et al., (2021) |
| ***Skin tumors :*** *Thorax, Abdumen, fore and hind quarters*, joints, perineum and tail |  |  |  |  |  |  |
| *Squamous cell carcinoma* | M, *pd* | 1 | *m*, 9 y | Skin of Left fore-limb above coronet | *L, 1* | **254** Tageldin and Omer, (1986) |
| *Squamous cell carcinoma* | M, *wd and pd* | 11 | *f, adult* | Ventral abdominal wall (7), ventrolateral of thigh (n=1), sacral area (n=3), | *L, 1* | **6** Al-Sobayil and El-Amir (2013) |
| *Squamous cell carcinoma of skin* | M**,** *wd* | 1 | *m*, 7 y | Skin of right hock joint (prim); tarsal & metatarsal bones (inv) | *L, 3* | **202** Abelardo Morales-Briceño et al., (2017) |
| *1: Squamous cell carcinoma*  *2: Sarcoma* | M, *wd*  M, *pd* | 1 | *m*, 20 y | -wound at crush pad (prim)  -pre-scapular lymph node (inv), -subcutaneous tissue (inv)  2:pedunculated mass | *L, 3*  *Med, 1* | **258**Garcia et al., (2022) |
| *Myxosarcoma* | M, *pd* | 6 | *m,4-10 y* | 3(skin of scrotum), 2 in forelimb, and 1 in back | *L, 1* | **257**Elmaghraby et al., (2023) |
| *Fibrosarcoma* | M | 1 | *Sex and age not specified* | Skin swelling at ventrolateral of abdomen | *U, 1* | **186** El-Shafaey et al., (2020) month 11y |
| *Melanoma* | M, *an* | 1 | *m, 21 y* | Skin of neck | *Med, 1* | **261** Al-Ani, et al., (1998) |
| *Papillomatosis* | B transforming into M | 1 | *f*, 15 y | wart-like growth over right fetlock joint | *L, 1* | **17** Sadana et al., (1980) |
| *Papillomatosis* | B | Many (outbreak) | *m***,** *f*; 6 months- 2 y | pustules & scabs on lips & nostrils. Generalized nodules (legs, ear, eyelids, inguinal, genital regions). | *L – S, 4* | **18** Munz et at., (1990) |
| *Papillomatosis* | B | Many (outbreak) | *m***,** *f*; young | Skin of mouth and nostrils | *L – Med-S, 1* | **354** Dioli and Stimmelmayr (1992) |
| *Fibropapilloma* | B | 2 | *18 & 24 Months* | mass on the metatarsal region | *L, 1* | **269** Oryan, et al., (2011) |
| *Fibroma* | B | 1 | *m, 8 y* | right metatarsal area | *L, 1* | **9** Siddiqui and Telfah (2010) |
| *Fibroma* | B | 7 | *f, adult* | thigh (6 cases) and fetlock joint (1 case) | *L, 1* | **6** Al-Sobayil and El-Amir (2013) |
| *Lipoma* | B | 1 | *f, adult* | hock joint | *L;1* | **6** Al-Sobayil & El-Amir (2013) |
| *Lipoma* | B | 1 | *m,* 7 y | left ischiorectal fossa | *L, 1* | **174** Kaswan et al., (2013) |
| *Lipoma* | B | 1  1 | *m,* 9 y  *f,* 10 y | Skin of scrotum, Skin of ventral abdominal wall | *L, 1*  *L, 1* | **257** Elmaghraby et al., (2023) |
| *Lpomas* | B | 1 | *f,* 10 y | Two bilateral very big s/c masses above base of tail | *L; 3* | **271** Zabady et al., (2024) |
| *Intramuscular myxoma* | B | 1 | *m,*, 8 y | Cutaneous mass anterior to hock joint (prim); adjacent muscles (inva) | *L, 2* | **206**Narnaware et al., (2021) |
| *Fibromas* | B | 6 | *Sex and age not specified* | Skin swelling at forelimb (1), hind limbs (4), abdomen (1) | *U, 1* | **186** El-Shafaey et al., (2020) |
| *Papilloma* | B | 1 | *Sex and age not specified* | Skin swelling at ventral abdomen, | *U, 1* | **186** El-Shafaey et al., (2020) |
| *Myxopapilloma* | B | 1 | *Sex and age not specified* | Skin swelling at ventral abdomen, | *U, 1* | **186** El-Shafaey et al., (2020) |
| *Squamous cell papilloma* | B | 1 | *m,* 10 y | Skin of scrotum | *U, 1* | **257** Elmaghraby et al., (2023) |
| *Fibropapilloma* | B | 2 | 18 months, 24 months | metatarsal region | *L.1* | 269 [Oryan](https://www.researchgate.net/profile/Ahmad-Oryan?_sg%5B0%5D=vy2DYEAFbcT5uNAQO-I_aJtsYQln0S5XhwPpHqRQ45jsd29VZUkzuhnEChFvSC6VK8EwnrY.k_tBzpVFGli9_GNbkOGR3Uh349UGQe8Xp1zNtkSNYo7cUzIeoUgH-vDJAGGBrYOfMjSTg1VCOSNLCs_eJQcVBA&_sg%5B1%5D=DSuq_fdZa262-trKKy-MV58UKdmrbWzLwcPpnc4u4ox4hbGZDqCsx8PV9vliiPg8az4A9eM.GIteTY36EIh-rtdYv6mHvASqKIgS7uzXF7apsIEUHeajXOc32o2BUZI17-MQoMK8cwxSohNIP7EBB34HJrgLUA&_tp=eyJjb250ZXh0Ijp7ImZpcnN0UGFnZSI6InB1YmxpY2F0aW9uIiwicGFnZSI6InB1YmxpY2F0aW9uIiwicHJldmlvdXNQYWdlIjoicHVibGljYXRpb24iLCJwb3NpdGlvbiI6InBhZ2VIZWFkZXIifX0) (2011) |
| *Fibropapilloma* | B | 1 | *m,* 5 y | Skin of forelimb | *, 1* | **257**Elmaghraby et al., (2023) |
| *Dermoid cyst* | B | 1 | *m, adult* | left carpal joint and fetlock joints | *Med-L, 2* | **9** Siddiqui and Telfah (2010) |
| ***Skin tumors: Toe*** |  |  |  |  |  |  |
| *Squamous cell carcinoma* | M, *wd* | 35 | *M, f* | toenails of forelimbs and hind limbs | *L-Med-S, 1* | **255** Siddiqui, et al., (2013) |
| *Squamous cell carcinoma (SCC)* | M | 114 | *m, f* | toenails of forelimbs and hind limbs | *L-Med-S, 1* | **256** Baker et al., (2017) |
| *Spiny keratoderma* | B | 11 | *m, f* | toenails of fore and hind limbs | *L-Med-S, 1* | **255** Siddiqui, et al., (2013) |
| *Spiny keratoderma* | B | 4 | *m, f* | toenails of forelimbs and hind limbs | *L-Med-S, 1* | **256** Baker et al., (2017) |
| *Fibroma* | B | 32 | *m, f* | toenails of forelimbs and hind limbs | *L-Med-S, 1* | **256** Baker et al., (2017) |
| *Fibroma* | B | 4 | *m, f* | toenails of forelimbs and hind limbs | *L-Med-S, 1* | **255** Siddiqui, et al., (2013) |

Table S9b: Summary of tumors reported in the eye of the dromedary camel: Tumor classification, tumor benign or malignant and differentiation, No of cases diagnosed, sex and age, organs involved, lesion size and distribution, references andreference number. Asterisks (***** - ********) explained below

| **Tumor Classification** | ***Tumor**  **-**Type: B; M  -Differentiation *wd, pd, an*) | **No of**  **cases** | ****Sex:**  *m, f*  **Age:** *y* | *****Organs involved** (Prim, Invas, Metast) | ******Lesion**  **-Size***: L, Med, S, U*  **-Distribution:** *1, 2, 3, 4 , 5* | **References** |
| --- | --- | --- | --- | --- | --- | --- |
| *Sebaceous carcinoma* | M | 1 | *m*, adult | Eyelid (skin) | *U, 1* | **260** Al Sadi and Obidi (2006) |
| *Squamous cell carcinoma* | M, *wd* | 2 | Not specified | Unilateral, around the cornea of right and of left eyes | *Med, 1* | **143**Abdella et al., (2018) |
| *Disseminated gamma‐delta T‐cell lymphoma* | M | 1 | *f*, 12 y | Right eye, liver, mesenteric lymph nodes | *L,4* | **165** Ibrahim et al., (2023) |
| *Unclassified ocular tumor* | - | 1 | Not specified | Left eye, Unilateral hyperechoic intraocular mass | *U, 1* | **272** El-Tookhy and Tharwat (2012) |
| *Uveal melanoma* | B | 1 |  | internal surfaces of iris and cornea and the ciliary body | *U, 1* | **209** Higazy, et al., (2004) |
| *Corneal papilloma* | B | 1 | *m*, 15 y | solid, pedunculated corneal nodule in the left eye, | *S, 1* | **130** Kilic et al., (2010) |
| *Corneal dermoid cyst* | B | 1 | *m*, 10 y | nodule on cornea of left eye | *L,1* | **270** Tmumen (2019) |
| *Maxillary osteoma, with eye involvement* | B | 1 | *f*, aged | right maxilla, right eye | *L, 2* | **9** Siddiqui and Telfah (2010) |

***Tumor**: M: Malignant; B benign

**Differentiation:** *wd=* well differentiated/moderately differentiated; *pd* = poorly differentiated; *an*= anaplastic**.**

****Sex:** female **♀**, male *Y,* not specified *NS*

***** Organs involved** (name of organ): Prim = Primary lesion; Inva = Invasive lesion; Meta= metastatic lesion

******Lesion** **size**: *L* = Large; *med* =Median; *S* =Small. *U*= Undefined size

**Lesion Distribution**: *1*= single lesion in single organ; *2* = single lesion in multiple organs; *3* = multiple lesions in single organ; *4* = multiple lesions in multiple organs; *5*= infiltrative tumor cells

Figure S1 frequencies of malignant and benign tumors in different body systems of the dromedary camels. Cumulative frequency (added numbers of X-axis at the end of horizontal bars) and relative frequencies (% numbers) at Y-axis below each body system. The total tumor data (682) were collated from individual studies from different authors at different times from different places

Figure S2: Relative frequencies of malignant tumors (X-axis) and tumor names (Y-axis) in different body systems of the dromedary camels. The total tumor data (682) were collated from individual studies from different authors at different times from different places

Figure S3: Relative frequencies of benign tumors (X-axis) and tumor names (Y-axis) in different body systems of the dromedary camels. The total tumor data (682) were collated from individual studies from different authors at different times from different places
